# CDC Clinical Practice Guideline for Prescribing Opioids for Pain **—** United States, 2022

**DOI:** 10.15585/mmwr.rr7103a1

**Published:** 2022-11-04

**Authors:** Deborah Dowell, Kathleen R. Ragan, Christopher M. Jones, Grant T. Baldwin, Roger Chou

**Affiliations:** ^1^Division of Overdose Prevention, National Center for Injury Prevention and Control, CDC; ^2^Office of the Director, National Center for Injury Prevention and Control, CDC; ^3^Pacific Northwest Evidence-based Practice Center and Oregon Health & Science University, Portland, Oregon

## Abstract

*This guideline provides recommendations for clinicians providing pain care, including those prescribing opioids, for outpatients aged ≥18 years. It updates the *CDC Guideline for Prescribing Opioids for Chronic Pain — United States, 2016 (MMWR Recomm Rep 2016;65[No. RR-1]:1–49) *and includes recommendations for managing acute (duration of <1 month), subacute (duration of 1–3* *months), and chronic (duration of >3 months) pain. The recommendations do not apply to pain related to sickle cell disease or cancer or to patients receiving palliative or end-of-life care. The guideline addresses the following four areas: 1) determining whether or not to initiate opioids for pain, 2) selecting opioids and determining opioid dosages, 3) deciding duration of initial opioid prescription and conducting follow-up, and 4) assessing risk and addressing potential harms of opioid use. CDC developed the guideline using the Grading of Recommendations Assessment, Development, and Evaluation (GRADE) framework. Recommendations are based on systematic reviews of the scientific evidence and reflect considerations of benefits and harms, patient and clinician values and preferences, and resource allocation. CDC obtained input from the Board of Scientific Counselors of the National Center for Injury Prevention and Control (a federally chartered advisory committee), the public, and peer reviewers. CDC recommends that persons with pain receive appropriate pain treatment, with careful consideration of the benefits and risks of all treatment options in the context of the patient’s circumstances. Recommendations should not be applied as inflexible standards of care across patient populations. This clinical practice guideline is intended to improve communication between clinicians and patients about the benefits and risks of pain treatments, including opioid therapy; improve the effectiveness and safety of pain treatment; mitigate pain; improve function and quality of life for patients with pain; and reduce risks associated with opioid pain therapy, including opioid use disorder, overdose, and death.*

## Introduction

### Background

Pain is one of the most common reasons adults seek medical care in the United States (*1*). Acute pain, a nearly universal experience, is a physiologic response to noxious stimuli that can become pathologic. Acute pain is usually sudden in onset and time limited (defined in this clinical practice guideline as having a duration of <1 month) and often is caused by injury, trauma, or medical treatments such as surgery (*2*,*3*). Unresolved acute pain or subacute pain (defined in this clinical practice guideline as pain that has been present for 1–3 months) can evolve into chronic pain (*4*). Chronic pain typically lasts >3 months (*4*) and can be the result of an underlying medical disease or condition, injury, medical treatment, inflammation, or unknown cause (*2*). Approximately one in five U.S. adults had chronic pain in 2019 and approximately one in 14 adults experienced “high-impact” chronic pain, defined as having pain on most days or every day during the past 3 months that limited life or work activities (*5*). Pain, especially chronic pain, can affect almost every aspect of a person’s life, leading to impaired physical functioning, poor mental health, and reduced quality of life, and contributes to substantial morbidity each year (*6*). In 2011, the economic costs of chronic pain were estimated to range from $560 to $635 billion in annual direct medical costs, lost productivity, and disability (*2*).

Pain is a complex phenomenon influenced by multiple factors, including biologic, psychological, and social factors (*7*). This complexity means substantial heterogeneity exists in the effectiveness of various pain treatments, depending on the type of underlying pain or condition being treated (*7*–*11*). Patients might experience persistent pain that is not well controlled (*6*). Chronic pain often co-occurs with behavioral health conditions, including mental and substance use disorders (*12*,*13*). Patients with chronic pain also are at increased risk for suicidal ideation and behaviors (*14*,*15*). Data from death investigations in 18 states during 2003–2014 indicate that approximately 9% of suicide decedents had evidence of having chronic pain at the time of death; however, this is likely an underestimate because of the limitations of the underlying data sources used in the study (*16*). These factors and potentially harmful outcomes associated with chronic pain for some persons add to the clinical complexity and underscore the importance of adequately treating and providing care to persons with pain. Thus, prevention, assessment, and treatment of pain is a persistent challenge for clinicians. Pain might go unrecognized, and some persons (e.g., members of marginalized racial and ethnic groups; women; older persons; persons with cognitive impairment; persons with mental and substance use disorders, sickle cell disease, or cancer-related pain; and persons at the end of life) can be at risk for inadequate pain treatment (*2*,*6*,*17*–*23*).

Although substantial opportunity exists for improved pain management broadly across the United States, data underscore opportunities for addressing specific, long-standing health disparities (*24*–*26*) in the treatment of pain. For example, patients who identify as Black or African American (Black), Hispanic or Latino (Hispanic), and Asian receive fewer postpartum pain assessments relative to White patients (*27*). Black (*28*,*29*) and Hispanic (*29*) patients are less likely than White patients to receive analgesia for acute pain. Among Black and White patients receiving opioids for pain, Black patients are less likely to be referred to a pain specialist, and Black patients receive prescription opioids at lower dosages than White patients (*24*,*30*). Racial and ethnic differences remain even after adjusting for access-related factors, the needs and preferences of patients, and the appropriateness of the intervention (*25*). These disparities appear to be further magnified for Black and Hispanic patients who live in socioeconomically disadvantaged neighborhoods (*26*). Women might be at higher risk for inadequate pain management (*31*), although they have higher opioid prescription fill rates (*32*) than men at a population level. Geographic disparities contribute to increased use of opioids for conditions for which nonopioid treatment options might be preferred but are less available. For example, adults living in rural areas are more likely to be prescribed opioids for chronic nonmalignant pain than adults living in nonrural areas (*33*). Although not Hispanic or Latino (non-Hispanic) American Indian or Alaska Native and non-Hispanic White populations have experienced much higher rates of prescription opioid–related overdose deaths than non-Hispanic Black, Hispanic, or non-Hispanic Asian or Pacific Islander populations (*34*), application of safeguards in opioid prescribing are disproportionately applied to Black patients. In one study, Black patients were more likely than White patients to receive regular office visits and have restricted early refills (*35*). In another study, clinicians were substantially more likely to discontinue opioids if there was evidence of misuse for Black patients compared with White patients (*36*). Differentially untreated or undertreated pain as a result of clinician biases persists and demands immediate and sustained attention and action (*37*–*40*).

Because of the clinical, psychological, and social consequences associated with pain, including limitations in activities, lost work productivity, reduced quality of life, and pervasive stigma, it is essential that clinicians have the training, education, guidance, and resources to provide appropriate, holistic, and compassionate care for patients with pain (*2*,*6*). An important aim of pain management is the provision of person-centered care built on trust between patients and clinicians. Such care includes appropriate evaluation to identify potentially reversible causes of pain and establish a diagnosis and measurable treatment outcomes that focus on optimizing function and quality of life (*6*). To achieve this aim, it is important that clinicians consider the full range of pharmacologic and nonpharmacologic treatments for pain care, and that health systems, payers, and governmental programs and entities make the full spectrum of evidence-based treatments accessible to patients with pain and their treating clinicians.

The range of therapeutic options has historically been inaccessible to many patients because of factors such as inadequate clinician education, training, and guidance; unconscious bias; a shortage of pain management specialists; insufficient access to treatment modalities such as behavioral therapy; siloed health systems; insurance coverage and reimbursement policies; and lack of clarity about the evidence supporting different pain treatments (*6*,*17*,*41*–*46*). Partly because of these factors affecting access to a wide range of treatment modalities, for many years medications such as prescription opioids have been the mainstay to treat pain, despite very limited evidence to support their long-term (>1 year) benefits; most placebo-controlled trials have been <6 weeks in duration (*2*,*6*,*47*,*48*).

Opioids can be essential medications for the management of pain; however, they carry considerable potential risk. A systematic review published in 2014 by the Agency for Healthcare Research and Quality (AHRQ) found insufficient evidence to demonstrate long-term benefits of prescription opioid treatment for chronic pain, and long-term prescription opioid use was found to be associated with increased risk for overdose and opioid misuse, among other risks (*47*). Some risks, such as overdose, were dose dependent (*47*). In 2014, on the basis of accumulating evidence of potential risks to patients, the Food and Drug Administration (FDA) required new safety labeling changes for extended-release and long-acting opioids. Changes included a boxed warning on the “risks of addiction, abuse, and misuse, which can lead to overdose and death” and, for patients receiving opioids during pregnancy, the risk for neonatal abstinence syndrome (a group of conditions that can occur when newborns withdraw from certain substances including opioids; withdrawal caused by in utero exposure to opioids also is called neonatal opioid withdrawal syndrome) (*49*). In 2016, these warnings were added to the labels for immediate-release opioids (*50*).

In addition to the potential risks to patients, prescribed opioids have the potential for diversion and nonmedical use among persons to whom they were not prescribed (*51*). In the United States, opioid prescribing increased fourfold during 1999–2010; this increase was paralleled by an approximately fourfold increase in overdose deaths involving prescription opioids during the same period (*52*) and increases in prescription opioid use disorder (*53*). In addition to the increased overall volume of opioid prescriptions during this period, how opioids were prescribed also changed; opioids increasingly were prescribed at higher dosages and for longer durations, prescribing behaviors associated with opioid use disorder and overdose (*54*,*55*). The limited evidence of long-term effectiveness of opioids for chronic pain, coupled with risks to patients and to persons using prescription opioids that were not prescribed to them, underscored the importance of reducing inappropriate opioid prescribing while advancing evidence-based pain care to improve the lives of persons living with pain.

CDC recognized the need for a national guideline on pain management that could improve appropriate opioid prescribing while minimizing opioid-related risks and released the *CDC Guideline for Prescribing Opioids for Chronic Pain — United States, 2016* (referred to as the 2016 CDC Opioid Prescribing Guideline hereafter). The 2016 CDC Opioid Prescribing Guideline included 12 recommendations for the prescribing of opioids for chronic pain by primary care clinicians in outpatient settings, excluding active cancer treatment, palliative care, and end-of-life care (*56*). The recommendations in the 2016 CDC Opioid Prescribing Guideline were based on a systematic review of the best-available evidence at the time, along with input from experts and the public and review and deliberation by the Board of Scientific Counselors (BSC) of the National Center for Injury Prevention and Control (NCIPC) (a federally chartered advisory committee). The goals of the guideline were to 1) ensure that clinicians and patients considered safer and more effective pain treatment; 2) improve patient outcomes, such as reduced pain and improved function; and 3) reduce the number of persons who developed opioid use disorder, experienced overdose, or experienced other prescription opioid–related adverse events (*56*). To facilitate uptake and implementation of the 2016 CDC Opioid Prescribing Guideline in clinical practice, CDC used a broad-reaching strategy that included clinician education and training, partnerships with health systems and payers, and multiple clinical tools and fact sheets (*57*).

The number of overall opioid prescriptions in the United States declined after 2012, and further declines have been observed after the release of the 2016 CDC Opioid Prescribing Guideline (*58*). The timing of this release was associated with accelerated decreases in overall opioid prescribing and declines in potentially high-risk prescribing (e.g., high-dosage opioid prescribing and concurrent prescribing of opioid pain medication and benzodiazepines) (*58*,*59*). The release of the 2016 CDC Opioid Prescribing Guideline also was temporally associated with modest increases in the prescribing of nonopioid pain medication (*60*). Although not the intent of the 2016 CDC Opioid Prescribing Guideline, design and implementation of new laws, regulations, and policies also appeared to reflect its recommendations. For example, since 2016, consistent with SUPPORT Act requirements (*61*), some state Medicaid programs have used the guideline and other resources to promote nonopioid options for chronic pain management (*62*). Approximately half of all states have passed legislation limiting initial opioid prescriptions for acute pain to a ≤7-day supply (*63*), and many insurers, pharmacy benefit managers, and pharmacies have enacted similar policies (*64*). At least 17 states have passed laws requiring or recommending the coprescription of naloxone in the presence of overdose risk factors, such as high dosages of opioids or concomitant opioid pain medications and benzodiazepines (*65*).

Although some laws, regulations, and policies that appear to support recommendations in the 2016 CDC Opioid Prescribing Guideline might have had positive results for some patients, they are inconsistent with a central tenet of the guideline: that the recommendations are voluntary and intended to be flexible to support, not supplant, individualized, patient-centered care. Of particular concern, some policies purportedly drawn from the 2016 CDC Opioid Prescribing Guideline have been notably inconsistent with it and have gone well beyond its clinical recommendations (*6*,*66*,*67*). Such misapplication includes extension to patient populations not covered in the 2016 CDC Opioid Prescribing Guideline (e.g., cancer and palliative care patients), rapid opioid tapers and abrupt discontinuation without collaboration with patients, rigid application of opioid dosage thresholds, application of the guideline’s recommendations for opioid use for pain to medications for opioid use disorder treatment (previously referred to as medication assisted treatment), duration limits by insurers and pharmacies, and patient dismissal and abandonment (*66*–*68*). These actions are not consistent with the 2016 CDC Opioid Prescribing Guideline and have contributed to patient harm, including untreated and undertreated pain, serious withdrawal symptoms, worsening pain outcomes, psychological distress, overdose, and suicidal ideation and behavior (*66*–*71*).

### Rationale

Since release of the 2016 CDC Opioid Prescribing Guideline, new evidence has emerged on the benefits and risks of prescription opioids for both acute and chronic pain, comparisons with nonopioid pain treatments, dosing strategies, opioid dose-dependent effects, risk mitigation strategies, and opioid tapering and discontinuation (*7*–*11*). This evidence includes studies on misapplication of the 2016 CDC Opioid Prescribing Guideline (*66*), benefits and risks of different tapering strategies and rapid tapering associated with patient harm (*68*,*71*–*73*), challenges in patient access to opioids (*6*), patient abandonment and abrupt discontinuation of opioids (*71*), a seminal randomized clinical trial comparing prescription opioids to nonopioid medications on long-term pain outcomes (*74*), the association of characteristics of initial opioid prescriptions with subsequent likelihood for long-term opioid use (*75*,*76*), and the small proportion of opioids used by patients compared with the amount prescribed to them for postoperative pain (*77*–*79*).

Opioid medications remain a common treatment for pain despite declines in the number of opioid prescriptions after 2012 (*58*). During 2015–2018, approximately 6% of U.S. adults reported use of one or more prescription opioids during the past 30 days (*80*), and in 2020, approximately 143 million opioid prescriptions were dispensed from pharmacies in the United States (*81*). Rates of opioid prescribing continue to vary across states, medical specialties, patient demographics, and pain conditions in ways that cannot be explained by the underlying health status of the population, and often are discordant with the 2016 CDC Opioid Prescribing Guideline recommendations (*25*,*77*,*82*–*84*). The prevalence of prescription opioid misuse and prescription opioid use disorder also has declined in recent years. In 2019, among persons aged ≥12 years in the United States, 9.7 million reported misuse of prescription opioids during the past year (a decrease from 12.5 million in 2015), and 1.4 million met criteria for a past-year prescription opioid use disorder (a decrease from 2.0 million in 2015) (*85*). However, in 2020, prescription opioids remained the most commonly misused prescription drug in the United States (*51*). Also in 2020, among those reporting misuse during the past year, 64.6% reported the main reason for their most recent misuse was to “relieve physical pain” compared with 11.3% to “feel good or get high” and 2.3% “because I am hooked or have to have it” (*51*). Taken together, these factors underscore the need for an updated clinical practice guideline on appropriate opioid prescribing for pain and pain management.

This clinical practice guideline expands and updates the 2016 CDC Opioid Prescribing Guideline to provide evidence-based recommendations for prescribing opioid pain medication for acute, subacute, and chronic pain for outpatients aged ≥18 years, excluding pain management related to sickle cell disease, cancer-related pain treatment, palliative care, and end-of-life care (Boxes 1 and 2). Lessons learned from the development of the 2016 CDC Opioid Prescribing Guideline informed the process used to generate this update. This update leverages new data to expand content on prescription opioids for acute and subacute pain throughout the recommendations. Importantly, the update also aims to clearly delineate recommendations that apply to patients who are being considered for initial treatment with prescription opioids and patients who have been receiving opioids as part of their ongoing pain management.

BOX 1 **Executive summary of the CDC Clinical Practice Guideline for Prescribing Opioids for Pain — United States, 2022**

This clinical practice guideline updates and expands the *CDC Guideline for Prescribing Opioids for Chronic Pain — United States, 2016* (MMWR Recomm Rep 2016;65[No. RR-1]:1–49]) and provides evidence-based recommendations for primary care and other clinicians (including physicians, nurse practitioners and other advanced practice registered nurses, physician assistants, and oral health practitioners) providing pain care, including those prescribing opioids, for outpatients aged ≥18 years with acute (duration of <1 month) pain, subacute (duration of 1–3 months) pain, or chronic (duration of >3 months) pain. Recommendations on use of opioids for acute pain and on tapering opioids for patients already receiving opioid therapy have been substantially expanded in this update. These recommendations do not apply to patients experiencing pain associated with the following conditions or settings: pain management related to sickle cell disease, cancer-related pain treatment, palliative care, and end-of-life care. Applicable outpatient settings include clinician offices, clinics, and urgent care centers. The recommendations do not apply to providing care to patients who are hospitalized or in an emergency department or other observational setting from which they might be admitted to inpatient care. These recommendations do apply to prescribing for pain management when patients are discharged from hospitals, emergency departments, or other facilities.

This clinical practice guideline addresses the following areas:

1. Determining whether or not to initiate opioids for pain
2. Selecting opioids and determining opioid dosages
3. Deciding duration of initial opioid prescription and conducting follow-up
4. Assessing risk and addressing potential harms of opioid use

CDC developed this clinical practice guideline using the Grading of Recommendations Assessment, Development, and Evaluation (GRADE) framework, and recommendations are made based on a systematic review of the available scientific evidence while considering benefits and harms; values and preferences of patients, caregivers, and clinicians; and resource allocation (e.g., costs to patients or health systems, including clinician time). CDC obtained input on this clinical practice guideline through individual conversations with patients, caregivers, and clinicians and public comment opportunities available via *Federal Register* notices. CDC also sought input from the Board of Scientific Counselors of the National Center for Injury Prevention and Control (BSC/NCIPC) (a federally chartered advisory committee), federal partners, and peer reviewers with scientific and clinical expertise.

The clinical evidence reviews found that a number of nonpharmacologic treatments and a number of nonopioid medications are associated with improvements in pain, function, or both, that appear comparable to improvements associated with opioid use. Multiple noninvasive nonpharmacologic interventions (e.g., exercise and psychological therapies) are associated with improvements in pain, function, or both, that are sustained after treatment and are not associated with serious harms. Nonopioid drugs, including serotonin and norepinephrine reuptake inhibitor (SNRI) antidepressants, pregabalin and gabapentin, and nonsteroidal anti-inflammatory drugs (NSAIDs), are associated with small to moderate improvements in chronic pain and function for certain chronic pain conditions. Nonopioid drug class–specific adverse events include serious cardiovascular, gastrointestinal, or renal effects with NSAIDs and sedation with anticonvulsants. Opioid therapy is associated with similar or decreased effectiveness for pain and function versus NSAIDs across several acute pain conditions and with small improvements in short-term (1 to <6 months) pain and function compared with placebo; evidence was found of attenuated pain reduction over time with opioids (between 3 and 6 months versus between 1 and 3 months). Opioid therapy is associated with increased risk for serious harms (including opioid use disorder and overdose) that appears to increase with increase in opioid dosage, without a clear threshold below which there is no risk. No validated, reliable way exists to predict which patients will suffer serious harm from opioid therapy. Evidence was sparse for long-term improvement of pain or function for any treatment for chronic pain. Some evidence indicated that beneficial effects of some nonpharmacologic therapies persist for up to 12 months after the end of a course of a treatment. Among 154 trials of nonopioid medications rated as good or fair quality, eight were long term (≥1 year). A single trial evaluated outcomes at 1 year for opioid medications (compared with nonopioid medications).

CDC invited input on the draft clinical practice guideline and received approximately 5,500 public comments. Many of these comments were related to experiences with pain or with the aftermath of a family member’s, friend’s, or significant person’s overdose; barriers to and access to pain care and evidence-based treatment; concerns about the level of specificity of recommendations; and overall communication and implementation of the clinical practice guideline. Some respondents expressed concerns that insufficient specificity of recommendations might leave clinicians without sufficient practical advice or context, whereas others were concerned that inclusion of more-specific recommendations or information in the guideline could facilitate misapplication through adaption of the clinical practice guideline or components of the guideline into rigid policies and laws. CDC incorporated insights from public comments into the clinical practice guideline, including special considerations for each recommendation. To help prevent misapplication of recommendations as inflexible rules and enable clinicians to account for individualized, person-centered clinical considerations, specific prescription dosages and durations are generally not included in the summary recommendation statements, which highlight general principles. Greater specificity is provided in implementation considerations and supporting rationales, which can offer more flexibility to help clinicians weigh benefits and risks of different therapeutic courses for specific patients.

Recommendation statements emphasize that opioids should be used only when benefits for pain and function are expected to outweigh risks. Before initiating opioid therapy for patients with pain, clinicians should discuss with patients the realistic benefits and known risks of opioid therapy. Before starting ongoing opioid therapy for patients with subacute or chronic pain, clinicians should work with patients to establish treatment goals for pain and function and consider how opioid therapy will be discontinued if benefits do not outweigh risks. When opioids are initiated, clinicians should prescribe the lowest effective dosage of immediate-release opioids for no longer than needed for the expected duration of pain severe enough to require opioids. During ongoing opioid therapy, clinicians should collaborate with patients to evaluate and carefully weigh benefits and risks of continuing opioid therapy and exercise care when increasing, continuing, or reducing opioid dosage. Before starting and periodically during continuation of opioid therapy, clinicians should evaluate risk for opioid-related harms and should work with patients to incorporate relevant strategies to mitigate risk, including offering naloxone and reviewing potential interactions with any other prescribed medications or substances used. Clinicians should offer or arrange treatment with evidence-based medications to treat patients with opioid use disorder.

CDC recommends that persons with pain receive appropriate pain treatment with careful consideration of the benefits and risks of all treatment options in the context of the patient’s circumstances. Clinicians should collaborate with patients when making treatment decisions and designing a treatment plan, including when initiating or changing pain management strategies and particularly when considering initiating, increasing, tapering, or discontinuing opioids. Clinicians should avoid abrupt discontinuation of opioids, especially for patients receiving high dosages of opioids, should avoid dismissing patients from care, and should ensure (provide or arrange) appropriate care for patients with pain and patients with complications from opioid use (e.g., opioid use disorder). Quality and equitable care across sociodemographic groups requires attention to mitigation of potential barriers to care, such as through linguistically tailored care and cost-assistance programs to ensure access to appropriate pharmacotherapy, psychological support, and physical therapy as needed.

This voluntary clinical practice guideline provides recommendations only and is intended to support, not supplant, clinical judgment and individualized, person-centered decision-making. This clinical practice guideline should not be applied as inflexible standards of care across patient populations by health care professionals; health systems; pharmacies; third-party payers; or state, local, or federal organizations or entities. This clinical practice guideline is intended to improve communication between clinicians and patients about the benefits and risks of pain treatment, including opioid therapy for pain; improve the safety and effectiveness of pain treatment; mitigate pain; improve function and quality of life for patients with pain; and reduce risks associated with opioid pain therapy, including opioid use disorder, overdose, and death.

BOX 2 **Intended use of CDC’s Clinical Practice Guideline for Prescribing Opioids for Pain — United States, 2022**

This clinical practice guideline is

- a clinical tool to improve communication between clinicians and patients and empower them to make informed, person-centered decisions related to pain care together;
- intended for primary care clinicians and other clinicians providing pain care for outpatients aged ≥18 years with
  - acute pain (duration of <1 month),
  - subacute pain (duration of 1–3 months), or
  - chronic pain (duration of >3 months); and
- intended to be flexible to enable person-centered decision-making, taking into account a patient’s expected health outcomes and well-being.

This clinical practice guideline is not

- a replacement for clinical judgment or individualized, person-centered care;
- intended to be applied as inflexible standards of care across patients or patient populations by health care professionals, health systems, pharmacies, third-party payers, or governmental jurisdictions or to lead to the rapid tapering or abrupt discontinuation of opioids for patients;
- a law, regulation, or policy that dictates clinical practice or as a substitute for Food and Drug Administration–approved labeling;
- applicable to
  - management of pain related to sickle cell disease,
  - management of cancer-related pain, or
  - palliative care or end-of-life care; or
- focused on opioids prescribed for opioid use disorder.

CDC developed a draft clinical practice guideline on the basis of five systematic reviews of the best-available evidence on the benefits and risks of prescription opioids, nonopioid pharmacologic treatments, and nonpharmacologic treatments. The draft clinical practice guideline was reviewed by an independent federal advisory committee (the Board of Scientific Counselors of the National Center for Injury Prevention and Control), peer reviewers, and the public and was revised after feedback from these reviews. Additional insights from patients, caregivers, and clinicians shared during virtual conversations held in 2020 were incorporated in the update. Importantly, to discourage the misapplication of opioid pain medication dosage thresholds as inflexible standards, revised recommendation statement language emphasizes principles such as avoiding increasing dosage above levels likely to yield diminishing returns in benefits relative to risks to patients. More-specific considerations related to dosage have been moved to implementation considerations that follow each recommendation statement, where more nuance is offered to inform clinical decision-making and individualized patient care.

This clinical practice guideline provides recommendations but does not replace clinical judgment and individualized, patient-centered decision-making. The recommendations are based on emerging evidence, including observational studies or randomized clinical trials with notable limitations; thus, they should be considered in the context of the clinician-patient relationship built on shared understanding and a whole-person approach that considers such factors as the patient’s physical and psychological functioning, support needs, expected health outcomes and well-being, home environment, and home and work responsibilities. Flexibility for clinicians and patients is paramount when making patient-centered clinical treatment decisions. The recommendations aim to improve communication between clinicians and patients about the benefits and risks of prescription opioids and other pain treatment strategies; improve the safety and effectiveness of pain treatment; improve pain, function, and quality of life for persons with pain; and reduce the risks associated with opioid pain treatment (including opioid use disorder, overdose, and death) and with other pain treatment.

This clinical practice guideline provides voluntary clinical practice recommendations for clinicians that should not be used as inflexible standards of care. The recommendations are not intended to be implemented as absolute limits for policy or practice across populations by organizations, health care systems, or government entities.

### Scope and Audience

This clinical practice guideline is intended for clinicians who are treating outpatients aged ≥18 years with acute (duration of <1 month), subacute (duration of 1–3 months), or chronic (duration of >3 months) pain, and excludes pain management related to sickle cell disease, cancer-related pain treatment, palliative care, and end-of-life care. The recommendations are most relevant to clinicians whose scope of practice includes prescribing opioids (e.g., physicians, nurse practitioners and other advanced-practice registered nurses, physician assistants, and oral health practitioners). Because clinicians might work within team-based care, this clinical practice guideline also refers to and promotes integrated pain management and collaborative working relationships among clinicians (e.g., behavioral health specialists such as social workers or psychologists, pharmacists, and registered nurses). This guideline update includes recommendations for primary care clinicians (e.g., internists and family physicians) and other clinicians managing pain in outpatient settings (e.g., surgeons, emergency medicine clinicians, occupational medicine clinicians, physical medicine and rehabilitation clinicians, and neurologists). Applicable settings include clinician offices, clinics, and urgent care centers. The recommendations do not apply to care provided to patients who are hospitalized or in an emergency department or other observational setting from which they might be admitted to inpatient care. These recommendations do apply to prescribing for pain management for patients when they are discharged from hospitals, emergency departments, or other facilities.

In addition to updating recommendations on the basis of new evidence regarding management of chronic pain, this clinical practice guideline is intended to assist clinicians in weighing benefits and risks of prescribing opioid pain medication for painful acute conditions (e.g., low back pain, neck pain, other musculoskeletal pain, neuropathic pain, dental pain, kidney stone pain, and acute episodic migraine) and pain related to procedures (e.g., postoperative pain and pain from oral surgery). In 2020, several of these indications were prioritized by an ad hoc committee of the National Academies of Sciences, Engineering, and Medicine (*86*) as those for which evidence-based clinical practice guidelines would help inform prescribing practices, with the greatest potential effect on public health. This update includes content on management of subacute painful conditions, when duration falls between that typically considered acute (defined as lasting <1 month) and chronic (defined as lasting >3 months). The durations used to define acute, subacute, and chronic pain might imply more specificity than is found in real-life patient experience, when pain often gradually transitions from acute to chronic. These time-bound definitions are not meant to be absolute but rather to be approximate guides to facilitate the consideration and practical use of the recommendations by clinicians and patients.

The 2016 CDC Opioid Prescribing Guideline focused on recommendations for primary care physicians. This clinical practice guideline expands the scope to additional clinicians. Although primary care physicians prescribe approximately 37% of all opioid prescriptions, other clinicians, including pain medicine clinicians (8.9%) and dentists (8.6%), account for considerable proportions of prescriptions. Pain medicine and physical medicine and rehabilitation clinicians prescribe opioids at the highest rates, followed by orthopedic and family medicine clinicians (*83*). Thus, expanding the scope to outpatient opioid prescribing can provide evidence-based advice for many additional clinicians, including dentists and other oral health providers, clinicians managing postoperative pain in outpatients, and clinicians providing pain management for patients being discharged from emergency departments.

Many principles of pain management are similar whether or not the treating clinician is a pain management specialist, and many of the recommendations might be relevant for pain management specialists. Many pain management specialists already follow principles outlined in this clinical practice guideline; however, use by pain management specialists is not the focus of this clinical practice guideline. Pain management specialists often have extensive training and expertise in pain management modalities that other clinicians do not, and they might treat patients with clinical situations that are more complex, less prevalent, and not well addressed by the available evidence; therefore, the balance of benefits and risks to patients might differ when the treating clinician is a pain management specialist.

The recommendations address the use of opioid pain medication in certain special populations (e.g., older adults and pregnant persons) and in populations with conditions posing special risks (e.g., a history of a substance use disorder). The recommendations do not address the use of opioid pain medication in children or adolescents aged <18 years. The available evidence concerning the benefits and risks of long-term opioid therapy in children and adolescents remains limited, and few opioid medications provide information in their labeling regarding safety and effectiveness in pediatric patients. Guidelines and recommendations are available for pain management in children with sickle cell disease (*87*), for children undergoing surgical procedures (*88*), and for palliative care in adolescent and young adult patients with cancer (*89*).

Although some principles in this clinical practice guideline might be helpful in the management of pain related to sickle cell disease, cancer-related pain treatment, palliative care, and end-of-life care, some recommendations might not be relevant for pain management in these contexts. Other guidelines more specifically address pain management in these situations (*87*,*89*–*93*); therefore, this clinical practice guideline does not apply to patients experiencing pain associated with these conditions or types of care. This does not imply that any other types of pain are more or less worthy of effective treatment, only that clinicians are referred to existing clinical guidelines that more specifically address unique considerations for management of pain related to sickle cell disease, cancer-related pain treatment, palliative care, and end-of-life care.

This clinical practice guideline follows the Institute of Medicine’s definition of palliative care as care that provides relief from pain and other symptoms, supports quality of life, and is focused on patients with serious advanced illness (*94*). Palliative care can begin early in the course of treatment for any serious illness that requires advanced management of pain or other distressing symptoms (*94*). In this guideline, end-of-life care refers to care for persons in hospice care and others with a terminal illness or at high risk for dying in the near future in hospitals, receiving long-term services and supports (including institutional care and home- and community-based services), or at home. This clinical practice guideline does not apply to patients undergoing cancer-related pain treatment, palliative care, or end-of-life care because of the unique therapeutic goals, ethical considerations, opportunities for medical supervision, and balance of benefits and risks with opioid therapy in such care. For example, for many persons at the end of life, serious potential long-term opioid-related harms such as opioid use disorder might not be relevant.

Recommendations on pain management for patients with cancer and patients who have survived cancer are available in the National Comprehensive Cancer Network (NCCN) Clinical Practice Guidelines in Oncology: Adult Cancer Pain (*90*), NCCN Clinical Practice Guidelines in Oncology: Survivorship (*91*), and Management of Chronic Pain in Survivors of Adult Cancers: American Society of Clinical Oncology (ASCO) Clinical Practice Guideline (*92*). Because of unique considerations in management of pain related to sickle cell disease, which can change the balance of benefits and risks of the use of opioids, clinicians should refer to the American Society of Hematology (ASH) 2020 Guidelines for Sickle Cell Disease: Management of Acute and Chronic Pain (*87*). In 2018, NCCN and ASCO convened and led a meeting including representatives and guideline authors from NCNN, ASCO, ASH, and CDC to review existing pain management guidelines and guidelines then in development from these organizations (*56*,*87*,*90*–*92*). Meeting participants noted that these guidelines applied to different patient populations and target audiences but found no disagreement among recommendations when applied to the appropriate patient and clinical situation (*95*).

Although this update includes content on pain management for patients with opioid use disorder and one recommendation on management of opioid use disorder as a complication of opioid use, recommendations on opioids used specifically as medications for opioid use disorder are not the focus of this clinical practice guideline. More detailed recommendations on management of patients with opioid use disorder are available in the American Society of Addiction Medicine (ASAM) National Practice Guideline for the Treatment of Opioid Use Disorder: 2020 Focused Update (*96*).

## Clinical Practice Guideline Development Methods

### Systematic Reviews and Evidence Sources

The 2016 CDC Opioid Prescribing Guideline was based on a systematic clinical evidence review sponsored by AHRQ on the effectiveness and risks of long-term opioid therapy for chronic pain (*47*,*97*), a CDC update to the AHRQ-sponsored review, and additional contextual questions (*56**,**98*). The systematic review addressed the effectiveness of long-term opioid therapy for outcomes related to pain, function, and quality of life; the comparative effectiveness of different methods for initiating and titrating opioids; the harms and adverse events associated with opioids; and the accuracy of risk prediction instruments and effectiveness of risk mitigation strategies on outcomes related to overdose, opioid use disorder, illicit drug use, and prescription opioid misuse. The CDC update to the AHRQ-sponsored review included literature published during or after 2015 and an additional question on the association between opioid therapy for acute pain and long-term use. The contextual evidence review addressed effectiveness of nonpharmacologic and nonopioid pharmacologic treatments, clinician and patient values and preferences, and information about resource allocation.

For this update to the 2016 CDC Opioid Prescribing Guideline, CDC funded AHRQ in 2018 and 2019 to conduct five systematic reviews (*7*–*11*). AHRQ’s Evidence-based Practice Centers completed these reviews, which included new evidence related to the treatment of chronic and acute pain. The AHRQ review of opioids for chronic pain updated and expanded the evidence for the 2016 CDC review; studies were included on short-term (1 to <6 months), intermediate-term (6 to <12 months) and long-term (≥12 months) outcomes of therapy involving opioids, effects of opioid plus nonopioid combination therapy, effects of tramadol, effects of naloxone coprescription, risks of coprescribed benzodiazepines, risks of coprescribed gabapentinoids, and effects of concurrent use of cannabis (*7*). The systematic clinical evidence review on opioids for chronic pain (*7*) also included contextual questions on clinician and patient values and preferences, costs and cost-effectiveness of opioid therapy, and risk mitigation strategies. CDC considered four new complementary AHRQ reviews on the benefits and harms of nonpharmacologic treatments for chronic pain (*9*), nonopioid pharmacologic treatments for chronic pain (*8*), treatments for acute episodic migraine (*11*), and treatments for acute (nonmigraine) pain (*10*). A question on management of acute pain in the systematic clinical evidence review for the 2016 CDC Opioid Prescribing Guideline was included in the new review on therapies for acute pain (*10*). CDC also reviewed AHRQ-sponsored surveillance reports conducted in follow-up to the five systematic reviews for any new evidence that could potentially change systematic review conclusions. To supplement the clinical evidence reviews, CDC sponsored a contextual evidence review on clinician and patient values and preferences and resource allocation (costs) for the areas addressed in the four new reviews (*8*–*11*).

### AHRQ Method for Evaluating Quality of Evidence

The reviews used the AHRQ approach to synthesize and grade the strength of evidence (*99*). The AHRQ approach is based on a systematic review of the evidence and provides an overall strength of evidence indicating the level of certainty (high, moderate, low, or insufficient); similar factors are considered in the Advisory Committee on Immunization Practices (ACIP) adapted (*100*,*101*) Grading of Recommendations Assessment, Development, and Evaluation (GRADE) (*102*) method. These factors include study limitations and risk for bias, consistency, directness, precision, and reporting bias. Large strength of association, dose response, and plausible confounders can strengthen observed findings. The primary clinical questions, detailed methods, and findings for the systematic and contextual evidence reviews are presented (Appendix).

### ACIP Adapted GRADE Method for Evaluating Quality of Evidence

The GRADE method is predicated on a systematic review of scientific evidence and provides a transparent framework for grading the quality of evidence and strength of recommendations. GRADE has been adapted by ACIP (*100*,*101*), and CDC used the ACIP adaptation in this clinical practice guideline. Under the ACIP GRADE framework, each body of evidence is initially categorized using a hierarchy that reflects the degree of confidence in the effect of a clinical action on health outcomes. The categories in the hierarchy are type 1 evidence (randomized clinical trials or overwhelming evidence from observational studies), type 2 evidence (randomized clinical trials with important limitations, or exceptionally strong evidence from observational studies), type 3 evidence (observational studies or randomized clinical trials with notable limitations), and type 4 evidence (clinical experience and observations, observational studies with important limitations, or randomized clinical trials with several major limitations) (Box 3). The evidence is downgraded if issues are identified with regard to risk for bias, inconsistency, indirectness, imprecision, or publication bias. Observational studies might be upgraded in certain situations (large strength of association, presence of dose response, or plausible effects of confounding would strengthen findings; that is, if confounding would likely provide results opposite to the observed findings, it strengthens the confidence that the observed association is present). A final evidence type is assigned based on these considerations. Type 1 evidence indicates high confidence that the true effect is close to the estimate of the effect; type 2 evidence means that the true effect is likely to be close to the estimate of the effect, but there is some uncertainty; type 3 evidence means that confidence in the effect estimate is limited (moderate uncertainty), and the true effect could differ substantially from the estimate of the effect; and type 4 evidence indicates very little confidence in the effect estimate (high uncertainty), and the likelihood is high that the true effect differs from the estimate of the effect (*100*,*103*). When no studies are available or the evidence is too limited to estimate effects, evidence is considered insufficient.

BOX 3 **Recommendations for prescribing opioids for outpatients with pain, excluding pain management related to sickle cell disease, cancer-related pain treatment, palliative care, and end-of-life care; recommendation categories; and evidence types — CDC Clinical Practice Guideline for Prescribing Opioids for Pain — United States, 2022**

**Determining Whether or Not to Initiate Opioids for Pain (Recommendations 1 and 2)**

1. Nonopioid therapies are at least as effective as opioids for many common types of acute pain. Clinicians should maximize use of nonpharmacologic and nonopioid pharmacologic therapies as appropriate for the specific condition and patient and only consider opioid therapy for acute pain if benefits are anticipated to outweigh risks to the patient. Before prescribing opioid therapy for acute pain, clinicians should discuss with patients the realistic benefits and known risks of opioid therapy (recommendation category: B; evidence type: 3).
2. Nonopioid therapies are preferred for subacute and chronic pain. Clinicians should maximize use of nonpharmacologic and nonopioid pharmacologic therapies as appropriate for the specific condition and patient and only consider initiating opioid therapy if expected benefits for pain and function are anticipated to outweigh risks to the patient. Before starting opioid therapy for subacute or chronic pain, clinicians should discuss with patients the realistic benefits and known risks of opioid therapy, should work with patients to establish treatment goals for pain and function, and should consider how opioid therapy will be discontinued if benefits do not outweigh risks (recommendation category: A; evidence type: 2).

**Selecting Opioids and Determining Opioid Dosages (Recommendations 3, 4, and 5)**

1. When starting opioid therapy for acute, subacute, or chronic pain, clinicians should prescribe immediate-release opioids instead of extended-release and long-acting (ER/LA) opioids (recommendation category: A; evidence type: 4).
2. When opioids are initiated for opioid-naïve patients with acute, subacute, or chronic pain, clinicians should prescribe the lowest effective dosage. If opioids are continued for subacute or chronic pain, clinicians should use caution when prescribing opioids at any dosage, should carefully evaluate individual benefits and risks when considering increasing dosage, and should avoid increasing dosage above levels likely to yield diminishing returns in benefits relative to risks to patients (recommendation category: A; evidence type: 3).
3. For patients already receiving opioid therapy, clinicians should carefully weigh benefits and risks and exercise care when changing opioid dosage. If benefits outweigh risks of continued opioid therapy, clinicians should work closely with patients to optimize nonopioid therapies while continuing opioid therapy. If benefits do not outweigh risks of continued opioid therapy, clinicians should optimize other therapies and work closely with patients to gradually taper to lower dosages or, if warranted based on the individual circumstances of the patient, appropriately taper and discontinue opioids. Unless there are indications of a life-threatening issue such as warning signs of impending overdose (e.g., confusion, sedation, or slurred speech), opioid therapy should not be discontinued abruptly, and clinicians should not rapidly reduce opioid dosages from higher dosages (recommendation category: B; evidence type: 4).

**Deciding Duration of Initial Opioid Prescription and Conducting Follow-Up (Recommendations 6 and 7)**

1. When opioids are needed for acute pain, clinicians should prescribe no greater quantity than needed for the expected duration of pain severe enough to require opioids (recommendation category: A; evidence type: 4).
2. Clinicians should evaluate benefits and risks with patients within 1–4 weeks of starting opioid therapy for subacute or chronic pain or of dosage escalation. Clinicians should regularly reevaluate benefits and risks of continued opioid therapy with patients (recommendation category: A; evidence type: 4).

**Assessing Risk and Addressing Potential Harms of Opioid Use (Recommendations 8, 9, 10, 11, and 12)**

1. Before starting and periodically during continuation of opioid therapy, clinicians should evaluate risk for opioid-related harms and discuss risk with patients. Clinicians should work with patients to incorporate into the management plan strategies to mitigate risk, including offering naloxone (recommendation category: A; evidence type: 4).
2. When prescribing initial opioid therapy for acute, subacute, or chronic pain, and periodically during opioid therapy for chronic pain, clinicians should review the patient’s history of controlled substance prescriptions using state prescription drug monitoring program (PDMP) data to determine whether the patient is receiving opioid dosages or combinations that put the patient at high risk for overdose (recommendation category: B; evidence type: 4).
3. When prescribing opioids for subacute or chronic pain, clinicians should consider the benefits and risks of toxicology testing to assess for prescribed medications as well as other prescribed and nonprescribed controlled substances (recommendation category: B; evidence type: 4).
4. Clinicians should use particular caution when prescribing opioid pain medication and benzodiazepines concurrently and consider whether benefits outweigh risks of concurrent prescribing of opioids and other central nervous system depressants (recommendation category: B; evidence type: 3).
5. Clinicians should offer or arrange treatment with evidence-based medications to treat patients with opioid use disorder. Detoxification on its own, without medications for opioid use disorder, is not recommended for opioid use disorder because of increased risks for resuming drug use, overdose, and overdose death (recommendation category: A; evidence type: 1).

**Recommendation categories** (on basis of evidence type, balance between desirable and undesirable effects, values and preferences, and resource allocation [cost]).

- **Category A recommendation**: Applies to all persons; most patients should receive the recommended course of action.
- **Category B recommendation**: Individual decision-making needed; different choices will be appropriate for different patients. Clinicians help patients arrive at a decision consistent with patient values and preferences and specific clinical situations.

**Evidence types** (on basis of study design and as a function of limitations in study design or implementation, imprecision of estimates, variability in findings, indirectness of evidence, publication bias, magnitude of treatment effects, dose-response gradient, and constellation of plausible biases that could change effects).

- **Type 1 evidence**: Randomized clinical trials or overwhelming evidence from observational studies.
- **Type 2 evidence**: Randomized clinical trials with important limitations, or exceptionally strong evidence from observational studies.
- **Type 3 evidence**: Observational studies or randomized clinical trials with notable limitations.
- **Type 4 evidence**: Clinical experience and observations, observational studies with important limitations, or randomized clinical trials with several major limitations.

### Categorizing the Evidence

The AHRQ approach uses a different method and terminology (high, moderate, low, or insufficient) to grade the strength of evidence from the ACIP adapted GRADE method (evidence types 1, 2, 3, or 4) (*99*). However, the underlying principles are similar, enabling translation from AHRQ to CDC grades. A methodologist translated the AHRQ strength of evidence grades to CDC evidence types according to the information provided in the summary of evidence tables in the AHRQ reviews. Tables with GRADE clinical evidence review ratings of the evidence for the key clinical questions are available (https://stacks.cdc.gov/view/cdc/121663). Evidence was categorized into the following types: type 1 (randomized clinical trials or overwhelming evidence from observational studies; equivalent to AHRQ high strength of evidence), type 2 (randomized clinical trials with important limitations, or exceptionally strong evidence from observational studies; equivalent to AHRQ moderate strength of evidence), type 3 (observational studies, or randomized clinical trials with notable limitations; equivalent to most AHRQ low strength of evidence ratings), or type 4 (clinical experience and observations, observational studies with important limitations, or randomized clinical trials with several major limitations; equivalent to AHRQ low strength of evidence with serious limitations). When no studies were available or the evidence was too limited to estimate effects, evidence was assessed as insufficient. Results from meta-analyses conducted for the AHRQ reviews were reported when available; otherwise, the evidence was synthesized qualitatively.

### Recommendation Development

CDC developed this clinical practice guideline using the method developed by the GRADE working group (https://www.gradeworkinggroup.org). Recommendations are based on the reviewed evidence. In the ACIP adapted GRADE framework, recommendations are assigned one of two categories (category A or B). Four major factors determine the category of the recommendation: 1) the quality of evidence, 2) the balance between desirable and undesirable effects, 3) values and preferences, and 4) resource allocation (e.g., costs to patients or health systems) (*104*). Other considerations include feasibility and acceptability and effect on equity (*105*). Recommendations are more likely to be category A when the evidence is higher quality, a balance of desirable relative to undesirable effects is greater, resources and costs are lower, and recommendations are less sensitive to differences in values and preferences. Category A recommendations typically apply to all persons in the group addressed in the recommendation and indicate a course of action that can be followed in most circumstances. Category B recommendations indicate that the recommendation might not apply to all persons in the group addressed in the recommendation; therefore, different choices will be appropriate for different patients, and decisions should be made based on the patient’s circumstances. For category B recommendations, clinicians must help patients arrive at a decision consistent with patient values and preferences and specific clinical situations (shared decision-making) (*106*). In the GRADE method, a particular quality of evidence does not necessarily result in a particular strength of recommendation (*102*–*104*). Although it is desirable for category A recommendations to be based on type 1 or type 2 evidence, category A recommendations can be based on type 3 or type 4 evidence when the advantages of a clinical action clearly outweigh the disadvantages in terms of benefits and harms, values and preferences, and costs, despite uncertainty in effect estimates (*104*). The GRADE working group has presented several paradigmatic situations in which strong (category A) recommendations might be justified despite low-quality evidence (e.g., when high-quality evidence suggests equivalence of two alternatives and low-quality evidence suggests harm in one alternative, or when high-quality evidence suggests modest benefits and low- or very low–quality evidence suggests possibility of catastrophic harm) (*104*). Category B recommendations are made when the advantages and disadvantages of a clinical action are more balanced or when more uncertainty exists with regard to whether benefits clearly outweigh harms.

In accordance with the ACIP adapted GRADE method, CDC drafted evidence-based recommendations focused on determining whether or not to initiate opioids for pain, selecting opioids and determining opioid dosages, deciding duration of initial opioid prescription and conducting follow-up, and assessing risk and addressing potential harms of opioid use. To help assure the draft guideline’s integrity and credibility, CDC then began a multistep review process.

### Federal Advisory Committee Review and Recommendation

CDC sought recommendations on the draft clinical practice guideline from one of its federal advisory committees, the Board of Scientific Counselors of the National Center for Injury Prevention and Control (BSC/NCIPC). BSC/NCIPC advises the U.S. Department of Health and Human Services (HHS) Secretary, the CDC Director, and the NCIPC Director and makes recommendations regarding scientific, programmatic, and research policies, strategies, objectives, projects, and priorities. BSC/NCIPC also reviews progress toward injury and violence prevention. BSC/NCIPC members are special government employees appointed by the HHS Secretary or their designee as CDC advisory committee members. Members are required to complete the Office of Government Ethics Form 450 annually to disclose relevant interests and report on their disclosures during meetings. Disclosures for BSC/NCIPC are reported in this clinical practice guideline. Meeting minutes and documents for public BSC/NCIPC meetings are available on the BSC/NCIPC website (https://www.cdc.gov/injury/bsc/meetings.html).

On December 4–5, 2019, CDC held a public meeting of BSC/NCIPC (announced via *Federal Register* 84 FR 57021; 84 FR 65159) and provided a presentation on the background for updating the clinical practice guideline. CDC then requested the formation of an Opioid Workgroup (OWG), under the parent BSC, whose primary purpose would be to review a draft clinical practice guideline and to develop a report of their observations for BSC/NCIPC (*107*). After considering CDC’s presentations, the proposed OWG Terms of Reference, and public comments, BSC/NCIPC voted unanimously to establish an OWG that reports to BSC/NCIPC. CDC then held a public nomination process for prospective OWG members (*107*).

To provide background to BSC/NCIPC for informing the creation of OWG with a balance of perspectives, CDC identified audiences that would be 1) directly affected by the clinical practice guideline, 2) directly involved with implementing or integrating recommendations into current practice, and 3) qualified to represent a specific discipline or expertise in alignment with the tasks of the workgroup for consideration by BSC/NCIPC. Identified groups with perspectives that would support the workgroup’s capacity included, but were not limited to, patients with pain, family members and caregivers, clinicians, public health practitioners, and research scientists. CDC announced the call for nominations at the December 4–5, 2019, public meeting and heard recommendations from the public during the public comment opportunities, as well as from BSC/NCIPC members, regarding recommendations for nominations. Persons interested in being considered for the workgroup were encouraged to submit self-nominations from December 4, 2019, through February 4, 2020. CDC’s BSC/NCIPC received 255 nominations for OWG.

After reviewing clinical expertise, professional credentials, and diversity in perspectives of all nominees (including diversity of gender, race and ethnicity, geographic region, institutional affiliations, and personal experiences relevant to pain management and providing care to patients with pain), OWG’s Designated Federal Officer (DFO) created a list of prospective workgroup members and sent them invitations to participate, along with conflict of interest disclosure forms. OWG’s DFO and BSC/NCIPC’s DFO reviewed conflict of interest disclosure forms. CDC’s Strategic Business Initiatives Unit (SBIU), which oversees the Federal Advisory Committee Act program, also reviewed the OWG Terms of Reference, prospective OWG roster, curricula vitae, and conflict of interest disclosure forms and determined all reported financial or other conflicts of interest were not present or nonsignificant before finalizing selection.* OWG members disclosed any potential topical conflicts of interest related to OWG meeting agenda items before each meeting. Disclosures of OWG are reported in the clinical practice guideline.

OWG had 23 members (*108*) including four ex officio members representing federal partner agencies (see Federal Partner Engagement). In accordance with CDC guidance (*109*,*110*) that at least two BSC/NCIPC members must serve on OWG and one of the two members must serve as the workgroup chair, OWG included a total of three BSC/NCIPC members, with one BSC/NCIPC member serving as the OWG chair. An NCIPC subject matter expert served as OWG’s DFO. OWG members included patients with pain, caregivers, and family members of patients with pain. OWG also comprised clinicians and subject matter experts, with the following perspectives represented: primary care, pain medicine, public health, behavioral health, pharmacy, emergency medicine, medical toxicology, obstetrics/gynecology, bioethics, orthopedic surgery, plastic surgery, dentistry, sickle cell disease, substance use disorder treatment, and research. OWG members were diverse in regard to gender, race and ethnicity, geographic region, institutional affiliation, subject matter expertise, and personal experiences. The CDC NCIPC OWG DFO presented the OWG roster and reviewed the Terms of Reference at the publicly held BSC/NCIPC meeting on July 22, 2020 (*Federal Register* 85 FR 30709; 85 FR 40290).

OWG had 11 meetings from October 2020 through June 2021. Before receiving the draft clinical practice guideline, OWG held meetings to review and discuss the 2016 CDC Opioid Prescribing Guideline; CDC’s community engagement activities with patients, caregivers, and clinicians; and GRADE methodology. CDC NCIPC staff provided OWG with evidence reviews, public comments from BSC/NCIPC meetings, and summaries of community engagements for review before providing OWG with the draft clinical practice guideline in March 2021. OWG held seven meetings to review and discuss the draft clinical practice guideline and develop a report summarizing their expert observations and findings for BSC/NCIPC. The OWG report provided overall observations on overarching themes and draft clinical practice guideline recommendations (*111*). In addition, many members of OWG developed a document entitled *OWG Guiding Principles* that was included as an appendix in the OWG report; this document outlines the “general process and principles by which OWG approached their assigned tasks.” These *Guiding Principles* included minimizing bias, ensuring scientific integrity, enhancing inclusivity, being patient and clinician centered, and considering historical context.

The OWG chair presented the OWG report at a public BSC/NCIPC meeting on July 16, 2021 (*Federal Register* 86 FR 30048). After hearing additional CDC presentations on the process and progress of the draft clinical practice guideline, discussion of the OWG report, and a 2-hour public comment period, BSC/NCIPC voted unanimously that CDC adopt the OWG report, while considering ideas and suggestions raised by BSC/NCIPC and the public during the meeting, and that OWG’s work be considered complete and that OWG be sunsetted. BSC/NCIPC provided their recommendations to HHS and CDC on July 20, 2021. CDC considered OWG’s observations, BSC/NCIPC recommendations, and public comments during BSC/NCIPC meetings when revising the draft clinical practice guideline (*112*,*113*). A list of BSC/NCIPC and of OWG members appears at the end of this report. The recommendations and all statements included in this guideline are those of CDC and do not necessarily represent the official position of any persons or organizations providing comments on this guideline.

### Federal Partner Engagement

BSC/NCIPC invited federal partners to serve as ex officio members of OWG, including representatives from the National Institute on Drug Abuse (NIDA) at the National Institutes of Health (NIH), the Substance Abuse and Mental Health Services Administration (SAMHSA), FDA, and the Indian Health Service (IHS). BSC/NCIPC included ex officio members from the Administration for Children and Families; the Administration on Aging in the Administration for Community Living; the National Institute for Occupational Safety and Health and the National Center for Health Statistics at CDC; the Health Resources and Services Administration; IHS; SAMHSA; and the National Institute on Aging, the National Institute of Child Health and Human Development, NIDA, and the National Institute of Mental Health at NIH. Additional federal partners were engaged throughout the clinical practice guideline update process. Federal partners reviewed the full draft clinical practice guideline as part of CDC’s agency clearance process.

### Public Comment and Community Engagement

CDC sought input through *Federal Register* notices to better understand community members’ experiences and perspectives related to pain and pain management options before drafting the clinical practice guideline (*113*). Through the *Federal Register* notice (85 FR 21441) posted from April 17, 2020, through June 16, 2020, CDC invited input specifically on topics focused on using or prescribing opioid pain medications, nonopioid medications, or nonpharmacologic treatments and received 5,392 public comments. Public comments were synthesized into common themes, using a CDC-funded analysis contract, and reviewed by CDC.

In addition, the Lab at the U.S. Office of Personnel Management (OPM) (https://lab.opm.gov) worked with CDC to design and implement community engagement opportunities. These opportunities were designed to gain additional insight into the values and preferences of groups including patients with acute or chronic pain, patients’ family members or caregivers, and clinicians who care for patients with pain or conditions that can complicate pain management (e.g., opioid use disorder or overdose).

CDC planned to have in-person individual conversations with patients, caregivers, and clinicians but pivoted to holding conversations with persons in a virtual format because of the COVID-19 pandemic. CDC posted a companion *Federal Register* notice (85 FR 44303) from July 22, 2020, through August 21, 2020, to solicit input from patients, caregivers, and clinicians interested in participating in individual conversations. After the *Federal Register* notice closed, CDC and OPM randomly selected participants within each group (i.e., patients, caregivers, and clinicians) from 973 respondents. CDC and OPM also developed a randomly selected waiting list of participants to fill conversation appointments that were missed or canceled by participants. The community engagement was authorized under the Generic Clearance for the Collection of Qualitative Feedback on Agency Service Delivery (OMB Control Number 0920–1050) approval for the Paperwork Reduction Act. CDC and OPM conducted telephone and video conversations throughout September 2020 and spoke with 106 persons, including 42 patients, 21 caregivers, and 43 clinicians. Participants lived and worked all over the United States and had diverse experiences with opioids. Participants provided verbal consent for their conversations to be recorded. A transcription service reviewed the conversation recordings to develop anonymized transcripts. CDC and OPM reviewed the anonymized transcripts to develop thematic summaries.

CDC and OPM also held two human-centered codesign workshops with staff from CDC and the Centers for Medicare & Medicaid Services. Workshop topics included framing priority needs for public input; objectives for individual conversations; and synthesizing engagement strategies on the basis of insights from public comments and conversations with patients, caregivers, and clinicians. Workshop participants included HHS staff who were themselves patients, caregivers, clinicians, clinical practice guideline authors, and other subject matter experts.

CDC also gathered input through oral and written public comment opportunities at and in conjunction with public BSC/NCIPC meetings. These public comment opportunities were announced through *Federal Register* notices (*Federal Register* 84 FR 57021; 84 FR 65159; 85 FR 30709; 85 FR 40290; 86 FR 1502; 86 FR 30048) and NCIPC newsletters.

CDC reviewed thematic summaries of public comments, individual conversations, and workshops to learn more about values and preferences of patients, caregivers, clinicians, and experts before drafting the clinical practice guideline (*113*). After incorporating observations and comments on the draft clinical practice guideline from BSC/NCIPC and the agency clearance process, CDC posted the revised full draft clinical practice guideline and supporting materials in the *Federal Register* for public comment (*Federal Register* 87 FR 7838). The public comment period was open for 60 days (February 10–April 11, 2022). The Federal Docket received approximately 5,500 unique comments (including one comment submitted with 28,322 additional signatories) from the public, including patients with acute and chronic pain, caregivers, and clinicians, and organizational perspectives from medical associations, professional organizations, academic institutions, state and local governments, and advocacy and industry groups. CDC reviewed and considered all public comments when revising the clinical practice guideline.

### Peer Review

This clinical practice guideline provides influential scientific information that could have a clear and substantial effect on public- and private-sector decisions. Therefore, peer review of the draft clinical practice guideline was required per the final information quality bulletin for peer review (https://www.whitehouse.gov/wp-content/uploads/2019/04/M-19-15.pdf).

CDC identified peer reviewers on the basis of multiple factors, including scientific and subject matter expertise, racial and ethnic diversity, gender diversity, diversity of experiences and perspectives, independence from the clinical practice guideline development process, and consideration of conflicts of interest. Specific effort was made to identify subject matter experts with knowledge and experience in topics such as chronic and acute pain management, clinical practice, health equity, mental health and well-being, opioids and opioid therapies, opioid tapering, opioid use disorder treatment, pharmacologic and nonpharmacologic pain management, and surgical pain management. CDC assessed potential conflicts of interest before finalizing selection of peer reviewers. The NCIPC Associate Director for Science reviewed conflict of interest disclosure forms and determined no conflicts of interest were present. After the peer reviews were completed, CDC posted the names of peer reviewers on the NCIPC and CDC/ATSDR Peer Review Agenda websites, which provide information about the peer review of influential government scientific documents (*114*,*115*). Peer reviewers independently reviewed the draft clinical practice guideline and evaluated its scientific merit and practical implementation considerations, with the goal of maintaining high-quality science and providing evidence-based recommendations to guide clinical practice and decision-making to help prevent opioid-related harms. CDC reviewed and considered peer review comments when revising the clinical practice guideline.

## Recommendations

This clinical practice guideline includes 12 recommendations for clinicians who are prescribing opioids for outpatients aged ≥18 years with acute (duration of <1 month), subacute (duration of 1–3 months), or chronic (duration of >3 months) pain, excluding pain management related to sickle cell disease, cancer-related pain treatment, palliative care, and end-of-life care (Box 3). The recommendations are not intended to be implemented as absolute limits of policy or practice across populations by organizations, health care systems, or government entities. In accordance with the ACIP adapted GRADE method, CDC based the recommendations on consideration of clinical evidence, contextual evidence (e.g., benefits and harms, values and preferences, and resource allocation), and expert opinion. Expert input is reflected within the recommendation rationales. For each recommendation statement, CDC notes the recommendation category (A or B) and the type of evidence (1, 2, 3, or 4) supporting the statement (Box 3).

Category A recommendations indicate that most patients should receive the recommended course of action; category B recommendations indicate that different choices will be appropriate for different patients, requiring clinicians to help patients arrive at a decision consistent with patient values and preferences and specific clinical situations. Consistent with the ACIP (*106*,*116*) and GRADE method (*103*), category A recommendations were made, even with type 3 and 4 evidence, when there was broad agreement that the advantages of a clinical action greatly outweighed the disadvantages. Category B recommendations were made when there was broad agreement that the advantages and disadvantages of a clinical action were more balanced, but advantages were significant enough to warrant a recommendation. Recommendations were associated with a range of evidence types, from type 1 to type 4.

In summary, the categorization of recommendations was based on the following assessment:

- A number of nonpharmacologic treatments and nonopioid medications are associated with improvements in pain, function, or both that are reportedly comparable to improvements associated with opioid use (*7*–*11*).
- Evidence exists that multiple noninvasive nonpharmacologic interventions improve chronic pain and function, with small to moderate effects in specific pain conditions, and are not associated with serious harms. Compared with medication treatment, for which benefits are anticipated while patients are taking the medication but are not usually expected to persist after patients stop taking the medication, multiple noninvasive nonpharmacologic interventions are associated with improvements in pain, function, or both that are sustained after completion of treatment (*9*).
- Nonopioid drugs, including serotonin and norepinephrine reuptake inhibitor (SNRI) antidepressants, pregabalin or gabapentin, and nonsteroidal anti-inflammatory drugs (NSAIDs), are associated with small to moderate improvements in chronic pain and function. Drug class–specific adverse events include serious cardiovascular, gastrointestinal, or renal effects with NSAIDs and sedation with anticonvulsants (*8*).
- Opioid therapy is associated with similar or decreased effectiveness for pain and function versus NSAIDs across multiple common acute pain conditions (*10*). Opioid therapy is associated with small improvements in short-term (duration of 1 to <6 months) pain and function compared with placebo, with increased short-term harms compared with placebo, and with evidence of attenuated pain reduction over time (between 3 and 6 months versus between 1 and 3 months) (*10*). Evidence exists from observational studies of an association between opioid use for acute pain and long-term opioid use (*10*). Evidence on long-term effectiveness of opioids remains very limited (*7*); a long-term (12 months) randomized trial of stepped therapy for chronic musculoskeletal pain found no difference in function and higher pain intensity after starting with opioid therapy compared with starting with nonopioid therapy (*74*). Evidence exists of increased risk for serious harms (including opioid use disorder and overdose) with long-term opioid therapy that appears to rise with increase in opioid dosage, without a clear threshold below which there is no risk (*7*).
- No validated, reliable way exists to predict which patients will experience serious harm from opioid therapy and which patients will benefit from opioid therapy (*7*).
- Discontinuing opioids after extended periods of continuous opioid use can be challenging for clinicians and patients. Tapering or discontinuing opioids in patients who have taken them long term can be associated with clinically significant risks (*68*), particularly if opioids are tapered rapidly or patients do not receive effective support.
- Patients, caregivers, and clinicians responded to CDC with invited input about their experiences and perspectives related to pain and pain management options. Themes included strained patient-clinician relationships and the need for patients and clinicians to make shared decisions, the effects of misapplication of the 2016 CDC Opioid Prescribing Guideline, inconsistent access to effective pain management solutions, and achieving reduced prescription opioid use through diverse approaches.
- Members of the public responded to CDC with invited comments. Themes included experiences with pain or experiences in the aftermath of the overdose of a friend, family member, or significant other; barriers and access to pain care and to evidence-based treatment; concerns about the level of specificity of recommendations; and overall communication and implementation of the clinical practice guideline.

Each of the 12 recommendation statements is followed by considerations for implementation and a rationale for the recommendation. The implementation considerations offer practical insights, context, and specific examples meant to further inform clinician-patient decision-making for the respective recommendation and are not meant to be rigidly or inflexibly followed.

The recommendations are grouped into four areas:

1. Determining whether or not to initiate opioids for pain
2. Selecting opioids and determining dosages
3. Deciding duration of initial opioid prescription and conducting follow-up
4. Assessing risk and addressing potential harms of opioid use

In addition, these five guiding principles should broadly inform implementation across recommendations (Box 4):

BOX 4 **Guiding principles for implementation of the CDC Clinical Practice Guideline for Prescribing Opioids for Pain — United States, 2022 recommendations**

1. Acute, subacute, and chronic pain needs to be appropriately assessed and treated independent of whether opioids are part of a treatment regimen.
2. Recommendations are voluntary and are intended to support, not supplant, individualized, person-centered care. Flexibility to meet the care needs and the clinical circumstances of a specific patient is paramount.
3. A multimodal and multidisciplinary approach to pain management attending to the physical health, behavioral health, long-term services and supports, and expected health outcomes and well-being of each person is critical.
4. Special attention should be given to avoid misapplying this clinical practice guideline beyond its intended use or implementing policies purportedly derived from it that might lead to unintended and potentially harmful consequences for patients.
5. Clinicians, practices, health systems, and payers should vigilantly attend to health inequities; provide culturally and linguistically appropriate communication, including communication that is accessible to persons with disabilities; and ensure access to an appropriate, affordable, diversified, coordinated, and effective nonpharmacologic and pharmacologic pain management regimen for all persons.

1. Acute, subacute, and chronic pain needs to be appropriately assessed and treated independent of whether opioids are part of a treatment regimen.
2. Recommendations are voluntary and are intended to support, not supplant, individualized, person-centered care. Flexibility to meet the care needs and the clinical circumstances of a specific patient is paramount.
3. A multimodal and multidisciplinary approach to pain management attending to the physical health, behavioral health, long-term services and supports, and expected health outcomes and well-being of each person is critical.
4. Special attention should be given to avoid misapplying this clinical practice guideline beyond its intended use or implementing policies purportedly derived from it that might lead to unintended and potentially harmful consequences for patients.
5. Clinicians, practices, health systems, and payers should vigilantly attend to health inequities; provide culturally and linguistically appropriate communication (*117*), including communication that is accessible to persons with disabilities; and ensure access to an appropriate, affordable, diversified, coordinated, and effective nonpharmacologic and pharmacologic pain management regimen for all persons.

### Determining Whether or Not to Initiate Opioids for Pain

All patients with pain should receive treatment that provides the greatest benefits relative to risks. (See Recommendation 1 for determining whether or not to initiate opioids for acute pain [i.e., pain lasting <1 month] and Recommendation 2 for determining whether or not to initiate opioids for subacute pain [i.e., pain lasting 1–3 months] or chronic pain [i.e., pain lasting >3 months].)

#### Recommendation 1

**Nonopioid therapies are at least as effective as opioids for many common types of acute pain. Clinicians should maximize use of nonpharmacologic and nonopioid pharmacologic therapies as appropriate for the specific condition and patient and only consider opioid therapy for acute pain if benefits are anticipated to outweigh risks to the patient. Before prescribing opioid therapy for acute pain, clinicians should discuss with patients the realistic benefits and known risks of opioid therapy (recommendation category: B; evidence type: 3).**

##### Implementation Considerations

- Nonopioid therapies are at least as effective as opioids for many common acute pain conditions, including low back pain, neck pain, pain related to other musculoskeletal injuries (e.g., sprains, strains, tendonitis, and bursitis), pain related to minor surgeries typically associated with minimal tissue injury and mild postoperative pain (e.g., simple dental extraction), dental pain, kidney stone pain, and headaches including episodic migraine.
- Clinicians should maximize use of nonopioid pharmacologic (e.g., topical or oral NSAIDs, acetaminophen) and nonpharmacologic (e.g., ice, heat, elevation, rest, immobilization, or exercise) therapies as appropriate for the specific condition.
- Opioid therapy has an important role for acute pain related to severe traumatic injuries (including crush injuries and burns), invasive surgeries typically associated with moderate to severe postoperative pain, and other severe acute pain when NSAIDs and other therapies are contraindicated or likely to be ineffective.
- When diagnosis and severity of acute pain warrant the use of opioids, clinicians should prescribe immediate-release opioids (see Recommendation 3) at the lowest effective dose (see Recommendation 4) and for no longer than the expected duration of pain severe enough to require opioids (see Recommendation 6).
- Clinicians should prescribe and advise opioid use only as needed (e.g., hydrocodone 5 mg/acetaminophen 325 mg, one tablet not more frequently than every 4 hours as needed for moderate to severe pain) rather than on a scheduled basis (e.g., one tablet every 4 hours) and encourage and recommend an opioid taper if opioids are taken around the clock for more than a few days (see Recommendation 6).
- If patients already receiving opioids long term require additional medication for acute pain, nonopioid medications should be used when possible and, if additional opioids are required (e.g., for superimposed severe acute pain), they should be continued only for the duration of pain severe enough to require additional opioids, returning to the patient’s baseline opioid dosage as soon as possible, including a taper to baseline dosage if additional opioids were used around the clock for more than a few days (see Recommendation 6).
- Clinicians should ensure that patients are aware of expected benefits of, common risks of, serious risks of, and alternatives to opioids before starting or continuing opioid therapy and should involve patients meaningfully in decisions about whether to start opioid therapy.

##### Supporting Rationale

Evaluation of the patient is critical to appropriate management. Evaluation can identify reversible causes of pain and underlying etiologies with potentially serious sequelae that require urgent action. To guide patient-specific selection of therapy, clinicians should evaluate patients and establish or confirm the diagnosis. Diagnosis can help identify interventions to reverse, ameliorate, or prevent worsening of pain and improve function (e.g., surgical intervention to repair structure and function after certain traumatic injuries, bracing to prevent recurrence of acute ankle sprain, fracture immobilization, ice or elevation to reduce swelling, and early mobilization to maintain function) (*118*).

#### Noninvasive Nonpharmacologic Approaches to Acute Pain

Noninvasive nonpharmacologic approaches to acute pain have the potential to improve pain and function without risk for serious harms (*10*). Clinical evidence reviews found that some nonpharmacologic treatments were likely effective for acute pain, such as heat therapy for acute low back pain; several others might be effective for specific acute pain conditions, such as spinal manipulation for acute back pain with radiculopathy, a cervical collar or exercise for acute neck pain with radiculopathy, acupressure for acute musculoskeletal pain, massage for postoperative pain (*10*), and remote electrical neuromodulation for acute pain related to episodic migraine (*11*).

The American College of Physicians (ACP) recommends nonpharmacologic treatment with superficial heat, massage, acupuncture, or spinal manipulation as a cornerstone of treatment for acute low back pain (*119*). ACP and the American Academy of Family Physicians (AAFP) suggest acupressure to improve pain and function and transcutaneous electrical nerve stimulation to reduce pain in patients with acute musculoskeletal injuries (*120*).

Despite evidence supporting their use, noninvasive nonpharmacologic therapies are not always or fully covered by insurance (*43*), and access and cost can be barriers, particularly for persons who are uninsured, have limited income, have transportation challenges, or live in rural areas where treatments are not available (*121*). Experts from OWG expressed concern about limited access to nonopioid pain management modalities, in part because of lack of availability or lack of coverage by payers, and emphasized improving access to nonopioid pain management modalities as a priority. Health insurers and health systems can contribute to improved pain management and reduced medication use by increasing access to noninvasive nonpharmacologic therapies with evidence of effectiveness (*9*,*43*). Noninvasive nonpharmacologic approaches should be used as appropriate to alleviate acute pain, including ice and elevation to reduce swelling and discomfort from musculoskeletal injuries, heat to alleviate low back pain, and other modalities depending on the cause of the acute pain.

#### Nonopioid Medications for Acute Pain

Many acute pain conditions often can be managed most effectively with nonopioid medications (*10*,*122*). A systematic review found that for musculoskeletal injuries such as sprains, whiplash, and muscle strains, topical NSAIDs provided the greatest benefit-harm ratio, followed by oral NSAIDs or acetaminophen with or without diclofenac (*122*). NSAIDs have been found to be more effective than opioids for surgical dental pain and kidney stone pain and similarly effective to opioids for low back pain (*10*). Evidence is limited on comparative effectiveness of therapies for acute neuropathic pain, neck pain, and postoperative pain (*10*). For episodic migraine, triptans, NSAIDs, antiemetics, dihydroergotamine, calcitonin gene-related peptide antagonists (gepants), and lasmiditan are associated with improved pain and function with usually mild and transient adverse events (*11*).

ACP recommends NSAIDs or skeletal muscle relaxants if pharmacologic treatment is desired to treat low back pain (*119*). For acute musculoskeletal injuries other than low back pain, ACP and AAFP recommend topical NSAIDs with or without menthol gel as first-line therapy and suggest oral NSAIDs to relieve pain or improve function or oral acetaminophen to reduce pain (*120*). The American Dental Association (ADA) recommends NSAIDs as first-line treatment for acute dental pain management (*123*). For acute kidney stone pain, NSAIDs are at least as effective as opioids (*124*–*127*), can decrease the ureteral smooth muscle tone and ureteral spasm (*128*) causing kidney stone pain, and are preferred for kidney stone pain if not contraindicated. Triptans, NSAIDs, combined triptans with NSAIDs, antiemetics, dihydroergotamine, and acetaminophen are established acute treatments for migraine (*11*). Lasmiditan, an 5-HT1F receptor agonist, and ubrogepant, a gepant, were approved by FDA in 2019 for the treatment of migraine (*129*); another gepant, rimegepant, was approved in 2020. Lasmiditan and the gepants were more effective than placebo in providing pain relief at 2 hours, 1 day, and 1 week (*11*). Adverse events related to these newer medications require further study; however, their mechanisms of action are believed to be nonvasoconstrictive (*130*) and potentially carry lower risks than vasoactive medications in patients with cardiovascular risk factors (*11*).

When not contraindicated, NSAIDs should be used for low back pain, painful musculoskeletal injuries (including minor pain related to fractures), dental pain, postoperative pain, and kidney stone pain; triptans, NSAIDs, or their combinations should be used along with antiemetics as needed for acute pain related to episodic migraine. NSAID use has been associated with serious gastrointestinal events and major coronary events (*8*), particularly in patients with cardiovascular or gastrointestinal comorbidities, and clinicians should weigh risks and benefits of use, dose, and duration of NSAIDs when treating older adults as well as patients with hypertension, renal insufficiency, heart failure, or those with risk for peptic ulcer disease or cardiovascular disease. Vasoactive effects of triptans and ergot alkaloids might preclude their use in patients with migraine who also have cardiovascular risk factors (*11*,*131*,*132*). Clinicians should review FDA-approved labeling, including boxed warnings, before initiating treatment with any pharmacologic therapy.

#### Pain Management for Pregnant and Postpartum Persons

For pain management in the postpartum period, the American College of Obstetricians and Gynecologists (ACOG) recommends stepwise, multimodal, shared decision-making, incorporating pharmacologic treatments that might include opioids. After vaginal delivery, ACOG recommends acetaminophen or NSAIDs, and if needed, adding an opioid. After cesarean delivery, ACOG recommends standard oral and parenteral medications such as acetaminophen, NSAIDs, or low-dose, low-potency, short-acting opioids with duration of opioid use limited to the shortest reasonable course expected for treating acute pain (*133*). ACOG recommends counseling persons who are prescribed opioids about the risk for central nervous system depression in the postpartum person and in the breastfed infant (*133*), noting that if a codeine-containing medication is selected, duration of therapy and neonatal signs of toxicity should be reviewed with patients and their families (*133*).

#### Opioid Medication for Acute Pain

A systematic review found that for musculoskeletal injuries such as sprains, whiplash, and muscle strains, no opioid provided better benefit than NSAIDs, and opioid use caused the most harms (*122*). The evidence review (*10*) found that opioids might not be more effective than nonopioid therapies for some acute pain conditions (*134*–*138*), and use of opioids might negatively affect recovery and function (*139*,*140*). The review found that opioids were probably less effective than NSAIDs for surgical dental pain and kidney stone pain, less effective than acetaminophen for kidney stone pain, and similarly effective as NSAIDs for low back pain (*10*). For postoperative pain, effects of opioids on pain intensity were inconsistent, and opioids were associated with increased likelihood of repeat or rescue analgesic use (*10*). Evidence was insufficient for opioids in treatment of episodic migraine (*11*). Compared with NSAIDs or acetaminophen, opioids were associated with increased risk for short-term adverse events, including any adverse event, nausea, dizziness, and somnolence (*10*). Observational studies found that opioid use for acute low back pain or postoperative pain was associated with increased likelihood of long-term opioid use (*10*). Proportions of adults with new long-term opioid use at follow-up after initiation for short-term use for postoperative pain have ranged from <1% to 13% (*141*–*146*). Odds of long-term opioid use at follow-up after initiation for short-term use for acute pain might be greater with higher dosage and longer initial duration of exposure. For example, one study found that, compared with no early opioid use for acute low back pain, the adjusted odds ratio was 2.08 (95% CI: 1.55–2.78) for an early prescription totaling 1–140 MME and increased to 6.14 (95% CI: 4.92–7.66) for an early prescription totaling ≥450 MME (*140*). In episodic migraine, opioids as well as butalbital-containing medications were associated with a twofold higher risk for development of medication overuse headache compared with simple analgesics and triptans (*11*,*147*). Serious adverse events were uncommon for opioids and other medications; however, studies were not designed to assess risk for overdose, opioid use disorder, or long-term harms (*10*).

For acute low back pain, ACP found insufficient evidence for effectiveness of opioids and recommends nonopioid medications (see Nonopioid Medications for Acute Pain) if choosing pharmacologic treatment (*119*). ACP and AAFP suggest against treating patients with acute pain from musculoskeletal injuries with opioids, including tramadol (*120*). ADA recommends NSAIDs as the first-line therapy for acute pain management (see Nonopioid Medications for Acute Pain) (*123*). Multiple guidelines that address prescribing for postoperative pain include both nonopioid and opioid treatment options and have emphasized multimodal analgesia, incorporating around-the-clock nonopioid analgesics and nonpharmacologic therapies and noting that systemic opioids often are needed postoperatively but are not required in all patients (*148*–*151*). The American Headache Society recommends against prescribing opioid or butalbital-containing medications as first-line treatment for recurrent headache disorders (*152*), and the American Academy of Neurology also recommends against use of both of these classes of medications for treatment of migraine, except as a last resort (*153*).

Because of equivalent or lesser effectiveness for pain relief compared with NSAIDs and risks for long-term opioid use after using opioids for acute pain, opioids are not recommended as first-line therapy for many common acute pain conditions, including low back pain, neck pain, pain related to other musculoskeletal injuries (e.g., sprains, strains, tendonitis, and bursitis), pain related to minor surgeries typically associated with minimal tissue injury and only mild postoperative pain (e.g., simple dental extraction), dental pain, kidney stone pain, and headaches including episodic migraine. Opioid therapy has an important role for acute pain related to severe traumatic injuries (including crush injuries and burns), invasive surgeries typically associated with moderate to severe postoperative pain, and other severe acute pain when NSAIDs and other therapies are contraindicated or likely to be ineffective.

When diagnosis and severity of acute pain warrant the use of opioids, clinicians should prescribe immediate-release opioids (see Recommendation 3) at the lowest effective dose (see Recommendation 4) and for no longer than the expected duration of pain severe enough to require opioids (see Recommendation 6) to minimize unintentional initiation of long-term opioid use. Clinicians should maximize use of nonopioid pharmacologic (e.g., NSAIDs, acetaminophen, or both) and nonpharmacologic (e.g., ice, heat, elevation, rest, immobilization, or exercise) therapies as appropriate for the specific condition and continue these therapies as needed after opioids are discontinued. Clinicians should work with patients to prevent prolonged opioid use, prescribe and advise opioid use only as needed (e.g., hydrocodone 5 mg/acetaminophen 325 mg, one tablet not more frequently than every 4 hours as needed for moderate to severe pain) rather than on a scheduled basis (e.g., one tablet every 4 hours), and encourage and include an opioid taper if opioids will be taken around the clock for more than a few days (see Recommendation 6). Clinicians should consider concurrent medical conditions, including sleep apnea, pregnancy, renal or hepatic insufficiency, mental health conditions, and substance use disorders, in assessing risks of opioid therapy (see Recommendation 8); offer naloxone, particularly if the patient or a household member has risk factors for opioid overdose (see Recommendation 8); use particular caution when prescribing benzodiazepines or other sedating medications with opioid pain medication (see Recommendation 11); and check the prescription drug monitoring program (PDMP) database to ensure a new opioid prescription will not contribute to cumulative opioid dosages or medication combinations that put the patient at risk for overdose (see Recommendation 9). If signs of opioid use disorder are present, clinicians should address concerns with the patient, offer or arrange medication treatment for patients who meet criteria for opioid use disorder, and use nonpharmacologic and pharmacologic treatments as appropriate to manage the patient’s pain (see Recommendation 12 and the ASAM National Practice Guideline for the Treatment of Opioid Use Disorder: 2020 Focused Update) (*96*).

Although findings regarding risks for new long-term opioid use after use for acute pain (*10*) relate specifically to patients who were previously opioid naïve, risks also might be associated with dosage escalation (see Recommendation 4) if patients already treated with long-term opioids are prescribed additional opioid medication for new acute pain superimposed on chronic pain. Therefore, strategies that minimize opioid use should be implemented for both opioid-naïve and opioid-tolerant patients with acute pain when possible. If patients receiving long-term opioid therapy require additional medication for acute pain, nonopioid medications should be used when possible. If additional opioids are required (e.g., for superimposed severe acute pain), they should be continued only for the duration of pain severe enough to require additional opioids, returning to the patient’s baseline opioid dosage as soon as possible, including an appropriate taper to baseline dosage if additional opioids were used around the clock for more than a few days (see Recommendation 6).

Patient education and discussion before starting outpatient opioid therapy are critical so that patient preferences and values can be understood and used to inform clinical decisions. Clinicians should ensure that patients are aware of expected benefits of, common risks of, serious risks of, and alternatives to opioids before starting or continuing opioid therapy and should involve patients in decisions about whether to start opioid therapy. Essential elements for communication and discussion with patients before prescribing outpatient opioid therapy for acute pain include the following:

- Advise patients that short-term opioid use can lead to unintended long-term opioid use and of the importance of working toward planned discontinuation of opioid use as soon as feasible, including a plan to appropriately taper opioids as pain resolves if opioids have been used around the clock for more than a few days (see Recommendation 6).
- Review communication mechanisms and protocols patients can use to tell clinicians of severe or uncontrolled pain and to arrange for timely reassessment and management.
- Advise patients about serious adverse effects of opioids, including potentially fatal respiratory depression and development of a potentially serious opioid use disorder (see Recommendation 12) that can cause distress and inability to fulfill major role obligations at work, school, or home.
- Advise patients about common effects of opioids, such as constipation, dry mouth, nausea, vomiting, drowsiness, confusion, tolerance, physical dependence, and withdrawal symptoms when stopping opioids. To prevent constipation associated with opioid use, advise patients to increase hydration and fiber intake and to maintain or increase physical activity as they are able. Prophylactic pharmacologic therapy (e.g., a stimulant laxative such as senna, with or without a stool softener) might be needed to ensure regular bowel movements if opioids are used for more than a few days. Stool softeners or fiber laxatives without another laxative should be avoided. To minimize withdrawal symptoms, clinicians should provide and discuss an opioid tapering plan when opioids will be used around the clock for more than a few days (see Recommendation 6). Limiting opioid use to the minimum needed to manage pain (e.g., taking the opioid only when needed if needed less frequently than every 4 hours and the prescription is written for every 4 hours as needed for pain) can help limit development of tolerance and therefore withdrawal after opioids are discontinued.
- If formulations are prescribed that combine opioids with acetaminophen, advise patients of the risks of taking additional over-the-counter products containing acetaminophen.
- To help patients assess when a dose of opioids is needed, explain that the goal is to reduce pain to make it manageable rather than to eliminate pain.
- Discuss effects that opioids might have on a person’s ability to safely operate a vehicle or other machinery, particularly when opioids are initiated or when other central nervous system depressants (e.g., benzodiazepines or alcohol) are used concurrently.
- Discuss the potential for workplace toxicology testing programs to detect therapeutic opioid use.
- Discuss increased risks for opioid use disorder, respiratory depression, and death at higher dosages, along with the importance of taking only the amount of opioids prescribed (i.e., not taking more opioids than prescribed or taking them more often).
- Review increased risks for respiratory depression when opioids are taken with benzodiazepines, other sedatives, alcohol, nonprescribed or illicit drugs (e.g., heroin), or other opioids (see Recommendations 8 and 11).
- Discuss risks to household members and other persons if opioids are intentionally or unintentionally shared with others for whom they are not prescribed, including the possibility that others might experience overdose at the same or lower dosage than prescribed for the patient and that young children and pets are susceptible to unintentional ingestion. Discuss storage of opioids in a secure and preferably locked location, options for safe disposal of unused opioids (*154*), and the value of having naloxone available.
- Discuss planned use of precautions to reduce risks, including naloxone for overdose reversal (see Recommendation 8) and clinician use of PDMP information (see Recommendation 9).

#### Recommendation 2

**Nonopioid therapies are preferred for subacute and chronic pain. Clinicians should maximize use of nonpharmacologic and nonopioid pharmacologic therapies as appropriate for the specific condition and patient and only consider initiating opioid therapy if expected benefits for pain and function are anticipated to outweigh risks to the patient. Before starting opioid therapy for subacute or chronic pain, clinicians should discuss with patients the realistic benefits and known risks of opioid therapy, should work with patients to establish treatment goals for pain and function, and should consider how opioid therapy will be discontinued if benefits do not outweigh risks (recommendation category: A; evidence type: 2).**

##### Implementation Considerations

- To guide patient-specific selection of therapy, clinicians should evaluate patients and establish or confirm the diagnosis.
- Clinicians should recommend appropriate noninvasive nonpharmacologic approaches to help manage chronic pain, such as exercise (e.g., aerobic, aquatic, or resistance exercises) or exercise therapy (a prominent modality in physical therapy) for back pain, fibromyalgia, and hip or knee osteoarthritis; weight loss for knee osteoarthritis; manual therapies for hip osteoarthritis; psychological therapy, spinal manipulation, low-level laser therapy, massage, mindfulness-based stress reduction, yoga, acupuncture, and multidisciplinary rehabilitation for low back pain; mind-body practices (e.g., yoga, tai chi, or qigong), massage, and acupuncture for neck pain; cognitive behavioral therapy, myofascial release massage, mindfulness practices, tai chi, qigong, acupuncture, and multidisciplinary rehabilitation for fibromyalgia; and spinal manipulation for tension headache.
- Low-cost options to integrate exercise include walking in public spaces or use of public recreation facilities for group exercise. Physical therapy can be helpful, particularly for patients who have limited access to safe public spaces or public recreation facilities for exercise or whose pain has not improved with low-intensity physical exercise.
- Health insurers and health systems can improve pain management and reduce medication use and associated risks by increasing reimbursement for and access to noninvasive nonpharmacologic therapies with evidence for effectiveness.
- Clinicians should review FDA-approved labeling, including boxed warnings, and weigh benefits and risks before initiating treatment with any pharmacologic therapy.
- When patients affected by osteoarthritis have an insufficient response to nonpharmacologic interventions such as exercise for arthritis pain, topical NSAIDs can be used in patients with pain in a single or few joints near the surface of the skin (e.g., knee). For patients with osteoarthritis pain in multiple joints or incompletely controlled with topical NSAIDs, duloxetine or systemic NSAIDs can be considered.
- NSAIDs should be used at the lowest effective dose and shortest duration needed and should be used with caution, particularly in older adults and in patients with cardiovascular comorbidities, chronic renal failure, or previous gastrointestinal bleeding.
- When patients with chronic low back pain have had an insufficient response to nonpharmacologic approaches such as exercise, clinicians can consider NSAIDs or duloxetine for patients without contraindications.
- Tricyclic, tetracyclic, and SNRI antidepressants; selected anticonvulsants (e.g., pregabalin, gabapentin enacarbil, oxcarbazepine); and capsaicin and lidocaine patches can be considered for neuropathic pain. In older adults, decisions to use tricyclic antidepressants should be made judiciously on a case-by-case basis because of risks for confusion and falls.
- Duloxetine and pregabalin are FDA-approved for the treatment of diabetic peripheral neuropathy, and pregabalin and gabapentin are FDA-approved for treatment of postherpetic neuralgia.
- In patients with fibromyalgia, tricyclic (e.g., amitriptyline) and SNRI antidepressants (e.g., duloxetine, milnacipran), NSAIDs (e.g., topical diclofenac), and specific anticonvulsants (i.e., pregabalin and gabapentin) are used to improve pain, function, and quality of life. Duloxetine, milnacipran, and pregabalin are FDA-approved for the treatment of fibromyalgia. In older adults, decisions to use tricyclic antidepressants should be made judiciously on a case-by-case basis because of risks for confusion and falls.
- Patients with co-occurring pain and depression might be especially likely to benefit from antidepressant medication (see Recommendation 8).
- Opioids should not be considered first-line or routine therapy for subacute or chronic pain. This does not mean that patients should be required to sequentially fail nonpharmacologic and nonopioid pharmacologic therapy or be required to use any specific treatment before proceeding to opioid therapy. Rather, expected benefits specific to the clinical context should be weighed against risks before initiating therapy. In some clinical contexts (e.g., serious illness in a patient with poor prognosis for return to previous level of function, contraindications to other therapies, and clinician and patient agreement that the overriding goal is patient comfort), opioids might be appropriate regardless of previous therapies used. In other situations (e.g., headache or fibromyalgia), expected benefits of initiating opioids are unlikely to outweigh risks regardless of previous nonpharmacologic and nonopioid pharmacologic therapies used.
- Opioid therapy should not be initiated without consideration by the clinician and patient of an exit strategy to be used if opioid therapy is unsuccessful.
- Before opioid therapy is initiated for subacute or chronic pain, clinicians should determine jointly with patients how functional benefit will be evaluated and establish specific, measurable treatment goals.
- For patients with subacute pain who started opioid therapy for acute pain and have been treated with opioid therapy for ≥30 days, clinicians should ensure that potentially reversible causes of chronic pain are addressed and that opioid prescribing for acute pain does not unintentionally become long-term opioid therapy simply because medications are continued without reassessment. Continuation of opioid therapy at this point might represent initiation of long-term opioid therapy, which should occur only as an intentional decision that benefits are likely to outweigh risks after informed discussion between the clinician and patient and as part of a comprehensive pain management approach.
- Clinicians seeing new patients already receiving opioids should establish treatment goals, including functional goals, for continued opioid therapy. Clinicians should avoid rapid tapering or abrupt discontinuation of opioids (see Recommendation 5).
- Patient education and discussion before starting opioid therapy are critical so that patient preferences and values can be understood and used to inform clinical decisions.
- Clinicians should review available low-cost options for pain management for all patients and particularly for patients who have low incomes, do not have health insurance, or have inadequate insurance.
- Clinicians should ensure that patients are aware of expected benefits of, common risks of, serious risks of, and alternatives to opioids before starting or continuing opioid therapy and should involve patients in decisions about whether to start opioid therapy.

##### Supporting Rationale

To guide patient-specific selection of therapy, clinicians should evaluate patients and establish or confirm the diagnosis (*155*). Detailed recommendations on diagnosis are provided in other guidelines (*156*–*159*). Evaluation should include a focused history, including history and characteristics of pain and potential contributing factors (e.g., function, work history and current work demands, psychosocial stressors, and sleep), and physical examination, with imaging or other diagnostic testing only if indicated (e.g., if severe or progressive neurologic deficits are present or if serious underlying conditions are suspected) (*158*,*159*). For complex pain syndromes, consultation with a pain specialist can be considered to assist with diagnosis and management.

Diagnosis can help identify disease-specific interventions to reverse, ameliorate, or prevent worsening of pain and improve function (e.g., improving glucose control to prevent progression of diabetic neuropathy; immune-modulating agents for rheumatoid arthritis; physical or occupational therapy to address posture, muscle weakness, or repetitive occupational motions that contribute to musculoskeletal pain; or surgical intervention to relieve severe mechanical or compressive pain) (*159*). The underlying mechanism for most pain syndromes has traditionally been categorized as neuropathic (e.g., diabetic neuropathy and postherpetic neuralgia) or nociceptive (e.g., osteoarthritis and muscular back pain). More recently, nociplastic pain has been suggested as a third, distinct category of pain with augmented central nervous system pain and sensory processing and altered pain modulation as experienced in conditions such as fibromyalgia (*160*). The diagnosis and pathophysiologic mechanism of pain have implications for symptomatic pain treatment with medication. For example, evidence is limited for improved pain or function, or evidence exists of worse outcomes, with long-term use of opioids for several chronic pain conditions for which opioids are commonly prescribed, such as osteoarthritis (*161*), nonspecific low back pain (*119*,*162*), headache (*152*), and fibromyalgia (*163*,*164*). For moderate to severe chronic back pain or hip or knee osteoarthritis pain, a nonopioid strategy starting with acetaminophen or NSAIDs results in improved pain intensity with fewer side effects compared with a strategy starting with opioids (*74*). Tricyclic antidepressants, SNRI antidepressants, selected anticonvulsants, or transdermal lidocaine are recommended for neuropathic pain syndromes (e.g., diabetic neuropathy or postherpetic neuralgia) (*156*).

Review of the patient’s history and context beyond the presenting pain syndrome is helpful in selection of pain treatments. In particular, medications should be used only after assessment and determination that expected benefits outweigh risks, considering patient-specific factors. For example, clinicians should consider fall risk when selecting and dosing potentially sedating medications (e.g., tricyclic antidepressants, anticonvulsants, and opioids) and should weigh benefits and risks of use, dosage, and duration of NSAIDs when treating older adults and patients with hypertension, renal insufficiency, heart failure, or those with risk for peptic ulcer disease or cardiovascular disease. NSAIDs are potentially inappropriate for use in older adults with chronic pain because of higher risk for adverse effects with prolonged use (*165*). Some guidelines recommend topical NSAIDs for localized osteoarthritis (e.g., knee osteoarthritis) over oral NSAIDs in patients aged ≥75 years to minimize systemic effects (*166*). (See Recommendation 8 for additional considerations for assessing risks of opioid therapy.)

#### Noninvasive Nonpharmacologic Approaches to Subacute and Chronic Pain

Many noninvasive nonpharmacologic approaches, including physical therapy, weight loss for knee osteoarthritis, and behavioral therapies (e.g., cognitive behavioral therapy and mindfulness-based stress reduction), can improve pain and function without risk for serious harms (*9*). High-quality evidence exists that exercise therapy (a prominent modality in physical therapy) for back pain, fibromyalgia, and hip or knee osteoarthritis reduces pain and improves function immediately after treatment and that the improvements are sustained for at least 2–6 months (*9*,*167*–*170*). Previous guidelines have recommended aerobic, aquatic, or resistance exercises for persons with chronic pain, including osteoarthritis of the knee or hip, back pain, and fibromyalgia (*119*,*156*,*166*,*171*). Other noninvasive nonpharmacologic therapies that improve pain, function, or both for at least 1 month after delivery without apparent risk for serious harm include cognitive behavioral therapy for knee osteoarthritis; manual therapies for hip osteoarthritis; psychological therapy, spinal manipulation, low-level laser therapy, massage, mindfulness-based stress reduction, yoga, acupuncture, and multidisciplinary rehabilitation for low back pain; mind-body practices (e.g., yoga, tai chi, and qigong), massage, and acupuncture for neck pain; cognitive behavioral therapy, myofascial release massage, mindfulness practices, tai chi, qigong, acupuncture, and multidisciplinary rehabilitation for fibromyalgia; and spinal manipulation for tension headache (*9*). For temporomandibular disorder pain, patient education and self-care can be effective, as can occlusal splints for some patients and biobehavioral therapy for prevention of disabling symptoms (*172*,*173*). Exercise, mind-body interventions, and behavioral treatments (including cognitive behavioral therapy and mindfulness practices) can encourage active patient participation in the care plan and help address the effects of pain in the patient’s life; these active therapies have somewhat more robust evidence for sustained improvements in pain and function than more passive treatments (e.g., massage), particularly at longer-term follow-up (*9*). In addition, physical activity can provide additional health benefits, such as preventing or reducing symptoms of depression (*174*). Active approaches that engage the patient should be used when possible, with a supplementary role for more passive approaches, to reduce pain and improve function.

Despite their favorable benefit-to-risk profile, noninvasive nonpharmacologic therapies are not always covered or fully covered by insurance (*43*). Access and cost can be barriers for patients, particularly persons who have low incomes, do not have health insurance or have inadequate insurance, have transportation challenges, or live in rural areas where services might not be available (*121*). Health insurers and health systems can improve pain management and reduce medication use and associated risks by increasing reimbursement for and access to noninvasive nonpharmacologic therapies with evidence for effectiveness (*9*,*43*). In addition, for many patients, aspects of these approaches can be used even when access to specialty care is limited. For example, previous guidelines have strongly recommended aerobic, aquatic, or resistance exercises for patients with osteoarthritis of the knee or hip (*166*) and maintenance of physical activity, including normal daily activities, for patients with low back pain (*158*). A randomized trial found no difference in reduced chronic low back pain intensity, frequency, or disability between patients assigned to relatively low-cost group aerobics and those assigned to individual physiotherapy or muscle reconditioning sessions (*175*). Low-cost options to integrate exercise include walking in public spaces or use of public recreation facilities for group exercise. Physical therapy can be helpful, particularly for patients who have limited access to safe public spaces or public recreation facilities for exercise or whose pain has not improved with low-intensity physical exercise. A randomized trial found a stepped exercise program, in which patients were initially offered an Internet-based exercise program and progressively advanced to biweekly coaching calls and then to in-person physical therapy if not improved at previous steps, successfully improved symptomatic knee osteoarthritis, with 35% of patients ultimately requiring in-person physical therapy (*176*). In addition, primary care clinicians can integrate elements of psychosocial therapies such as cognitive behavioral therapy, which addresses psychosocial contributors to pain and improves function (*177*), by encouraging patients to take an active role in the care plan, supporting patients in engaging in activities such as exercise that are typically beneficial but that might initially be associated with fear of exacerbating pain (*159*), or providing education in relaxation techniques and coping strategies. In many locations, free or low-cost patient support, self-help, and educational community-based or employer-sponsored programs are available that can provide stress reduction and other mental health benefits. Clinicians should become familiar with such options within their communities so they can refer patients to low-cost services. Patients with higher levels of anxiety or fear related to pain or other clinically significant psychological distress can be referred for treatment with a mental health specialist (e.g., psychologist, psychiatrist, or clinical social worker).

#### Nonopioid Medications for Subacute and Chronic Pain

Several nonopioid pharmacologic therapies (including acetaminophen, NSAIDs, and selected antidepressants and anticonvulsants) are used for painful symptoms in chronic pain conditions. Nonopioid pharmacologic therapies are associated with risks, particularly in older adults, pregnant patients, and patients with certain comorbidities such as cardiovascular, renal, gastrointestinal, and liver disease. For example, NSAID use has been associated with serious gastrointestinal events and major coronary events (*8*). Increases in nonserious adverse events have been found with anticonvulsants pregabalin (blurred vision, cognitive effects, sedation, weight gain, dizziness, and peripheral edema) and gabapentin (blurred vision, cognitive effects, sedation, and weight gain), cannabis (nausea and dizziness), and SNRI antidepressants duloxetine (nausea and sedation) and milnacipran (nausea); dosage reductions reduced the risk for some adverse events with SNRI antidepressants (*8*). Clinicians should review FDA-approved labeling, including boxed warnings, before initiating treatment with any pharmacologic therapy.

For osteoarthritis, NSAIDs including topical NSAIDs and SNRI antidepressant duloxetine have small to moderate benefits for pain and function at short-term assessment (3–6 months), with intermediate-term (6–12 months) evidence for certain medications (celecoxib and duloxetine) and evidence that duloxetine is more effective in older (>65 years) than younger patients and in patients with knee osteoarthritis (*8*). Acetaminophen has limited evidence for effectiveness (*8*) and is no longer considered a first-line treatment for osteoarthritis (*161*). When patients have an insufficient response to nonpharmacologic interventions (e.g., exercise for arthritis pain), and if a single or a few joints near the surface of the skin (e.g., knee) are affected by osteoarthritis, use of topical NSAIDs is recommended (*161*). In patients with osteoarthritis pain in multiple joints or incompletely controlled pain with topical NSAIDs, systemic NSAIDs or duloxetine can be used. However, systemic NSAIDs should be used at the lowest effective dosage and shortest duration needed because risks might increase with longer use and at higher dosages (*178*).

Oral NSAIDs should be used with caution, particularly in older persons and in patients with cardiovascular comorbidities, chronic renal failure, or previous gastrointestinal bleeding. In patients with gastrointestinal comorbidities but without current or previous gastrointestinal bleeding, cyclooxygenase-2 inhibitors or NSAIDs with proton pump inhibitors can be used to minimize risk compared with risk with use of NSAIDs alone (*161*).

Moderate-quality evidence demonstrates small improvements in chronic low back pain with NSAIDs (*119*) and with duloxetine (*8*). When patients with low back pain have had an insufficient response to nonpharmacologic approaches such as exercise, clinicians can consider NSAIDs or duloxetine (*119*) for patients without contraindications.

For temporomandibular disorder pain that is not sufficiently improved with nonpharmacologic interventions, NSAIDs can be effective (*179*,*180*). Tricyclic, tetracyclic, and SNRI antidepressants; selected anticonvulsants; and capsaicin and lidocaine patches are recommended for neuropathic pain (*156*). However, evidence on topical lidocaine and capsaicin is limited (*8*). SNRI antidepressant duloxetine and anticonvulsants pregabalin, gabapentin, enacarbil, and oxcarbazepine are associated with small improvements in neuropathic pain (mainly diabetic neuropathy and postherpetic neuralgia) (*8*). Duloxetine and pregabalin are FDA-approved for the treatment of diabetic neuropathy, and pregabalin and gabapentin are FDA-approved for treatment of postherpetic neuralgia.

In patients with fibromyalgia, multiple medications are associated with small to moderate improvements in pain, function, and quality of life, including SNRI antidepressants (duloxetine and milnacipran), NSAIDs (topical diclofenac), and specific anticonvulsants (pregabalin and gabapentin) (*8*). Tricyclic and SNRI antidepressants also can relieve fibromyalgia symptoms. Duloxetine, milnacipran, and pregabalin are FDA-approved for and are recommended for the treatment of fibromyalgia (*156*). Tricyclic antidepressant amitriptyline often is used and recommended for patients with fibromyalgia (*156*), although evidence for its effectiveness is limited (*8*). Because patients with chronic pain might experience concurrent depression (*181*) and depression can exacerbate physical symptoms including pain (*182*), patients with co-occurring pain and depression might be especially likely to benefit from antidepressant medication (see Recommendation 8).

Tricyclic antidepressants are potentially inappropriate for older adults (aged ≥65 years) because of their anticholinergic effects (*165*). Evidence on effectiveness of cannabis for painful conditions is limited and inconsistent across studies, and some studies have reported adverse events such as dizziness, nausea, and sedation (*8*,*183*).

#### Opioid Medication for Subacute and Chronic Pain

Clinical evidence reviews found insufficient evidence to determine long-term benefits of opioid therapy for chronic pain and found an increased risk for serious harms related to long-term opioid therapy that appears to be dose dependent (*7*). Compared with no opioid use, opioid use was associated with increased risk for opioid use disorder, overdose, all-cause deaths, fractures, falls, and myocardial infarction (*7*). Opioids also were associated with increased risk for discontinuation because of gastrointestinal adverse events, somnolence, dizziness, and pruritus (*7*). Compared with placebo, at short-term follow-up (1 to <6 months), opioids were associated with small mean improvements in pain intensity (mean difference: −0.79 on a 0–10 scale; 95% CI: −0.93 to −0.67; I^2^: 71%) and function (*7*). Some evidence indicates that improvement in pain is reduced with longer duration of opioid therapy, from a mean improvement of 1 on a 0–10 scale at 1–3 months to approximately 0.5 at 3–6 months (*7*). No placebo-controlled trial evaluated effectiveness of opioids at intermediate (6 to <12 months) or long-term (≥12 months) follow-up (*7*). Compared with nonopioid treatments at short-term follow-up, there were no differences in mean pain improvement (mean difference: −0.29 on a 0–10 scale; 95% CI: −0.61 to 0.03) or functional improvement. No trials were identified that compared opioids with nonopioid therapies at intermediate- or long-term follow-up, with the exception of one trial that found stepped therapy starting with opioids to be associated with higher pain intensity than stepped therapy starting with nonopioids (4.0 versus 3.5; mean difference: 0.5; 95% CI: 0–1.0) at 12 months (*7*,*74*).

Clinical evidence reviews identified an observational study (*54*) finding long-term (>90 days’ supply) opioid prescription to be associated with considerably increased risk for a new opioid use disorder diagnosis for all dosages of long-term (>90 days’ supply) opioids prescribed compared with no opioids prescribed, with adjusted odds ratios of 15, 29, and 122 at low (1–36 MME/day), medium (36–120 MME/day), and high (≥120 MME/day) opioid dosages, respectively. Compared with no opioid use, opioid use was associated with increased risk for opioid use disorder, overdose, all-cause deaths, fractures, falls, and myocardial infarction (*7*).

Multiple experts from OWG stated that they appreciated this recommendation because of the importance of highlighting both pain and function, sharing realistic expectations with patients before initiating treatment, and paying attention to tapering and exit strategies. Although some experts reasoned the recommendation statement could state nonopioid therapies “may be preferred” or “may be effective” for chronic pain, others agreed with language that nonopioid therapies “are preferred” for chronic pain because opioid therapies are associated with small short-term benefits compared with placebo, comparable or reduced short-term benefits compared with nonopioid therapies, uncertain long-term benefits, and potential for serious harms.

Opioids should not be considered first-line or routine therapy for subacute or chronic pain. Although evidence on long-term benefits of nonopioid therapies also is limited, these therapies also are associated with short-term benefits, no evidence exists for attenuated benefit over time or difficulty stopping therapy when benefits do not outweigh risks, and risks for serious harms are usually lower. This does not mean that patients should be required to sequentially fail nonpharmacologic and nonopioid pharmacologic therapy or be required to use any specific treatment before proceeding to opioid therapy. Rather, expected benefits specific to the clinical context should be weighed against risks before initiating therapy. In some clinical contexts (e.g., serious illness in a patient with poor prognosis for return to previous level of function, contraindications to other therapies, and clinician and patient agreement that the overriding goal is patient comfort), opioids might be appropriate regardless of previous therapies used. In other situations (e.g., headache or fibromyalgia), expected benefits of initiating opioids are unlikely to outweigh risks regardless of previous nonpharmacologic and nonopioid pharmacologic therapies used.

Clinical evidence reviews found no instrument with high accuracy for predicting opioid-related harms, such as overdose or opioid use disorder (*7*). For clinicians, predicting whether benefits of opioids for chronic pain will outweigh risks of ongoing opioid treatment for individual patients can be challenging. Therefore, opioid therapy should only be initiated with consideration by the clinician and patient of an exit strategy that could be used if opioid therapy is unsuccessful in improving pain and pain-related function.

Before opioid therapy is initiated for subacute or chronic pain, clinicians should determine with patients how functional benefit will be evaluated and establish treatment goals. Some patients have reported treatment goals are effective in increasing motivation and functioning (*7*). Goals ideally include improvement in function (including social, emotional, and physical dimensions), pain, and quality of life. Goals can be tailored to specific patient and clinical circumstances. For example, for some patients with diseases typically associated with progressive functional impairment or catastrophic injuries such as spinal cord trauma, reductions in pain without improvement in physical function might be more realistic. Clinicians can assess and then follow function, pain severity, and quality of life using tools such as the three-item PEG (Pain average, interference with Enjoyment of life, and interference with General activity) assessment scale (*184*) (see Recommendation 7). Clinically meaningful improvement has been defined as a 30% improvement in scores for both pain and function (*185*). Clinicians can ask patients about functional goals that have meaning for them (e.g., walking the dog or walking around the block, returning to part-time work, and attending family events or recreational activities), and then use these goals in assessing benefits of opioid therapy and weighing benefits against risks of continued opioid therapy for individual patients (see Recommendation 7).

Patients with subacute pain might be at a particularly critical point, both for potential transition to chronic pain and potential transition to long-term opioid therapy. Clinicians should reevaluate patients with subacute pain and their treatment course, ensure that potentially reversible causes of ongoing pain are addressed, and optimize pain management as needed. For patients with subacute pain who started opioid therapy for acute pain and have been treated with opioid therapy for ≥30 days, clinicians should ensure that opioid prescribing for acute pain does not unintentionally become long-term opioid prescribing simply because medications are continued without reassessment. Continuation of opioid therapy at this point might represent initiation of long-term opioid therapy, which should occur only as an intentional decision that benefits are likely to outweigh risks after informed discussion between the clinician and patient and as part of a comprehensive pain management approach.

Clinicians seeing new patients already using opioid medication should establish treatment goals, including functional goals, for continued opioid therapy. Clinicians should avoid rapid tapering or abrupt discontinuation of opioids (see Recommendation 5). Although the clinical evidence reviews did not find studies evaluating the effectiveness of written agreements or treatment plans (*7*), clinicians and patients who clearly document a treatment plan including specific functional goals in advance of prescribing will clarify expectations about how opioids will be prescribed and monitored with an aim to improve patient safety, health, and well-being.

Patient education and discussion before starting opioid therapy are critical so that patient preferences and values can be understood and used to inform clinical decisions. Clinicians should ensure that patients are aware of expected benefits of, common risks of, serious risks of, and alternatives to opioids before starting or continuing opioid therapy and should involve patients in decisions about whether to start opioid therapy. Many patients rank pain relief, nausea, vomiting, and constipation as important effects (*7*). The following elements are essential for communication and discussion with patients before starting opioid therapy:

- Review available low-cost options for pain management for all patients, and particularly for patients who have low incomes, do not have health insurance, or have inadequate insurance. Review considerations related to access to care because of the clinical oversight needed to initiate and continue opioid therapy and other treatments for pain.
- Be explicit and realistic about expected benefits of opioids, explaining that there is not robust evidence that opioids improve pain or function with long-term use and that complete elimination of pain is unlikely.
- Emphasize improvement in function as a primary goal and that function can improve even when pain is not eliminated.
- Advise patients about serious adverse effects of opioids, including potentially fatal respiratory depression and development of a potentially serious opioid use disorder that can cause distress and inability to fulfill major obligations at work, school, or home.
- Advise patients about common effects of opioids, such as constipation, dry mouth, nausea, vomiting, drowsiness, confusion, tolerance, physical dependence, and withdrawal symptoms when stopping opioids. To prevent constipation associated with opioid use, advise patients to increase hydration and fiber intake and to maintain or increase physical activity. Prophylactic pharmacologic therapy (e.g., a stimulant laxative such as senna, with or without a stool softener) is usually needed to ensure regular bowel movements if opioids are taken regularly. Stool softeners or fiber laxatives without another laxative should be avoided.
- If formulations are prescribed that combine opioids with acetaminophen, advise patients of the risks for taking additional over-the-counter products containing acetaminophen.
- Discuss effects that opioids might have on ability to safely operate a vehicle or other machinery, particularly when opioids are initiated, when dosages are increased, or when other central nervous system depressants, such as benzodiazepines or alcohol, are used concurrently.
- Discuss the potential for workplace toxicology testing programs to detect therapeutic opioid use.
- Discuss increased risks for opioid use disorder, respiratory depression, and death at higher dosages, along with the importance of taking only the amount of opioids prescribed (i.e., not taking more opioids than prescribed or taking them more often).
- Review increased risks for respiratory depression when opioids are taken with benzodiazepines, other sedatives, alcohol, nonprescribed drugs such as heroin, or other opioids.
- Discuss risks for household members and other persons if opioids are intentionally or unintentionally shared with others for whom they are not prescribed, including the possibility that others might experience overdose at the same or at lower dosage than prescribed for the patient and that young children are susceptible to unintentional ingestion. Discuss storage of opioids in a secure, preferably locked location and options for safe disposal of unused opioids (*154*).
- Discuss the importance of periodic reassessment to ensure that opioids are helping to meet patient goals and, if opioids are not effective or are harmful, to allow opportunities for consideration of opioid tapering and dosage reduction or discontinuation and of additional nonpharmacologic or nonopioid pharmacologic treatment options.
- Discuss expectations for clinician and patient responsibilities to mitigate risks of opioid therapy and planned use of precautions to reduce risks, including naloxone for overdose reversal (see Recommendation 8) and clinician use of PDMP information (see Recommendation 9) and toxicology screening (see Recommendation 10).
- Consider whether cognitive status might interfere with management of opioid therapy and, if so, determine whether a caregiver can responsibly comanage medication therapy. Discuss the importance of reassessing medication use over time with both the patient and caregiver, as appropriate.

Because of the possibility that benefits of opioid therapy might diminish or that risks might become more prominent over time, clinicians should elicit patients’ experiences and preferences and review expected benefits and risks of continued opioid therapy with patients periodically (see Recommendation 7).

#### Interventional Approaches to Subacute and Chronic Pain

Office-based interventional approaches, such as arthrocentesis and intra-articular glucocorticoid injection for pain associated with rheumatoid arthritis (*186*) or osteoarthritis (*187*) and subacromial corticosteroid injection for rotator cuff disease (*188*), can provide short-term improvement in pain and function to supplement or facilitate exercise, physical therapy, and other conservative approaches. Evidence is insufficient to determine the extent to which repeated glucocorticoid injection increases potential risks such as articular cartilage changes (in osteoarthritis) and sepsis (*187*).

Interventional pain management specialists offer additional interventions that can alleviate pain as part of a comprehensive pain management approach (*6*) for patients with indications including back pain, persistent pain after spinal surgery, neuropathic pain, and complex regional pain syndrome. Certain more common procedures include epidural steroid injections (for lumbar radiculopathy with herniated disc), nerve ablation procedures (e.g., radiofrequency denervation for low back pain), and neurostimulation procedures (e.g., peripheral nerve stimulation and spinal cord stimulation). Descriptions of common interventional procedures are available (*6*). Level of evidence for effectiveness and risks varies by procedure, and additional research is needed to establish the clinical benefits as well as risks of specific interventional procedures for specific pain conditions (*6*,*189*) compared with risks of opioid pain medications and other pharmacologic therapies. Rare, serious adverse events have been reported with epidural injection (*190*). Interventional procedures should be performed by properly trained clinicians following meticulous infection control protocols. Clinicians can consult with a qualified pain management specialist who is well versed in benefits and risks of diagnostic and therapeutic options to determine potential appropriateness of specific interventional procedures for their patients’ indications and clinical circumstances.

#### Multimodal Therapy for Subacute and Chronic Pain

Integrated pain management requires coordination of medical, psychological, and social aspects of health care and includes primary care, mental and behavioral health care, and specialist services when needed (*191*). Multimodal therapies and multidisciplinary biopsychosocial rehabilitation (e.g., combining psychological therapies with exercise) can reduce long-term pain and disability compared with usual care and compared with physical treatments (e.g., exercise) alone. Nonpharmacologic therapies also can provide synergistic benefits when nonopioid or opioid pain medications are used (*6*). When needed, medications should ideally be combined with nonpharmacologic therapy to provide greater benefits to patients in improving pain and function. Multimodal therapies are not always available or reimbursed by insurance and can be time consuming and costly for patients, and disparities in abilities to access multimodal care exist (*6*). Evidence exists that less-intensive multidisciplinary rehabilitation can be similarly effective to high-intensity multidisciplinary rehabilitation (*9*). Multimodal therapies should be considered for patients not responding to single-modality therapy, and combinations should be tailored depending on patient needs, cost, convenience, and other individual factors.

Depending on patient comorbidities and benefit-to-risk ratios in individual patients, combinations of medications (e.g., two nonopioid medications with different mechanisms of action or a nonopioid with an opioid medication) also might be used. In some cases, medication combinations might provide complementary or synergistic benefits and facilitate lower dosing of individual medications, as has been demonstrated in trials of patients with neuropathic pain (*7*). However, this approach should be used with caution to avoid synergistic risks of medications. For example, combinations of medications that depress the central nervous system and cause sedation (see Recommendation 11), such as an opioid with gabapentin, have been associated with increased risk for overdose compared with either medication alone (*7*).

### Selecting Opioids and Determining Opioid Dosages

#### Recommendation 3

**When starting opioid therapy for acute, subacute, or chronic pain, clinicians should prescribe immediate-release opioids instead of extended-release and long-acting (ER/LA) opioids (recommendation category: A; evidence type: 4).**

##### Implementation Considerations

- Clinicians should not treat acute pain with ER/LA opioids or initiate opioid treatment for subacute or chronic pain with ER/LA opioids, and clinicians should not prescribe ER/LA opioids for intermittent or as-needed use.
- ER/LA opioids should be reserved for severe, continuous pain. FDA has noted that some ER/LA opioids should be considered only for patients who have received certain dosages of opioids of immediate-release opioids daily for at least 1 week.
- When changing to an ER/LA opioid for a patient previously receiving a different immediate-release opioid, clinicians should consult product labeling and reduce total daily dosage to account for incomplete opioid cross-tolerance.
- Clinicians should use additional caution with ER/LA opioids and consider a longer dosing interval when prescribing to patients with renal or hepatic dysfunction because decreased clearance of medications among these patients can lead to accumulation of drugs to toxic levels and persistence in the body for longer durations.
- Methadone should not be the first choice for an ER/LA opioid. Only clinicians who are familiar with methadone’s unique risk profile and who are prepared to educate and closely monitor their patients, including assessing risk for QT prolongation and considering electrocardiographic monitoring, should consider prescribing methadone for pain.
- Only clinicians who are familiar with the dosing and absorption properties of the ER/LA opioid transdermal fentanyl and are prepared to educate their patients about its use should consider prescribing it.

##### Supporting Rationale

ER/LA opioids include methadone, transdermal fentanyl, and extended-release versions of opioids such as oxycodone, hydromorphone, hydrocodone, and morphine. Clinical evidence reviews found that effects of opioids on short-term pain and function were generally consistent across duration of action (short- or long-acting) and opioid type (opioid agonist, partial agonist, or mixed mechanism [with mixed opioid and nonopioid mechanisms of action] agent), although five trials directly comparing different types of opioids found a mixed mechanism agent associated with greater pain relief versus a pure opioid agonist, with fewer nonserious adverse events (*7*). A fair-quality study demonstrated a higher risk for overdose among patients treated with ER/LA opioids than among those treated with immediate-release opioids, especially within the first 2 weeks of therapy, with relative risk decreasing with longer duration of exposure (*7*,*192*). Clinical evidence reviews did not find evidence that continuous, time-scheduled use of ER/LA opioids is more effective or safer than intermittent use of immediate-release opioids or that time-scheduled use of ER/ LA opioids reduces risk for opioid use disorder (*7*).

In 2014, FDA modified the labeling for ER/LA opioid pain medications, noting serious risks and recommending that ER/LA opioids be reserved for management of pain severe enough to require daily, around-the-clock, long-term opioid treatment when alternative treatment options (e.g., nonopioid analgesics or immediate-release opioids) are ineffective, not tolerated, or would be otherwise inadequate to provide sufficient management of pain and not used as as-needed pain relievers (*49*). FDA also noted that some ER/LA opioids are only appropriate for opioid-tolerant patients, defined as patients who have received certain dosages of opioids (e.g., 60 mg daily of oral morphine, 30 mg daily of oral oxycodone, or equianalgesic dosages of other opioids) for at least 1 week (*193*). Time-scheduled opioid use can be associated with greater total average daily opioid dosage compared with intermittent, as-needed opioid use (*194*). Technologies have been used to prevent manipulation intended to defeat extended-release properties of ER/LA opioids and to prevent opioid use by unintended routes of administration, such as intravenous injection of oral opioids. FDA guidance for industry on evaluation and labeling of these “abuse-deterrent” opioids (*195*) indicates that these technologies, although they are expected to make manipulation of opioids more difficult or reduce the potent effects of manipulation, do not prevent opioid misuse or overdose through oral intake (the most common route of opioid misuse) and can still be misused by nonoral routes. The “abuse-deterrent” label does not indicate that there is no risk for misuse or opioid use disorder. No studies were found in the clinical evidence reviews assessing the effectiveness of “abuse-deterrent” technologies as a risk mitigation strategy for deterring or preventing opioid misuse, opioid use disorder, or overdose (*7*). Experts from OWG agreed with the recommendation for clinicians to initiate opioid treatment with immediate-release opioids instead of with ER/LA opioids and said they appreciated discussion of the lack of evidence for “abuse-deterrent” formulations.

In comparing different ER/LA formulations, clinical evidence reviews found inconsistent results for overdose risk with methadone versus other ER/LA opioids used for chronic pain, with two cohort studies of Medicaid beneficiaries finding methadone associated with increased risk for overdose or all-cause deaths versus morphine and one cohort study of U.S. Department of Veterans Affairs patients finding methadone to be associated with decreased risk (*7*). Methadone has been associated with disproportionate numbers of overdose deaths relative to the frequency with which it is prescribed for pain (*196*). In addition, methadone is associated with cardiac arrhythmias along with QT prolongation on the electrocardiogram, and it has complicated pharmacokinetics and pharmacodynamics, including a long and variable half-life and peak respiratory depressant effect occurring later and lasting longer than peak analgesic effect (*197*–*199*). In regard to other ER/LA opioid formulations, the absorption and pharmacodynamics of transdermal fentanyl are complex, with gradually increasing serum concentration during the first part of the 72-hour dosing interval, and variable absorption affected by factors such as external heat. In addition, the dosing of transdermal fentanyl is in mcg/hour, which is not typical for a drug used by outpatients and can be confusing. These complexities might increase the risk for fatal overdose when methadone or transdermal fentanyl is prescribed.

Clinicians should not treat acute pain with ER/LA opioids or initiate opioid treatment for subacute or chronic pain with ER/LA opioids, and clinicians should not prescribe ER/LA opioids for intermittent use. Because of the longer half-life and longer duration of effects (e.g., respiratory depression) of ER/LA opioids (e.g., methadone, fentanyl patches, or extended-release versions of oxycodone, hydromorphone, hydrocodone, or morphine), clinicians should not prescribe ER/LA opioids for the treatment of acute pain. ER/LA opioids should be reserved for severe, continuous pain and should be considered only for patients who have received certain dosages of immediate-release opioids daily (e.g., 60 mg daily of oral morphine, 30 mg daily of oral oxycodone, or equianalgesic dosages of other opioids) for at least 1 week (*193*). When changing to an ER/LA opioid for a patient previously receiving a different immediate-release opioid, clinicians should consult product labeling and reduce total daily dosage to account for incomplete opioid cross-tolerance. Clinicians should use additional caution with ER/LA opioids and consider a longer dosing interval when prescribing to patients with renal or hepatic dysfunction because decreased clearance of medications among these patients can lead to accumulation of medications to toxic levels and persistence in the body for longer durations. Although in certain situations clinicians might need to prescribe immediate-release and ER/LA opioids together (e.g., when transitioning patients from ER/LA opioids to immediate-release opioids by temporarily using lower dosages of both, for temporary postoperative use of short-term opioids in a patient already receiving ER/LA opioids, or in patients with opioid use disorder treated and stabilized on methadone who need short-acting opioids for acute pain), clinicians should consider the potential for increased overdose risk and use caution when prescribing immediate-release opioids in combination with ER/LA opioids.

When an ER/LA opioid is prescribed, using one with predictable pharmacokinetics and pharmacodynamics is preferred to minimize unintentional overdose risk. In particular, unique characteristics of methadone and transdermal fentanyl make safe prescribing of these medications for pain especially challenging. Methadone should not be the first choice for an ER/LA opioid. Only clinicians who are familiar with methadone’s unique risk profile and who are prepared to educate and closely monitor their patients, including risk assessment for QT prolongation and consideration of electrocardiographic monitoring, should consider prescribing methadone for pain. A clinical practice guideline regarding methadone prescribing for pain has been published previously (*200*). Because dosing effects of transdermal fentanyl often are misunderstood by both clinicians and patients, only clinicians who are familiar with its dosing and absorption properties of and are prepared to educate their patients about its use should consider prescribing transdermal fentanyl.

#### Recommendation 4

**When opioids are initiated for opioid-naïve patients with acute, subacute, or chronic pain, clinicians should prescribe the lowest effective dosage. If opioids are continued for subacute or chronic pain, clinicians should use caution when prescribing opioids at any dosage, should carefully evaluate individual benefits and risks when considering increasing dosage, and should avoid increasing dosage above levels likely to yield diminishing returns in benefits relative to risks to patients (recommendation category: A; evidence type: 3).**

##### Implementation Considerations

- The recommendations related to opioid dosages are not intended to be used as an inflexible, rigid standard of care; rather, they are intended to be guideposts to help inform clinician-patient decision-making. Risks of opioid use, including risk for overdose and overdose death, increase continuously with dosage, and there is no single dosage threshold below which risks are eliminated. Therefore, the recommendation language emphasizes that clinicians should avoid increasing dosage above levels likely to yield diminishing returns in benefits relative to risks to patients rather than emphasizing a single specific numeric threshold. Further, these recommendations apply specifically to starting opioids or to increasing opioid dosages, and a different set of benefits and risks applies to reducing opioid dosages (see Recommendation 5).
- When opioids are initiated for opioid-naïve patients with acute, subacute, or chronic pain, clinicians should prescribe the lowest effective dosage.
- For patients not already taking opioids, the lowest effective dose can be determined using product labeling as a starting point with calibration as needed based on the severity of pain and other clinical factors such as renal or hepatic insufficiency (see Recommendation 8).
- The lowest starting dose for opioid-naïve patients is often equivalent to a single dose of approximately 5–10 MME or a daily dosage of 20–30 MME/day. A listing of common opioid medications and their doses in MME equivalents is provided (Table).

**TABLE:** Morphine milligram equivalent doses for commonly prescribed opioids for pain management

Opioid	Conversion factor*
Codeine	0.15
Fentanyl transdermal (in mcg/hr)	2.4
Hydrocodone	1.0
Hydromorphone	5.0
Methadone	4.7
Morphine	1.0
Oxycodone	1.5
Oxymorphone	3.0
Tapentadol^†^	0.4
Tramadol^§^	0.2

**Sources:** Adapted from Von Korff M, Saunders K, Ray GT, et al. Clin J Pain 2008;24:521–7 and Nielsen S, Degenhardt L, Hoban B, Gisev N. Pharmacoepidemiol Drug Saf 2016;25:733–7.

**Abbreviations:** mcg/hr = microgram per hour; mg = milligram; MME = morphine milligram equivalent.

* Multiply the dose for each opioid by the conversion factor to determine the dose in MMEs. For example, tablets containing hydrocodone 5 mg and acetaminophen 325 mg taken four times a day would contain a total of 20 mg of hydrocodone daily, equivalent to 20 MME daily; extended-release tablets containing oxycodone 10 mg and taken twice a day would contain a total of 20 mg of oxycodone daily, equivalent to 30 MME daily. The following cautions should be noted: 1) All doses are in mg/day except for fentanyl, which is mcg/hr. 2) Equianalgesic dose conversions are only estimates and cannot account for individual variability in genetics and pharmacokinetics. 3) Do not use the calculated dose in MMEs to determine the doses to use when converting one opioid to another; when converting opioids, the new opioid is typically dosed at a substantially lower dose than the calculated MME dose to avoid overdose because of incomplete cross-tolerance and individual variability in opioid pharmacokinetics. 4) Use particular caution with methadone dose conversions because methadone has a long and variable half-life, and peak respiratory depressant effect occurs later and lasts longer than peak analgesic effect. 5) Use particular caution with transdermal fentanyl because it is dosed in mcg/hr instead of mg/day, and its absorption is affected by heat and other factors. 6) Buprenorphine products approved for the treatment of pain are not included in the table because of their partial *µ*-receptor agonist activity and resultant ceiling effects compared with full *µ*-receptor agonists. 7) These conversion factors should not be applied to dosage decisions related to the management of opioid use disorder.

^†^ Tapentadol is a *µ*-receptor agonist and norepinephrine reuptake inhibitor. MMEs are based on degree of *µ*-receptor agonist activity; however, it is unknown whether tapentadol is associated with overdose in the same dose-dependent manner as observed with medications that are solely *µ*-receptor agonists.

^§^ Tramadol is a *µ*-receptor agonist and norepinephrine and serotonin reuptake inhibitor. MMEs are based on degree of *µ*-receptor agonist activity; however, it is unknown whether tramadol is associated with overdose in the same dose-dependent manner as observed with medications that are solely *µ*-receptor agonists.
- If opioids are continued for subacute or chronic pain, clinicians should use caution when prescribing opioids at any dosage and should generally avoid dosage increases when possible.
- Many patients do not experience benefit in pain or function from increasing opioid dosages to ≥50 MME/day but are exposed to progressive increases in risk as dosage increases. Therefore, before increasing total opioid dosage to ≥50 MME/day, clinicians should pause and carefully reassess evidence of individual benefits and risks. If a decision is made to increase dosage, clinicians should use caution and increase dosage by the smallest practical amount. The recommendations related to opioid dosages are not intended to be used as an inflexible, rigid standard of care; rather, they are intended to be guideposts to help inform clinician-patient decision-making.
- Additional dosage increases beyond 50 MME/day are progressively more likely to yield diminishing returns in benefits for pain and function relative to risks to patients as dosage increases further. Clinicians should carefully evaluate a decision to further increase dosage on the basis of individualized assessment of benefits and risks and weighing factors such as diagnosis, incremental benefits for pain and function relative to risks with previous dosage increases, other treatments and effectiveness, and patient values and preferences. The recommendations related to opioid dosages are not intended to be used as an inflexible, rigid standard of care; rather, they are intended to be guideposts to help inform clinician-patient decision-making.

##### Supporting Rationale

Benefits of high-dose opioids for pain are not well established. Few trials evaluated opioid dosages of ≥90 MME/day (*7*). Opioid dosages of 50–90 MME/day were associated with a minimally greater (below the threshold for small) improvement in mean pain intensity compared with dosages of <50 MME/day (mean difference: −0.26; 95% CI: −0.57 to −0.02); there was no difference in mean improvement in function (*7*). Analyses of placebo-controlled trials also found some evidence of a plateauing effect at ≥50 mg MME/day (*7*). One trial of more liberal dose escalation compared with maintenance of current dosage found no difference in outcomes related to pain or function (*7*).

At the same time, risks for serious harms related to opioid therapy, including opioid misuse, overdose, and death, increase at higher opioid dosage, without a single point below which there is no risk (*201*). One cohort study from the clinical evidence reviews found higher dosages of opioids were associated with increased risk for all-cause deaths; one cohort study found modest associations between higher dose of long-term opioid and increased risk for falls and major trauma; one case-control study found opioid dosages of >20 MME/day were associated with increased odds of road trauma injury when the analysis was restricted to drivers, with no dose-dependent association at dosages of >20 MME/day; and cohort studies found association between higher opioid dose and risk for various endocrinological adverse events (*7*). Patients on higher doses reported reliance on opioids despite ambivalence about their benefits (*7*).

Four observational studies identified in the clinical evidence reviews consistently found an association between higher doses of long-term opioids and risk for overdose or overdose death (*7*). Opioid dosages for chronic pain of 50 to <100 MME/day in observational studies have been associated with increased risks for opioid overdose by factors of 1.9–4.6 compared with dosages of 1 to <20 MME/day, and dosages of ≥100 MME/day were found to be associated with increased risks for overdose 2.0–8.9 times the risk at 1 to <20 MME/day, after adjusting for confounders on the basis of demographics, comorbidities, concomitant medications, and other factors (*55*,*202*,*203*). When opioids are prescribed for acute pain, similar associations have been found, with dosages of 50 to <100 MME/day associated with 4.73 times the risk for overdose and dosages of ≥100 MME/day associated with 6.64 times the risk, compared with dosages of 1 to <20 MME/day (*55*). The MME cut points in these studies (e.g., 20 MME, 50 MME, and 100 MME) were selected by the authors for research purposes, and whereas their findings are consistent with progressive increases in overdose risk being associated with increases in prescribed opioid dosages, they do not demonstrate a specific dosage threshold below which opioids are never associated with overdose. In a national sample of Veterans Health Administration patients with chronic pain who were prescribed opioids, mean prescribed daily opioid dosage among patients who died from opioid overdose was 98 MME (median: 60 MME), compared with mean prescribed daily opioid dosage of 48 MME (median: 25 MME) among patients not experiencing fatal overdose (*204*). A narrative review conducted by FDA staff concluded that, although there is not a single dosage threshold below which overdose risk is eliminated (*201*), the studies included in the review indicated an increasing risk for serious adverse health outcomes, including misuse, overdose, and death associated with increasing opioid dose. These studies examined dose-response risk for overdose for full agonist opioids and not for partial agonist opioids such as buprenorphine, which is unlikely to have the same continuous association between dosage and overdose risk because respiratory depressant effects of buprenorphine reach a plateau (*205*).

Multiple experts from OWG expressed concern that including specific dosage thresholds in a main recommendation statement would emphasize them as authoritative absolutes and would lead to noncollaborative tapers or other potentially harmful consequences. Experts also noted the lack of a single standard formula for calculating MMEs (*206*). However, experts agreed there is a need for thresholds as benchmarks and suggested including them in the supporting text after the main recommendation statement. Experts also agreed with separating recommendations on dosage into a recommendation applying to patients starting opioids and patients already receiving opioids.

When opioids are used for acute, subacute, or chronic pain, clinicians should start opioids at the lowest possible effective dosage. For patients not already taking opioids, the lowest effective dose can be determined using product labeling as a starting point with calibration as needed on the basis of the severity of pain and other clinical factors, such as renal or hepatic insufficiency (see Recommendation 8). The lowest starting dose for opioid-naïve patients is often equivalent to a single dose of approximately 5–10 MME or a daily dosage of 20–30 MME/day. A listing of common opioid medications and their doses in MME equivalents is provided (Table). For example, a label for hydrocodone bitartrate (5 mg) and acetaminophen (300 mg) (*207*) states that the usual adult dosage is one or two tablets every 4–6 hours as needed for pain, and the total daily dosage should not exceed eight tablets. Clinicians should use additional caution when initiating opioids for patients aged ≥65 years and patients with renal or hepatic insufficiency because of a potentially smaller therapeutic window between safe dosages and dosages associated with respiratory depression and overdose (see Recommendation 8). Formulations with lower opioid doses (e.g., hydrocodone bitartrate 2.5 mg/acetaminophen 325 mg) are available and can facilitate dosing when additional caution is needed. Product labeling regarding tolerance includes guidance for patients already taking opioids. In addition to opioids, clinicians should consider cumulative dosages of other medications, such as acetaminophen, that are combined with opioids in many formulations and for which decreased clearance of medications might result in accumulation of medications to toxic levels.

Clinicians should generally avoid unnecessary dosage increases, use caution when increasing opioid dosages, and increase dosage by the smallest practical amount because overdose risk increases with increases in opioid dosage. Although evidence to recommend specific intervals for dosage titration is limited, rapid dosage increases put patients at greater risk for sedation, respiratory depression, and overdose. For opioid-naïve outpatients with acute pain treated with an opioid for a few days or less, dosage increases are usually unnecessary and should not be attempted without close monitoring because of the risks for respiratory depression. In the context of long-term opioid use, when dosage is increased, clinicians should reevaluate patients after increasing dosage for changes in pain, function, and risk for harm (see Recommendation 7).

Before increasing total opioid dosage to ≥50 MME/day, clinicians should pause, considering that dosage increases to >50 MME/day are unlikely to provide substantially improved pain control for most patients while overdose risk increases with dosage, and carefully reassess evidence of benefits and risks. If a patient’s opioid dosage for all sources of opioids combined reaches or exceeds 50 MME/day, clinicians should implement additional precautions, including increased frequency of follow-up (see Recommendation 7), and offer naloxone and overdose prevention education to both the patient and the patient’s household members (see Recommendation 8).

Additional dosage increases beyond 50 MME/day are progressively more likely to yield diminishing returns in benefits for pain and function relative to risks to patients. Clinicians should carefully evaluate a decision to increase dosage after an individualized assessment of benefits and risks and weighing factors such as diagnosis, incremental benefits for pain and function relative to risks with previous dosage increases, other treatments and effectiveness, and patient values and preferences.

Certain states require clinicians to implement clinical protocols at specific dosage levels. For example, before increasing long-term opioid therapy dosage to >120 MME/day, clinicians in Washington state must obtain consultation from a pain specialist who agrees that the increase is indicated and appropriate (*208*). Clinicians should be aware of policies related to MME thresholds and associated clinical protocols established by their states.

#### Recommendation 5

**For patients already receiving opioid therapy, clinicians should carefully weigh benefits and risks and exercise care when changing opioid dosage. If benefits outweigh risks of continued opioid therapy, clinicians should work closely with patients to optimize nonopioid therapies while continuing opioid therapy. If benefits do not outweigh risks of continued opioid therapy, clinicians should optimize other therapies and work closely with patients to gradually taper to lower dosages or, if warranted based on the individual circumstances of the patient, appropriately taper and discontinue opioids. Unless there are indications of a life-threatening issue such as warning signs of impending overdose (e.g., confusion, sedation, or slurred speech), opioid therapy should not be discontinued abruptly, and clinicians should not rapidly reduce opioid dosages from higher dosages (recommendation category: B; evidence type: 4).**

##### Implementation Considerations

- Clinicians should carefully weigh both the benefits and risks of continuing opioid medications and the benefits and risks of tapering opioids.
- If benefits outweigh risks of continued opioid therapy, clinicians should work closely with patients to optimize nonopioid therapies while continuing opioid therapy.
- When benefits (including avoiding risks of tapering) do not outweigh risks of continued opioid therapy, clinicians should optimize other therapies and work closely with patients to gradually taper to a reduced opioid dosage or, if warranted based on the individual clinical circumstances of the patient, appropriately taper and discontinue opioid therapy.
- In situations where benefits and risks of continuing opioids are considered to be close or unclear, shared decision-making with patients is particularly important.
- At times, clinicians and patients might not be able to agree on whether or not tapering is necessary. When patients and clinicians are unable to arrive at a consensus on the assessment of benefits and risks, clinicians should acknowledge this discordance, express empathy, and seek to implement treatment changes in a patient-centered manner while avoiding patient abandonment.
- Patient agreement and interest in tapering is likely to be a key component of successful tapers.
- For patients agreeing to taper to lower opioid dosages and for those remaining on higher opioid dosages, clinicians should establish goals with the patient for continued opioid therapy (see Recommendations 2 and 7) and maximize pain treatment with nonpharmacologic and nonopioid pharmacologic treatments as appropriate (see Recommendation 2).
- Clinicians should collaborate with the patient on the tapering plan, including patients in decisions such as how quickly tapering will occur and when pauses in the taper might be warranted.
- Clinicians should follow up frequently (at least monthly) with patients engaging in opioid tapering. Team members (e.g., nurses, pharmacists, and behavioral health professionals) can support the clinician and patient during the ongoing taper process through telephone contact, telehealth visits, or face-to-face visits.
- When opioids are reduced or discontinued, a taper slow enough to minimize symptoms and signs of opioid withdrawal (e.g., anxiety, insomnia, abdominal pain, vomiting, diarrhea, diaphoresis, mydriasis, tremor, tachycardia, or piloerection) should be used.
- Longer duration of previous opioid therapy might require a longer taper. For patients who have taken opioids long-term (e.g., for ≥1 year), tapers can be completed over several months to years depending on the opioid dosage and should be individualized based on patient goals and concerns.
- When patients have been taking opioids for longer durations (e.g., for ≥1 year), tapers of 10% per month or slower are likely to be better tolerated than more rapid tapers.
- For patients struggling to tolerate a taper, clinicians should maximize nonopioid treatments for pain and should address behavioral distress.
- Clinically significant opioid withdrawal symptoms can signal the need to further slow the taper rate.
- At times, tapers might have to be paused and restarted again when the patient is ready and might have to be slowed as patients reach low dosages.
- Before reversing a taper, clinicians should carefully assess and discuss with the patient the benefits and risks of increasing opioid dosage.
- Goals of the taper might vary (e.g., some patients might achieve discontinuation whereas others might attain a reduced dosage at which functional benefits outweigh risks). If the clinician has determined with the patient that the ultimate goal of tapering is discontinuing opioids, after the smallest available dose is reached the interval between doses can be extended and opioids can be stopped when taken less frequently than once a day.
- Clinicians should access appropriate expertise if considering tapering opioids during pregnancy because of possible risks to the pregnant patient and the fetus if the patient goes into withdrawal.
- Clinicians should advise patients of an increased risk for overdose on abrupt return to a previously prescribed higher dose because of loss of opioid tolerance, provide opioid overdose education, and offer naloxone.
- Clinicians should remain alert to signs of and screen for anxiety, depression, and opioid misuse or opioid use disorder (see Recommendations 8 and 12) that might be revealed by an opioid taper and provide treatment or arrange for management of these comorbidities.
- Clinicians should closely monitor patients who are unable to taper and who continue on high-dose or otherwise high-risk opioid regimens (e.g., opioids prescribed concurrently with benzodiazepines) and should work with patients to mitigate overdose risk (e.g., by providing overdose education and naloxone) (see Recommendation 8).
- Clinicians can use periodic and strategic motivational questions and statements to encourage movement toward appropriate therapeutic changes and functional goals.
- Clinicians have a responsibility to provide or arrange for coordinated management of patients’ pain and opioid-related problems, including opioid use disorder.
- Payers, health systems, and state medical boards should not use this clinical practice guideline to set rigid standards or performance incentives related to dose or duration of opioid therapy; should ensure that policies based on cautionary dosage thresholds do not result in rapid tapers or abrupt discontinuation of opioids; and should ensure that policies do not penalize clinicians for accepting new patients who are using prescribed opioids for chronic pain, including those receiving high dosages of opioids, or for refraining from rapidly tapering patients prescribed long-term opioid medications.
- Although Recommendation 5 specifically refers to patients using long-term opioid therapy for subacute or chronic pain, many of the principles in these implementation considerations and supporting rationale, including communication with patients, pain management, behavioral support, and slower taper rates, also are relevant when discontinuing opioids in patients who have received them for shorter durations (see Recommendations 6 and 7).

##### Supporting Rationale

Patients receiving long-term, high-dosage opioid therapy for chronic pain are at increased risk for adverse events including overdose death (*55*,*72*,*202*,*203*,*209*). However, discontinuation of long-term, high-dosage opioid therapy has been associated with adverse events including mental health crisis, overdose events, and overdose death (*71*–*73*,*210*,*211*). In addition, opioid tapering has been found to be associated with subsequent termination of care (*212*). One study found that whereas sustained opioid therapy discontinuation (i.e., opioid discontinuation for at least 3 months) was associated with an approximately 50% reduction in risk for overdose, dose variability was a risk factor for opioid overdose (*213*). In another study, discontinuation of long-term, high-dosage opioid therapy was associated with increased risk for suicide but with reduced risk for overdose when compared with stable or increasing dosage (*211*). Both starting and stopping opioids were associated with overdose or suicide risk in another study; risk associated with stopping opioids was increased when patients had received opioids for longer durations. Death rates for overdose or suicide in one study increased immediately after starting or stopping treatment with opioids, with the incidence decreasing over approximately 3–12 months (*214*) in one study and persisting over 2 years in another study (*215*). In observational studies evaluating outcomes related to heroin use after discontinuation of prescription opioids, one study found that heroin use was associated with discontinuation of long-term opioid use (*216*); another study found that among persons experiencing heroin overdose, prescription opioid use in the past 12 months was common but discontinuation of long-term opioid use was uncommon (*217*).

Discontinuation of opioids has been associated with greater risks when it occurs over shorter periods. FDA has advised that risks of rapid tapering or sudden discontinuation of opioids in physically dependent patients include acute withdrawal symptoms, exacerbation of pain, serious psychological distress, and thoughts of suicide (*68*). One observational study found that, among adults prescribed stable higher opioid dosages (mean: ≥50 MME/day) long-term, increasing maximum monthly dose reduction rate by 10% was associated with an adjusted incidence rate ratio of 1.09 for overdose (95% CI: 1.07–1.11) and 1.18 for mental health crisis (95% CI: 1.14–1.21) (*210*). Another study of patients on long-term, high-dosage (≥120 MME/day) opioid therapy found that each additional week of tapering time before opioid discontinuation was associated with a 7% relative reduction in the risk for opioid-related emergency department visits or hospitalizations (*71*). The clinical evidence reviews did not find studies comparing different rates of opioid tapering; however, a taper support intervention (psychiatric consultation, opioid dosage tapering, and 18 weekly meetings with a physician assistant to explore motivation for tapering and learn pain self-management skills) was associated with better functional outcomes (specifically, improvement in pain interference) compared with usual care, with effects persisting at 34-week follow-up (*7*). A systematic review (*218*) found that, among studies rated as good or fair quality, when opioids were tapered after discussion with patients who agreed to taper, opioid dose reduction was associated with improved pain, function, and quality of life. These results suggest that involving patients in decisions regarding continuation or discontinuation of opioid medications as well as practices including behavioral support, integration of nonpharmacologic pain management, and slower tapers might improve outcomes.

Experts from OWG said they appreciated the complexity of managing patients already receiving higher dosages of opioids long-term. Although some experts indicated there should be more consideration of obtaining informed consent before tapering opioids, others believed that informed discussion is more appropriate than informed consent when considering tapering opioids because of clinicians’ overriding responsibility to avoid providing treatment that harms patients. Some experts were concerned that overemphasizing risks of tapering could increase harm from continued high-dosage opioid use.

#### Determining Whether, When, and How to Taper Opioids

The benefits and risks of opioid therapy change over time and should be reevaluated periodically (see Recommendations 6 and 7). Opioid therapy should be limited to circumstances where benefits of therapy outweigh risks. Because tapering opioids can be harmful in some circumstances, benefits of continuing opioids in patients who have already received them long-term might include avoiding risks of tapering and discontinuing opioids. In situations where benefits and risks of continuing opioids are considered to be close or unclear, shared decision-making with patients is particularly important. At times, clinicians and patients might not be able to agree on whether tapering is necessary. When patients and clinicians are unable to arrive at a consensus on the assessment of benefits and risks, clinicians should acknowledge this discordance, express empathy, and seek to implement treatment changes in a patient-centered manner while avoiding patient abandonment. Unless there is a life-threatening issue such as warning signs of an imminent overdose, the benefits of rapidly tapering or abruptly discontinuing opioids are unlikely to outweigh the substantial risks of these practices (*71*,*219*). However, after slow, voluntary reduction of long-term opioid dosages, patients might experience improvements in function, quality of life, anxiety, and mood without worsening pain or with decreased pain levels (*218*). Clinicians and patients should consider whether opioids continue to meet treatment goals, including functional goals; whether opioids are exposing the patient to an increased risk for serious adverse events or opioid use disorder; and whether benefits continue to outweigh risks of opioids. Clinicians should not insist on opioid tapering or discontinuation when opioid use might be warranted (i.e., when benefits of opioids outweigh risks) (*66*,*219*). Clinicians should access appropriate expertise if considering tapering opioids during pregnancy because of possible risk to the pregnant patient and the fetus if the patient goes into withdrawal. For pregnant persons with opioid use disorder, medications for opioid use disorder are preferred over withdrawal management (i.e., discontinuation of opioids through either short- or medium-term tapering) (*220*,*221*).

Some patients using more than one respiratory depressant (e.g., benzodiazepines and opioids) might require tapering one or more medications to reduce risk for respiratory depression. Tapering decisions and plans should be coordinated with prescribers of all respiratory depressant medications (see Recommendation 11). Benzodiazepines should be tapered gradually because of risks (anxiety, hallucinations, seizures, delirium tremens, and, rarely, death) of benzodiazepine withdrawal (*222*,*223*). Patients who are not taking prescribed opioids (e.g., patients who are diverting all opioids they obtain) do not require tapers.

Consistent with the HHS Guide for Clinicians on the Appropriate Dosage Reduction or Discontinuation of Long-Term Opioid Analgesics (*219*), clinicians should consider tapering to a reduced opioid dosage or tapering and discontinuing opioid therapy and discuss these approaches with patients before initiating changes when

- the patient requests dosage reduction or discontinuation,
- pain improves and might indicate resolution of an underlying cause,
- opioid therapy has not meaningfully reduced pain or improved function,
- the patient has been treated with opioids for a prolonged period (e.g., years) and the benefit-risk balance is unclear (e.g., decreased positive effects because of tolerance and symptoms such as reduced focus or memory that might be due to opioids),
- the patient is receiving higher opioid dosages without evidence of benefit from the higher dosage,
- the patient experiences side effects that diminish quality of life or impair function,
- evidence of opioid misuse exists,
- the patient experiences an overdose or other serious event (e.g., an event leading to hospitalization or injury) or has warning signs for an impending event (e.g., confusion, sedation, or slurred speech), or
- the patient is receiving medications (e.g., benzodiazepines) or has medical conditions (e.g., sleep apnea, liver disease, kidney disease, or fall risk) that increase risk for adverse outcomes.

For patients already taking opioids long term (both established patients and patients transferring from other clinicians), the possibility of opioid dosage reduction might provoke substantial anxiety. In addition, tapering opioids after years of taking them can be especially challenging because of physical and psychological dependence. However, patients should be offered the opportunity to reevaluate their continued use of opioids. Clinicians should review benefits and risks of continued opioid therapy with empathy.

Whenever possible, clinicians should collaborate with patients and share decision-making about whether and how to taper opioids. Clinicians should review benefits and risks of opioid therapy with the patient and decide whether tapering is appropriate for the patient. If the existing opioid regimen does not put the patient at imminent risk for overdose or other injury, tapering does not need to occur immediately, and clinicians can take time to reach agreement with patients (*224*). For patients who agree to taper opioids to lower dosages, clinicians should collaborate with the patient on a tapering plan. Open discussion between the clinician and patient should take place, whether the goal of the taper is stopping opioids or reducing opioids to a point where benefits outweigh risks; the goal will depend on the patient’s circumstances and an individualized assessment of benefits and risks. Tapering is more likely to be successful when patients collaborate in the taper (*224*). Clinicians can discuss with patients the patient’s perceptions of benefits, risks, and adverse effects of continued opioid therapy; include patient concerns in taper planning; and include patients in making decisions such as which medication will be decreased first (e.g., in patients prescribed more than one opioid) and how quickly tapering will occur.

#### Providing Advice to Patients Before Tapering

Clinicians should advise patients that overall, after voluntary reduction of long-term opioid dosages, most patients report stable or improved function, anxiety, and mood without worsening pain or with decreased pain levels (*66*,*218*,*225*–*228*). However, other patients report insomnia, anxiety, depression, and increased pain, particularly in the short term (*66*,*225*,*227*,*229*,*230*). Increased pain might be related to hyperalgesia or opioid withdrawal and can be prolonged in some patients (*229*). Patients can be counseled that worsening of pain is a frequent symptom of opioid withdrawal that tends to diminish over time (*219*). Clinicians should advise patients about the increased risk for overdose with abrupt return to a previously prescribed higher dosage because of loss of opioid tolerance and warn of a risk for overdose if the patient returns to their original dosage (*219*). Clinicians should provide opioid overdose education and offer naloxone.

#### Pain Management During Tapering

Clinicians should commit to working with patients to improve function and decrease pain, whether or not opioids are tapered. Nonpharmacologic and nonopioid treatments should be integrated into patients’ pain management plans after an individualized assessment of benefits and risks that considers the patient’s diagnosis, circumstances, and unique needs (see Recommendation 2). Integrating behavioral and nonopioid pain therapies before and during a taper can help manage pain (*218*) and strengthen the therapeutic relationship between the clinician and patient. Whether patients are agreeing to taper to lower opioid dosages or remaining on higher opioid dosages, clinicians should work with them to establish functional goals for continued opioid therapy (see Recommendations 2 and 7) and maximize pain treatment with nonpharmacologic and nonopioid pharmacologic treatments as appropriate (see Recommendation 2).

#### Behavioral Health Support During Tapering

Integrating behavioral and nonopioid pain therapies and treatment for comorbid mental health conditions before and during a taper can help manage pain (*218*), strengthen the therapeutic relationship between the clinician and patient, and improve the likelihood of positive tapering outcomes (*228*). Mental health comorbidities including depression and anxiety are common in patients with painful conditions, especially those receiving long-term opioid therapy (*231*). Depressive symptoms predict taper dropout (*225*,*226*). Primary care clinicians should collaborate with mental health specialists and with other specialty clinicians as needed to optimize nonopioid pain management (see Recommendation 2) and provide psychosocial support for patients who have anxiety related to the taper. Clinicians should consider arranging for consultation with a behavioral health specialist before initiating a taper in patients with serious mental illness who are at high risk for suicide or with suicidal ideation (*219*). Clinicians should remain alert to signs of and screen for anxiety, depression, and opioid misuse or opioid use disorder (see Recommendations 8 and 12) that might be revealed by an opioid taper and provide treatment or arrange for management of these comorbidities. Successful tapering studies have used at least weekly follow-up (*218*), and clinicians should follow up frequently (at least monthly) with patients engaging in opioid tapering. Team members (e.g., nurses, pharmacists, and behavioral health professionals) can support the clinician and patient during the ongoing taper process through telephone contact, telehealth visits, or face-to-face visits. Clinicians can acknowledge patient fears about tapering (*232*), ask how they can support the patient (*232*), and make sure patients receive appropriate and accessible psychosocial support (*228*). Many patients fear withdrawal symptoms, pain, or abandonment (*233*), and clinicians can help patients by telling them what to expect (e.g., the rate will be kept slow to minimize withdrawal symptoms and pain might worsen at first but usually improves over time) and that they will be supporting them through the process.

#### Tapering Rate

Evidence to support specific tapering rates is limited. The rate of tapering should be individualized based on the patient’s clinical situation. When opioids are reduced or discontinued, a taper slow enough to minimize symptoms and signs of opioid withdrawal (e.g., anxiety, insomnia, abdominal pain, vomiting, diarrhea, diaphoresis, mydriasis, tremor, tachycardia, or piloerection) should be used. Tapers can be completed over several months to years depending on the opioid dosage and should be individualized based on patient goals and concerns. Longer durations of previous opioid therapy might require longer tapers. Evidence on optimal taper rate is emerging. Tapers of approximately 10% per month or slower are likely to be better tolerated than more rapid tapers when patients have been taking opioids for longer durations (e.g., ≥1 year) (*219*). When patients have taken opioids for shorter durations (e.g., weeks to months rather than years), a decrease of 10% of the original dose per week or slower (until approximately 30% of the original dose is reached, followed by a weekly decrease of approximately 10% of the remaining dose) is less likely to trigger withdrawal (*225*) and can be successful for some patients. For patients struggling to tolerate a taper, clinicians should maximize nonopioid treatments for pain and should address behavioral distress (*234*). Clinically significant opioid withdrawal symptoms can signal the need to further slow the taper rate. At times, tapers might have to be paused and restarted again when the patient is ready and might have to be slowed as patients reach low dosages to allow gradual accommodation to lower opioid dosages and development of new skills for nonopioid management of pain and emotional distress. Before reversing a taper, clinicians should carefully assess and discuss with patients benefits and risks of increasing opioid dosage. If the clinician and patient have determined that the goal is discontinuing opioids, after the smallest available dose is reached, the interval between doses can be extended and opioids can be stopped when taken less frequently than once a day.

More rapid tapers might be needed for patient safety under certain circumstances (e.g., for patients who have experienced overdose on their current dosage) (*219*). However, unless there are indications of a life-threatening issue, such as warning signs of impending overdose, opioid therapy should not be discontinued abruptly, and clinicians should not rapidly reduce opioid dosages from higher dosages. Sudden discontinuation might precipitate substantial opioid withdrawal (*71*). Rapid tapering or sudden discontinuation of opioids in physically dependent patients also can increase risks for psychological distress and opioid-related emergency department visits and hospitalizations (*68*,*71*). Ultrarapid detoxification under anesthesia is associated with substantial risks, including death, and should not be used (*235*).

#### Management of Opioid Withdrawal During Tapering

The first approach to withdrawal symptoms and signs should generally be consideration of slowing or pausing the taper rate. If needed, short-term oral medications might also help manage withdrawal symptoms (*232*). These include alpha-2 agonists for the management of autonomic signs and symptoms (e.g., sweating and tachycardia). Alpha-2 agonists clonidine and lofexidine are more effective than placebo in reducing severity of withdrawal (*236*) from heroin or methadone in the context of abrupt (not gradual) discontinuation. Similar research could not be found on clonidine and lofexidine in patients tapering from long-term opioid treatment for pain (*225*); however, alpha-2 agonist tizanidine has been used to help taper patients from long-term, high-dosage opioids for chronic pain (*230*). Other medications addressing specific symptoms (NSAIDs, acetaminophen, or topical menthol or methyl salicylate for muscle aches; trazodone for sleep disturbance; prochlorperazine, promethazine, or ondansetron for nausea; dicyclomine for abdominal cramping; and loperamide or bismuth subsalicylate for diarrhea) also have been used (*232*).

#### Challenges to Tapering

Some patients with unanticipated challenges to tapering, such as inability to make progress in tapering despite opioid-related harm, might have undiagnosed opioid use disorder. Therefore, patients experiencing such challenges should be assessed for opioid use disorder using *Diagnostic and Statistical Manual of Mental Disorders,* *Fifth Edition* (DSM-5) criteria and, if criteria for opioid use disorder are met, offered evidence-based medication treatment (see Recommendation 12) and naloxone for opioid overdose reversal (see Recommendation 8).

Emerging evidence suggests that patients for whom risks of continued high-dose opioid use outweigh benefits but who are unable to taper and who do not meet criteria for opioid use disorder might benefit from transition to buprenorphine (*219*,*237*,*238*). Buprenorphine is a partial agonist opioid that can treat pain and opioid use disorder (*239*) and has other properties that might be helpful (*155*), including less respiratory depression (*205*) and overdose risk than other opioids (*155*,*237*). Although overdose is less likely with buprenorphine than with full agonist opioids, overdose is still possible, particularly if buprenorphine is taken concurrently with other respiratory depressants (e.g., full agonist opioids, benzodiazepines, or alcohol) (*240*). A specialty clinic offering opioid tapering services for patients receiving high-dosage opioids (defined in this study as ≥90 MME/day) for chronic pain found that 44.6% of patients referred for opioid taper were able to successfully taper to <90 MME/day, and an additional 18.8% who were unable to taper were able to successfully transition to sublingual buprenorphine (*230*). Different buprenorphine products, available at different formulations and doses, are approved for the treatment of pain and for the treatment of opioid use disorder. Although prescription of buprenorphine for treatment of opioid use disorder requires the clinician to have a waiver from SAMHSA (see Recommendation 12), prescription of buprenorphine for treatment of chronic pain does not require a waiver (*237*).

To avoid precipitating withdrawal, transitioning any patient taking full agonist opioids to buprenorphine requires specific timing of the initial buprenorphine dose (*219*) (see Recommendation 12 for application to patients with opioid use disorder). Patients should be in mild to moderate withdrawal from full agonist opioids before the first buprenorphine dose (*219*). To do this, experts have advised that clinicians and patients should wait at least 8–12 hours after the last dose of short-acting full agonist opioids and longer after the last dose of long-acting full agonist opioids (e.g., at least 12–24 hours after the last dose of an ER/LA full agonist opioid, and longer for methadone) before the first dose of buprenorphine is administered (*229*). As an alternative for patients not yet in opioid withdrawal, certain studies have described low dose initiation of buprenorphine to allow for initiation of buprenorphine in patients receiving full agonist opioids for acute or chronic pain (*241*). SAMHSA’s Providers Clinical Support System (https://pcssnow.org) offers training, technical assistance, and mentors to assist clinicians who are unfamiliar with initiation of buprenorphine and have additional questions about the diagnosis and treatment of opioid use disorder. Because the duration of action for analgesia is shorter than the duration of action for suppression of opioid withdrawal and stabilization of opioid use disorder (*242*), dosing of buprenorphine for pain is typically multiple times daily rather than once-a-day dosing as done for the treatment of opioid use disorder (*229*).

#### Continuing High-Dosage Opioids

Clinicians should closely monitor patients who are unable to taper and who continue on high-dosage or otherwise high-risk opioid regimens (e.g., opioids prescribed concurrently with benzodiazepines) and should work with patients to mitigate overdose risk (e.g., by providing overdose education and naloxone) (see Recommendation 8). Clinicians can use periodic and strategic motivational questions and statements to encourage movement toward appropriate therapeutic changes (*224*).

Management of chronic pain with opioids can be challenging, as can management of opioid discontinuation (*67*). However, clinicians have a responsibility to provide or arrange for coordinated management of patients’ pain and opioid-related challenges. Payers and health systems should not use this clinical practice guideline to set rigid standards related to dosage or duration of opioid therapy and should ensure that policies based on cautionary dosage thresholds do not result in rapid tapers or abrupt discontinuation of opioids, do not penalize clinicians for accepting new patients who are receiving opioids for chronic pain, and do not provide incentives to clinicians to implement rapid tapering. Patients prescribed opioids but unable to access ongoing care (*243*) might be at risk for abrupt opioid discontinuation and might miss opportunities to receive life-saving interventions, including monitoring for and management of mental health and substance use comorbidities.

### Deciding Duration of Initial Opioid Prescription and Conducting Follow-Up

#### Recommendation 6

**When opioids are needed for acute pain, clinicians should prescribe no greater quantity than needed for the expected duration of pain severe enough to require opioids (recommendation category: A; evidence type: 4).**

##### Implementation Considerations

- Nontraumatic, nonsurgical acute pain can often be managed without opioids (see Recommendation 1).
- Opioids are sometimes needed for treatment of acute pain (see Recommendation 1). When the diagnosis and severity of acute pain warrant use of opioids, clinicians should prescribe no greater quantity than needed for the expected duration of pain severe enough to require opioids. For many common causes of nontraumatic, nonsurgical pain, when opioids are needed, a few days or less are often sufficient, and shorter courses can minimize the need to taper opioids to prevent withdrawal symptoms at the end of a course of opioids. However, durations should be individualized to the patient’s clinical circumstances.
- Clinicians should generally avoid prescribing additional opioids to patients just in case pain continues longer than expected.
- For postoperative pain related to major surgery, procedure-specific opioid prescribing recommendations are available with ranges for amounts of opioids needed (on the basis of actual use and refills and on consensus).
- To minimize unintended effects on patients, clinicians, practices, and health systems should have mechanisms in place for the subset of patients who experience severe acute pain that continues longer than the expected duration. These mechanisms should allow for timely reevaluation to confirm or revise the initial diagnosis and adjust pain management accordingly. Clinicians, practices, and health systems can help minimize disparities in access to and affordability of care and refills by ensuring all patients can obtain and afford additional evaluation and treatment, as needed.
- Longer durations of opioid therapy are more likely to be needed when the mechanism of injury is expected to result in prolonged severe pain (e.g., severe traumatic injuries).
- Patients should be evaluated at least every 2 weeks if they continue to receive opioids for acute pain.
- If opioids are continued for ≥1 month, clinicians should ensure that potentially reversible causes of chronic pain are addressed and that opioid prescribing for acute pain does not unintentionally become long-term opioid therapy simply because medications are continued without reassessment. Continuation of opioid therapy at this point might represent initiation of long-term opioid therapy, which should occur only as an intentional decision that benefits are likely to outweigh risks after discussion between the clinician and patient and as part of a comprehensive pain management approach. Clinicians should refer to recommendations on subacute and chronic pain for initiation (Recommendation 2), follow-up (Recommendation 7), and tapering (Recommendation 5) of ongoing opioid therapy.
- If patients already receiving long-term opioid therapy require additional opioids for superimposed severe acute pain (e.g., major surgery), opioids should be continued only for the duration of pain severe enough to require additional opioids, returning to the patient’s baseline opioid dosage as soon as possible, including a taper to baseline dosage if additional opioids were used around the clock for more than a few days.
- If opioids are used continuously (around the clock) for more than a few days for acute pain, clinicians should prescribe a brief taper to minimize withdrawal symptoms on discontinuation of opioids.
- If a taper is needed, taper durations might need to be adjusted depending on the duration of the initial opioid prescription (see Supporting Rationale for this recommendation for additional details).
- Tapering plans should be discussed with the patient before hospital discharge and with clinicians coordinating the patient’s care as an outpatient. (See Recommendation 5 for tapering considerations when patients have taken opioids continuously for >1 month.)

##### Supporting Rationale

Data suggest that pain improves within days for many patients with common types of acute pain in primary care or emergency department settings. Analysis of nationwide U.S. commercial insurance claims in 2014 found median durations of initial opioid analgesic prescriptions for acute pain indications in primary care settings were 4–7 days (*244*), suggesting that in most cases, clinicians considered an initial opioid prescription of 4–7 days’ duration sufficient. Some patients (17.8%; range: 11.7%–30.0% depending on the acute pain condition) obtained at least one refill within 30 days after their initial opioid prescription, suggesting that although these durations might have been sufficient or more than necessary for most patients, variation across diagnoses and among patients in time to recovery is likely. In an older study of the course of acute low back pain (not associated with malignancies, infections, spondyloarthropathies, fractures, or neurologic signs) in a primary care setting, a large decrease in pain occurred until the fourth day after treatment with paracetamol, with smaller decreases thereafter (*245*). A more recent single-center survey of patients prescribed opioids for acute pain on emergency department discharge (*246*) found that patients taking opioids continued them for a median of 4 days (IQR: 2–7 days), including on the day of discharge, with variation across patients and diagnoses. Median numbers of days that patients continued taking prescribed opioids were 6 days (IQR: 4–8 days) for back pain and fractures, 2 days (IQR: 1–5 days) for renal colic, 5.5 days (IQR: 4–7 days) for musculoskeletal injury, and 3 days (IQR: 2–6) for other diagnoses. Most patients (92.5%) reported having leftover pills, with 52.2% of pills unused overall. A Canadian study following patients for 14 days after discharge from the emergency department with opioid prescriptions for acute pain similarly found most (68%) total prescribed opioids were unused, and the quantity of 5-mg morphine tablets to prescribe to adequately supply 80% of the patients with the amount of opioids they used was 20 tablets for musculoskeletal pain, 30 for fracture, 15 for renal colic or abdominal pain, and 20 for other pain conditions (*247*).

Since 2017, multiple studies have found that many patients do not use all prescribed opioids after surgery and that prescribing a lower quantity of opioids postoperatively is associated with less opioid use without increases in pain score or in requests for refills of pain medication and without reductions in satisfaction with pain management (*77*–*79*). One study found that, after five common surgical procedures, median opioid consumption was three 5-mg oxycodone pills or less, and that following consensus recommendations intended to reduce unnecessary postoperative opioid prescribing published in 2018 and 2019 would still result in 47%–56% of pills prescribed remaining unused (*248*). Evidence exists of variation in opioid needs across patients undergoing the same procedures attributable to factors including pain at discharge and previous opioid use (*249*). One study found that, although a majority of patients used no or few (>0 to <50 MME during their entire postoperative course) opioids, some patients required opioids for up to 15 days after surgery (*250*).

Clinical evidence reviews found observational evidence that opioid use for acute pain is associated with long-term opioid use and that a greater amount of early opioid exposure is associated with greater likelihood of long-term use, noting recent evidence for a dose- and duration-dependent effects (*63*,*75*,*141*,*244*,*251*,*252*). Opioids prescribed for surgery and other acute pain conditions that go unused are a potential source for misuse and diversion (*249*,*253*–*255*). In addition, sudden discontinuation of opioids might result in clinically significant opioid withdrawal (*71*). Therefore, limiting duration of opioids prescribed can minimize the need for a taper to prevent distressing or unpleasant withdrawal symptoms.

Many common causes of nonsurgical, nontraumatic acute pain can often be managed without opioids (see Recommendation 1). When the diagnosis and severity of acute pain warrant the use of opioids, clinicians should prescribe no greater quantity than needed for the expected duration of pain severe enough to require opioids. A few days or less are often sufficient when opioids are needed for many common causes of nonsurgical acute pain, and limiting the duration of opioid therapy can minimize the need to taper to prevent withdrawal symptoms at the end of the course of opioids and limit unused opioids. Certain circumstances (e.g., severe traumatic injuries) might require use of opioids for durations of >7 days. Durations should be individualized based on the patient’s clinical circumstances.

When patients are discharged from the hospital after surgery, the course and dosage of any opioid medications administered during hospitalization and before discharge can help predict ongoing pain management needs (*150*,*256*,*257*). For postoperative pain, procedure-specific opioid prescribing recommendations are available with ranges for amounts of opioids needed (on the basis of use and refills and on consensus) (*149*,*151*,*250*).

Clinicians should generally not prescribe additional opioids to patients just in case pain continues longer than expected. However, if pain continues longer than expected, some patients might face challenges in successfully navigating the health care system (e.g., clinician and pharmacy contact, transportation, and need for assistance) to obtain additional medication as needed, leading to potential disparities in treatment. Clinicians, practices, and health systems should have mechanisms in place for the subset of patients who experience severe acute pain that continues longer than the expected duration. These mechanisms should allow for timely reevaluation to confirm or revise the initial diagnosis and adjust pain management accordingly. In particular, clinicians, practices, and health systems should ensure all patients can obtain and afford additional evaluation and treatment as needed to minimize disparities in access to and affordability of care and refills.

Patients should be evaluated at least every 2 weeks if they continue to receive opioids for acute pain. If opioids are continued for ≥1 month, clinicians should ensure that potentially reversible causes of chronic pain are addressed and that opioid prescribing for acute pain does not unintentionally become long-term opioid therapy simply because medications are continued without reassessment. Continuation of opioid therapy at this point might represent initiation of long-term opioid therapy, which should occur only as an intentional decision that benefits are likely to outweigh risks after discussion between the clinician and patient and as part of a comprehensive pain management approach. Clinicians should refer to recommendations on subacute and chronic pain for initiation (Recommendation 2), follow-up (Recommendation 7), and tapering (Recommendation 5) of ongoing opioid therapy. If patients already receiving long-term opioids require additional opioids for superimposed severe acute pain (e.g., major surgery), opioids should be continued only for the duration of pain severe enough to require additional opioids, returning to the patient’s baseline opioid dosage as soon as possible, including a taper to baseline dosage if additional opioids were used around the clock for more than a few days.

If opioids are used continuously (around the clock) for more than a few days for acute pain, clinicians should prescribe a brief taper to minimize withdrawal symptoms on discontinuation of opioids. Taper durations might need to be adjusted depending on the duration of the initial opioid prescription. For example, if opioids are used continuously for >3 days but for <1 week, clinicians can consider reducing the daily dosage to 50% for 2 days to ameliorate withdrawal symptoms when discontinuing opioids. When patients have taken opioids continuously for ≥1 week but <1 month, clinicians might consider a slower taper (e.g., reducing the daily dosage by approximately 20% every 2 days, a range consistent with tapering rates successfully used in studies of postoperative opioid prescribing) (*256*,*257*). When patients are discharged from the hospital after surgery, opioid dosages needed during hospitalization and before discharge can help predict tapering needs to prevent withdrawal symptoms (*150*,*256*,*257*). Tapering plans should be discussed with the patient before discharge and with clinicians coordinating the patient’s care as an outpatient. (See Recommendation 5 for tapering considerations when patients have taken opioids continuously for >1 month.)

#### Recommendation 7

**Clinicians should evaluate benefits and risks with patients within 1–4 weeks of starting opioid therapy for subacute or chronic pain or of dosage escalation. Clinicians should regularly reevaluate benefits and risks of continued opioid therapy with patients (recommendation category: A; evidence type: 4).**

##### Implementation Considerations

- In addition to evaluating benefits and risks of opioids before starting opioid therapy (see Recommendation 2), clinicians should evaluate patients to assess benefits and risks of opioids within 1–4 weeks of starting long-term opioid therapy or of dosage escalation.
- Clinicians should consider follow-up intervals within the lower end of this range when ER/LA opioids are started or increased, because of the increased risk for overdose within the first 2 weeks of treatment, or when total daily opioid dosage is ≥50 MME/day. (Overdose risk is doubled across multiple studies for dosages of 50 to <100 MME/day relative to <20 MME/day.) (See Recommendation 4.)
- Shorter follow-up intervals (every 2–3 days for the first week) should be strongly considered when starting or increasing the dosage of methadone, because of the variable half-life of this drug (see Recommendation 3) and the potential for drug accumulation during initiation and during upward titration of dosage.
- An initial follow-up interval closer to 4 weeks can be considered when starting immediate-release opioids at a dosage of <50 MME/day.
- Clinicians should follow up with and evaluate patients with subacute pain who started opioid therapy for acute pain and have been treated with opioid therapy for 30 days to reassess the patient’s pain, function, and treatment course; ensure that potentially reversible causes of chronic pain are addressed; and prevent unintentional initiation of long-term opioid therapy. Continuation of opioid therapy at this point might represent initiation of long-term opioid therapy, which should occur only as an intentional decision that benefits are likely to outweigh risks after discussion between the clinician and patient and as part of a comprehensive pain management approach (see Recommendation 2).
- Clinicians should regularly reassess all patients receiving long-term opioid therapy, including patients who are new to the clinician but on long-term opioid therapy, with a suggested interval of every 3 months or more frequently for most patients.
- Clinicians seeing new patients already receiving opioids should establish treatment goals, including functional goals, for continued opioid therapy (see Recommendation 2).
- Clinicians should reevaluate patients who are at higher risk for opioid use disorder or overdose (e.g., patients with depression or other mental health conditions, a history of substance use disorder, a history of overdose, taking ≥50 MME/day, or taking other central nervous system depressants with opioids) more frequently than every 3 months. Clinicians should regularly screen all patients for these conditions, which can change during the course of treatment (see Recommendation 8).
- Clinicians, practices, and health systems can help minimize unintended effects on patients by ensuring all patients can access and afford follow-up evaluation.
- In practice contexts where virtual visits are part of standard care (e.g., in remote areas where distance or other context makes follow-up visits challenging), or for patients for whom in-person follow-up visits are challenging (e.g., frail patients), follow-up assessments that allow the clinician to communicate with and observe the patient through telehealth modalities might be conducted.
- At follow-up, clinicians should review patient perspectives and goals, determine whether opioids continue to meet treatment goals, including sustained improvement in pain and function, and determine whether the patient has experienced common or serious adverse events or early warning signs of serious adverse events or has signs of opioid use disorder.
- Clinicians should ensure that treatment for depression, anxiety, or other psychological comorbidities is optimized.
- Clinicians should ask patients about their preferences for continuing opioids, considering their effects on pain and function relative to any adverse effects experienced. If risks outweigh benefits of continued opioid therapy (e.g., if patients do not experience meaningful, sustained improvements in pain and function compared with before initiation of opioid therapy; if patients are taking higher-risk regimens [e.g., dosages of ≥50 MME/day or opioids combined with benzodiazepines] without evidence of benefit; if patients believe benefits no longer outweigh risks; if patients request dosage reduction or discontinuation; or if patients experience overdose or other serious adverse events), clinicians should work with patients to taper and reduce opioid dosage or taper and discontinue opioids when possible (see from Recommendation 5).
- Clinicians should maximize pain treatment with nonpharmacologic and nonopioid pharmacologic treatments as appropriate (see Recommendation 2).

##### Supporting Rationale

Although clinical evidence reviews did not find studies evaluating the effectiveness of more frequent monitoring intervals (*7*), they identified an observational study (*54*) that found risk for opioid use disorder was associated with continuing opioid therapy for ≥3 months. The reviews also identified a study that found risk for overdose associated with ER/LA opioids might be particularly high during the first 2 weeks of treatment (*192*). Another study found the first 3 months after opioid initiation to be a period of higher risk for opioid overdose (*214*). Patients who do not have pain relief with opioids at 1 month are unlikely to experience pain relief with opioids at 6 months (*258*). Although evidence is insufficient to determine at what point within the first 3 months of opioid therapy the risks for opioid use disorder increase, reassessment of pain and function within 1 month of initiating opioids provides an opportunity to modify the treatment plan to achieve pain treatment goals, including functional goals, and minimize risks of long-term opioid use by tapering and discontinuing opioids among patients not receiving a clear benefit from these medications. In addition, evaluation within the first 3 months might provide opportunities to identify and mitigate risks for opioid use disorder and overdose.

Experts from OWG noted that although little evidence exists for specific follow-up time frames, the recommendation was reasonable and reflects common practice and therefore supported the recommendation. Experts further noted that social determinants of health affecting ability to return frequently for care (e.g., role as unpaid caregiver or work at a job with minimal paid time off) or payer issues (e.g., copays) could have consequences when recommending frequent visits and should be considered.

Clinicians should evaluate patients to assess benefits and risks of opioids within 1–4 weeks of starting long-term opioid therapy or of dosage escalation. Clinicians should consider follow-up intervals within the lower end of this range when ER/LA opioids are started or increased, because of the increased risk for overdose within the first 2 weeks of treatment (*192*), or when total daily opioid dosage is ≥50 MME/day, because the overdose risk is doubled across multiple studies for dosages of 50 to <100 MME/day relative to <20 MME/day (see Recommendation 4). Shorter follow-up intervals (every 2–3 days for the first week) should be strongly considered when starting or increasing the dosage of methadone because of the variable half-life of this drug (see Recommendation 3) and the potential for drug accumulation during initiation and during upward titration of dosage. An initial follow-up interval closer to 4 weeks can be considered when starting immediate-release opioids at a dosage of <50 MME/day.

Patients who started opioid therapy for acute pain and are continuing to receive opioids for subacute pain might be at a particularly critical point for potential transition to chronic pain and potential transition to long-term opioid therapy. Clinicians should follow up with and evaluate patients with subacute pain who have been treated with opioid therapy for 30 days. Clinicians should ensure that opioid prescribing for acute pain does not unintentionally become long-term opioid therapy simply because medications are continued without reassessment, but only as an intentional decision that benefits are likely to outweigh risks after discussion between the clinician and patient. Clinicians should reassess the patient’s pain, function, and treatment course; ensure that potentially reversible causes of chronic pain are addressed; and optimize pain management as needed (see Recommendation 2).

In analyses of placebo-controlled trials, the clinical evidence reviews found that effects of opioids on mean improvement in pain and in function were greater at 1–3 months than at 3–6 months (*7*). A cohort study found an association between longer duration of therapy and increased risk for new-onset depression (*7*). Because of potential changes in the balance of benefits and risks of opioid therapy over time, clinicians should regularly reassess all patients receiving long-term opioid therapy, including patients who are new to the clinician but on long-term opioid therapy, with a suggested interval of every 3 months or more frequently. Clinicians seeing new patients already receiving opioids should establish treatment goals, including functional goals, for continued opioid therapy (see Recommendation 2). Clinicians should reevaluate patients who are at greater risk for opioid use disorder or overdose (e.g., patients with depression or other mental health conditions, a history of substance use disorder, a history of overdose, taking ≥50 MME/day, or taking other central nervous system depressants with opioids) more frequently than every 3 months. Clinicians should regularly screen all patients for these conditions, which can change during the course of treatment (see Recommendation 8). Clinicians, practices, and health systems can help minimize unintended effects on patients by ensuring all patients can access and afford follow-up evaluation (*86*). In addition, policymakers can consider evidence-based methods of minimizing barriers to care (e.g., paid sick leave) (*259*). In practice contexts where virtual visits are part of standard care (e.g., in remote areas where distance or other context makes follow-up visits challenging), or for patients for whom in-person follow-up visits are challenging (e.g., frail patients), follow-up assessments that allow the clinician to communicate with and observe the patient through telehealth modalities might be conducted when available.

At follow-up, clinicians should review patient perspectives on progress and challenges in moving toward treatment goals; determine whether opioids continue to meet treatment goals, including sustained improvement in pain and function; determine whether the patient has experienced common or serious adverse events or early warning signs of serious adverse events or has signs of opioid misuse or opioid use disorder (e.g., difficulty controlling use, cravings, work, and social or family problems related to opioid use); determine whether benefits of opioids continue to outweigh risks; and determine whether there is a need for opioid dosage reduction or discontinuation. Clinicians should assess benefits in function, pain control, and quality of life by asking patients about progress toward person-centered functional goals that have meaning for them (see Recommendation 2) or by using tools such as the three-item PEG assessment scale (*184*); clinically meaningful improvement has been defined as a 30% improvement in scores for both pain and function (*185*). Clinicians also should ask patients about common adverse effects such as constipation and drowsiness (see Recommendation 2) and should ask about and assess for effects that might be early warning signs for more serious problems such as overdose (e.g., sedation or slurred speech) or opioid use disorder (e.g., craving, wanting to take opioids in greater quantities or more frequently than prescribed, difficulty controlling use, or work, social, or family problems related to opioid use). Clinicians can use validated screening tools such as the Drug Abuse Screening Test (DAST) (*260*), the Tobacco, Alcohol, Prescription medication, and other Substance use Tool (TAPS) (*261*), and the three-question version of the Alcohol Use Disorders Identification Test (AUDIT-C) (*262*,*263*) (see Recommendations 8 and 12). Because depression, anxiety, and other psychological comorbidities often coexist with and can interfere with resolution of pain, clinicians should use validated instruments to assess for these conditions (see Recommendation 8) and ensure that treatment for these conditions is optimized. Clinicians should ask patients about their preferences for continuing opioids considering their effects on pain and function relative to any adverse effects experienced.

If risks outweigh benefits of continued opioid therapy (e.g., if patients do not experience meaningful, sustained improvements in pain and function compared with before initiation of opioid therapy; if patients are taking higher-risk regimens [e.g., dosages of ≥50 MME/day or opioids combined with benzodiazepines] without evidence of benefit; if patients believe benefits no longer outweigh risks; if patients request dosage reduction or discontinuation; or if patients experience overdose or other serious adverse events), clinicians should work with patients to taper and reduce opioid dosage or to taper and discontinue opioids when possible (see Recommendation 5). Clinicians should maximize pain treatment with nonpharmacologic and nonopioid pharmacologic treatments as appropriate (see Recommendation 2).

### Assessing Risk and Addressing Potential Harms of Opioid Use

#### Recommendation 8

**Before starting and periodically during continuation of opioid therapy, clinicians should evaluate risk for opioid-related harms and discuss risk with patients. Clinicians should work with patients to incorporate into the management plan strategies to mitigate risk, including offering naloxone (recommendation category: A; evidence type: 4).**

##### Implementation Considerations

- Clinicians should ask patients about their drug and alcohol use and use validated tools or consult with behavioral specialists to screen for and assess mental health and substance use disorders.
- When considering initiating long-term opioid therapy, clinicians should ensure that treatment for depression and other mental health conditions is optimized, consulting with behavioral health specialists when needed.
- Clinicians should offer naloxone when prescribing opioids, particularly to patients at increased risk for overdose, including patients with a history of overdose, patients with a history of substance use disorder, patients with sleep-disordered breathing, patients taking higher dosages of opioids (e.g., ≥50 MME/day), patients taking benzodiazepines with opioids (see Recommendation 11), and patients at risk for returning to a high dose to which they have lost tolerance (e.g., patients undergoing tapering or recently released from prison).
- Practices should educate patients on overdose prevention and naloxone use and offer to provide education to members of their households.
- Naloxone coprescribing can be facilitated by clinics or practices with resources to provide naloxone training, by collaborative practice models with pharmacists, or through statewide protocols or standing orders for naloxone at pharmacies.
- Resources for prescribing naloxone in primary care and emergency department settings can be found through Prescribe to Prevent at https://prescribetoprevent.org. Additional resources are at https://www.samhsa.gov.
- In part because of concerns about cost of naloxone and access for some patients and reports that purchasing of naloxone has in some cases been required to fill opioid prescriptions, including for patients without a way to afford naloxone, this recommendation specifies that naloxone should be offered to patients. To that end, clinicians, health systems, and payers can work to ensure patients can obtain naloxone, a potentially lifesaving treatment.
- Clinicians should avoid prescribing opioids to patients with moderate or severe sleep-disordered breathing when possible to minimize risk for respiratory depression.
- When making decisions about whether to initiate opioid therapy for pain during pregnancy, clinicians and patients together should carefully weigh benefits and risks. For pregnant persons already receiving opioids, clinicians should access appropriate expertise if tapering is being considered because of possible risks to the pregnant patient and the fetus if the patient goes into withdrawal (see Recommendation 5).
- For pregnant persons with opioid use disorder, medication for opioid use disorder (buprenorphine or methadone) is the recommended therapy and should be offered as early as possible in pregnancy to prevent harms to both the patient and the fetus (see Recommendation 12).
- Clinicians should use additional caution and increased monitoring (see Recommendation 7) to minimize risks of opioids prescribed for patients with renal or hepatic insufficiency and for patients aged ≥65 years. Clinicians should implement interventions to mitigate common risks of opioid therapy among older adults, such as exercise or bowel regimens to prevent constipation, risk assessment for falls, and patient monitoring for cognitive impairment.
- For patients with jobs that involve potentially hazardous tasks and who are receiving opioids or other medications that can negatively affect sleep, cognition, balance, or coordination, clinicians should assess patients’ abilities to safely perform the potentially hazardous tasks (e.g., driving, use of heavy equipment, climbing ladders, working at heights or around moving machinery, or working with high-voltage equipment).
- Clinicians should use PDMP data (see Recommendation 9) and toxicology screening (see Recommendation 10) as appropriate to assess for concurrent substance use that might place patients at higher risk for opioid use disorder and overdose.
- Clinicians should provide specific counseling on increased risks for overdose when opioids are combined with other drugs or alcohol (see Recommendation 2) and ensure that patients are provided or receive effective treatment for substance use disorders when needed (see Recommendation 12).
- Although substance use disorders can alter the expected benefits and risks of opioid therapy for pain, patients with co-occurring pain and substance use disorder require ongoing pain management that maximizes benefits relative to risks. (See Recommendation 12, Pain Management for Patients with Opioid Use Disorder for additional considerations specific to these patients.)
- If clinicians consider opioid therapy for chronic pain for patients with substance use disorder, they should discuss increased risks for opioid use disorder and overdose with patients, carefully consider whether benefits of opioids outweigh increased risks, and incorporate strategies to mitigate risk into the management plan (e.g., offering naloxone [see Offering Naloxone to Patients] and increasing frequency of monitoring [see Recommendation 7]).
- If patients experience nonfatal opioid overdose, clinicians should evaluate for opioid use disorder and treat or arrange treatment if needed. Clinicians should work with patients to reduce opioid dosage and to discontinue opioids when indicated (see Recommendation 5) and should ensure continued close monitoring and support for patients prescribed or not prescribed opioids.
- If clinicians continue opioid therapy in patients with previous opioid overdose, they should discuss increased risks for overdose with patients, carefully consider whether benefits of opioids outweigh substantial risks, and incorporate strategies to mitigate risk into the management plan (e.g., offering naloxone and increasing frequency of monitoring [see Recommendation 7]).

##### Supporting Rationale

The clinical evidence reviews found evidence too limited to determine effects of patient demographics and comorbidities on risk for opioid-related harms (*7*). However, on the basis of observational studies (*181*,*264*–*273*) and expert opinion, certain risk factors are likely to increase susceptibility to opioid-related harms and warrant incorporation of additional strategies into the management plan to mitigate risk. Clinicians should assess these risk factors periodically, with frequency individualized to patient comorbidities and other risk factors. For example, factors that vary over time, such as alcohol use, require more frequent assessment. Clinicians should offer naloxone and reevaluate patients more frequently (see Recommendation 7) when factors are present that increase risk for harm, such as sleep-disordered breathing, history of overdose, history of substance use disorder, higher dosages of opioids (e.g., ≥50 MME/day), and concurrent use of benzodiazepines with opioids. Experts from OWG had concerns about the cost of purchasing naloxone for patients with limited means and reported that purchasing of naloxone has in some cases been required to fill opioid prescriptions. In part because of these concerns and because in certain settings naloxone is directly provided by a practice or health system to patients, “offering” naloxone (which can be done by offering a prescription or by offering naloxone directly) is recommended rather than specifying “prescribing” naloxone. Clinicians, health systems, and payers should work to ensure patients can obtain naloxone, a potentially lifesaving treatment.

#### Patients with Sleep-Disordered Breathing, Including Sleep Apnea

A case-control analysis among veterans prescribed opioids found that sleep apnea was associated with increased risk for life-threatening respiratory/central nervous system depression or overdose (*264*). Careful monitoring and cautious dose titration should be used if opioids are prescribed for patients with mild sleep-disordered breathing. Clinicians should avoid prescribing opioids to patients with moderate or severe sleep-disordered breathing, whenever possible, to minimize risks for respiratory depression.

#### Pregnant Persons

Pregnant, postpartum, and parenting persons should receive compassionate, evidence-based care for pain or opioid use disorder. ACOG has noted that a cautious approach to prescribing opioids should be balanced with the need to address pain, and pregnancy should not be a reason to avoid treating acute pain (*274*). At the same time, opioid use during pregnancy might be associated with risks to both the pregnant person and the fetus. Certain observational studies have shown an association of opioid use in pregnancy with stillbirth, poor fetal growth, and preterm delivery (*265*–*268*,*275*). In some cases, opioid use during pregnancy leads to neonatal abstinence syndrome/neonatal opioid withdrawal syndrome (*269*). ACOG has emphasized that pregnancy should not be a reason to avoid treating acute pain because of concern for opioid misuse or neonatal abstinence syndrome and that neonatal abstinence syndrome is an expected and treatable condition that can follow prenatal exposure to opioid agonists.

Clinicians and patients together should carefully weigh benefits and risks when making decisions about whether to initiate opioid therapy for pain during pregnancy. In addition, before initiating opioid therapy for persons who can become pregnant, clinicians and patients should discuss family planning and potential effects of long-term opioid use on any future pregnancy. For all persons with reproductive potential, discussing future pregnancy intentions and engaging in shared decision-making regarding contraception, if appropriate, is a core component of care. A review of all prescription and nonprescription medications is recommended during prepregnancy and interpregnancy care (*276*,*277*). Intentional application of a patient-centered reproductive justice framework and use of a shared decision-making model is the recommended approach for providing supportive contraceptive counseling and care to help patients to achieve their reproductive goals (*278*). Counseling should be noncoercive and include a discussion of all contraceptive options (*276*–*278*). When opioids are needed for treatment of acute pain in pregnant persons, the lowest effective dose (see Recommendation 4) should be used for no longer than the expected duration of pain severe enough to require opioids (see Recommendation 6). For pregnant persons with chronic pain, ACOG recommends that practice goals include strategies to avoid or minimize the use of opioids for pain management, highlighting alternative pain therapies such as nonpharmacologic (e.g., exercise, physical therapy, and behavioral approaches), and nonopioid pharmacologic treatments (*274*). Pharmacokinetic and physiologic changes occur during pregnancy, especially in the third trimester, and these changes might require dose adjustments (*274*). For pregnant persons already receiving opioids, clinicians should access appropriate expertise if considering tapering opioids because of possible risk to the pregnant patient and the fetus if the patient goes into withdrawal (see Recommendation 5).

ACOG has noted that early universal screening, brief intervention (e.g., engaging in a short conversation and providing feedback and advice), and referral for treatment of pregnant persons with opioid use disorder improve both maternal and infant outcomes (*274*). For pregnant persons with opioid use disorder, medication for opioid use disorder (buprenorphine or methadone) is the recommended therapy, has been associated with improved maternal outcomes, and should be offered as early as possible in pregnancy to prevent harms to both the patient and the fetus (*274*) (see Recommendation 12). In contrast, criminalization or otherwise punishing (e.g., through threatened loss of child custody) the use of opioids, including for opioid use disorder, discourages pregnant, postpartum, and parenting persons from seeking care; nonpunitive public health approaches to treatment result in better outcomes (*274*,*279*).

The American Academy of Pediatrics (AAP) has published recommendations for the care of infants with neonatal opioid withdrawal syndrome, including that pregnant persons with opioid use disorder should receive antenatal counseling to provide education on the clinical signs of withdrawal and on postnatal treatment for neonatal opioid withdrawal syndrome (e.g., nonpharmacologic treatment, including breastfeeding, and pharmacotherapy) (*280*). In addition, all infants with long-term opioid exposure should be observed for at least 72 hours (4–7 days if exposed to buprenorphine or ER/LA opioids and 5–7 days if exposed to methadone) to monitor for the development of withdrawal (*280*). Clinicians caring for pregnant persons receiving prescribed or using nonprescribed opioids should arrange for delivery at a facility prepared to monitor, evaluate for, and treat neonatal opioid withdrawal syndrome. In instances when travel to such a facility would present an undue burden on the pregnant person, it is appropriate for the clinician to arrange delivery locally, monitor and evaluate the newborn for neonatal opioid withdrawal syndrome, and transfer the newborn for additional treatment if needed. Previous consensus recommendations have advised that if a codeine-containing medication is selected for postpartum management, clinicians should review duration of therapy and neonatal signs of toxicity with patients and their families (*133*).

#### Patients with Renal or Hepatic Insufficiency

A case-control study of risk for life-threatening respiratory/central nervous system depression or overdose among veterans prescribed opioids found that renal disease and moderate or severe liver disease were associated with increased risk for these events (*264*). Clinicians should use additional caution and increased monitoring (see Recommendation 7) to minimize risks of opioids prescribed for patients with renal or hepatic insufficiency because of their decreased ability to process and excrete medications, susceptibility to accumulation of opioids, and reduced therapeutic window between safe dosages and dosages associated with respiratory depression and overdose (*281*) (see Recommendations 3, 4, and 7).

#### Patients Aged ≥65 Years

Older adults are a heterogenous group comprising a wide span of ages and functional abilities, ranging from healthy, active older adults to frail older adults. Frail older adults in particular can be at risk for changes in function that might be exacerbated by pain and contribute to deterioration in overall health and independence. Functional assessment is especially important in patients aged ≥65 years to better assess effects of pain on function and independence. Persons aged ≥65 years can be at risk for inadequate pain treatment (*2*,*6*,*17*,*282*). For certain older adults (e.g., older adults with serious illness that requires advanced management of pain or other distressing symptoms) (*94*), palliative care, which is beyond the scope of this guideline but addressed in other guidelines (*93*), is appropriate.

Pain management for older patients can be challenging because of increased risks of both nonopioid pharmacologic therapies (see Recommendation 2) and opioid therapy in this population. Because of reduced renal function and medication clearance even in the absence of renal disease, patients aged ≥65 years might have increased susceptibility to accumulation of all medications, increased risk for drug-drug interactions, and a smaller therapeutic window between safe dosages and dosages associated with adverse effects. These adverse effects include renal, cardiovascular, and gastrointestinal effects with oral NSAIDs (see Recommendation 2) and respiratory depression and overdose with opioids. A case-control analysis among veterans prescribed opioids found that age ≥55 years was associated with increased risk for life-threatening respiratory/central nervous system depression or overdose (*264*). Some older adults might have a cognitive impairment, such as dementia, that can increase risk for medication errors and make opioid-related confusion riskier. In addition, older adults are more likely than younger adults to experience comorbid medical conditions and are more likely to receive multiple medications, some of which might interact with opioids.

Clinicians should review all current medications, over-the-counter drugs, and natural remedies before prescribing any new drugs. Clinicians should use additional caution and increased monitoring (see Recommendation 7) for patients aged ≥65 years to ensure pain is addressed and minimize risks of opioids prescribed. Clinicians should educate older adults receiving opioids to avoid medication-related behaviors that increase risk, such as saving unused medications. Caregivers can have an important role in management of opioid therapy for older persons with cognitive impairment. Clinicians also should implement interventions to mitigate common risks of opioid therapy among older adults, such as monitoring for cognitive impairment, risk assessment for falls, and exercise and bowel regimens to prevent constipation.

#### Patients in Safety Critical Jobs

A safety critical job involves work or an occupational environment where limitations in physical or mental performance, or both, involve dangers to self, coworkers, or the public. According to the American College Occupational Environmental Medicine, for occupations with higher risks (especially public transportation), prescription of an opioid might be incompatible with continued employment in a safety critical job (*270*,*283*). For patients with safety critical jobs who are receiving opioids or other medications that can negatively affect sleep, cognition, balance, or coordination, clinicians should assess patients’ abilities to perform jobs that involve driving, using heavy equipment, climbing ladders, working at heights or around moving machinery, or working with high-voltage equipment.

#### Patients with Mental Health Conditions

Psychological distress frequently interferes with improvement of pain and function in patients with chronic pain; therefore, using validated instruments such as the Generalized Anxiety Disorder (GAD)-7 and the Patient Health Questionnaire (PHQ-9 or PHQ-4) to support assessment for anxiety, posttraumatic stress disorder (PTSD), and depression (*284*) might help clinicians improve overall pain treatment outcomes. Patients with mental health conditions including depression might be at higher risk than other patients for opioid use disorder (*181*,*271*) and drug overdose (*272*). Additional caution and increased monitoring (see Recommendation 7) might lessen the increased risk for overdose among patients with depression (*264*,*272*). In addition, patients with anxiety disorders and other mental health conditions are more likely to receive benzodiazepines, which can exacerbate opioid-induced respiratory depression and increase risk for overdose (see Recommendation 11). Clinicians should ensure that treatment for depression and other mental health conditions as well as treatment for pain is optimized, consulting with behavioral health specialists when needed. Treatment for depression can improve pain symptoms and depression and might decrease overdose risk (*272*). For treatment of chronic pain in patients with depression, clinicians should consider using tricyclic or SNRI antidepressants for analgesic as well as antidepressant effects if these medications are not otherwise contraindicated (see Recommendation 2).

#### Patients with Substance Use Disorders

Patients with substance use disorders are likely to experience greater risks for opioid use disorder and overdose (*55*,*202*,*264*) than persons without these conditions. Despite increased risk for opioid misuse and opioid use disorder when prescribed opioid analgesics (*271*,*285*), patients with histories of substance use disorders are more likely than other patients to receive long-term opioid treatment for chronic pain (*286*). Previous guidelines have recommended screening or risk assessment tools to identify patients at higher risk for opioid misuse or opioid use disorder. However, the clinical evidence reviews found that available risk stratification tools (e.g., Opioid Risk Tool, Screener and Opioid Assessment for Patients with Pain [SOAPP] Version 1, SOAPP-R, and Brief Risk Interview) demonstrate limited and variable accuracy for classification of patients as at low or high risk for opioid use disorder or misuse (*7*). If these tools are used, they should be supplemented with other assessments, such as discussions with patients, family, and caregivers; clinical records; PDMP data (see Recommendation 9); and toxicology screening data (see Recommendation 10). Clinicians should always use caution when considering or prescribing opioids and should not overestimate the ability of available risk stratification tools to rule out risks of long-term opioid therapy.

Nonprescribed drugs (e.g., heroin, illicitly manufactured fentanyl, cocaine, and methamphetamine) (*287*) and alcohol (*288*) are listed as contributory factors on a substantial proportion of death certificates for prescription opioid–involved overdose deaths. Clinicians should ask patients about their drug (*289*) and alcohol use. Single screening questions can be used (*290*). For example, the question “How many times in the past year have you used an illegal drug or used a prescription medication for nonmedical reasons?” (with an answer of one or more considered positive) was found in a primary care setting to be 100% sensitive and 73.5% specific for the detection of a drug use disorder compared with a standardized diagnostic interview (*291*). Validated screening tools, such as the Drug Abuse Screening Test (DAST) (*260*); the Tobacco, Alcohol, Prescription medication, and other Substance use Tool (TAPS) (*261*); and the three-question version of the Alcohol Use Disorders Identification Test (AUDIT-C) (*262*,*263*), also can be used. Clinicians should use PDMP data (see Recommendation 9) and toxicology screening (see Recommendation 10) as appropriate to assess for concurrent substance use that might place patients at higher risk for opioid use disorder and overdose. Clinicians should also provide specific counseling on increased risks for overdose when opioids are combined with other drugs or alcohol (see Recommendation 2) and ensure that patients receive effective treatment for substance use disorders when needed (see Recommendation 12).

If clinicians consider prescribing opioid therapy for chronic pain to patients with substance use disorders, they should discuss increased risks for opioid use disorder and overdose with patients; carefully consider whether benefits of opioids outweigh increased risks; and incorporate strategies to mitigate risk into the management plan, such as offering naloxone (see Offering Naloxone to Patients) and increasing frequency of monitoring (see Recommendation 7) when opioids are prescribed. Clinicians should communicate with patients’ substance use disorder treatment providers if opioids are prescribed. Although substance use disorders can alter the expected benefits and risks of opioid therapy for pain, patients with co-occurring pain and substance use disorder require ongoing pain management that maximizes benefits relative to risks. (See Recommendation 12, Pain Management for Patients with Opioid Use Disorder for additional considerations.)

#### Patients with Previous Overdose

Previous opioid overdose is associated with substantially increased risk for future nonfatal or fatal opioid overdose (*273*). Yet, a cohort study of commercially insured patients found that opioids were dispensed to 91% of patients who had a previous overdose; a substantial percentage experienced a repeated opioid overdose, with a cumulative incidence at 2 years of 17% among patients receiving ≥100 MME/day, 15% among those prescribed 50–100 MME/day, 9% among those prescribed <50 MME/day, and 8% among those prescribed no opioids (*273*).

If patients experience nonfatal opioid overdose, clinicians should evaluate them for opioid use disorder and provide or arrange treatment if needed. Treatment with buprenorphine or methadone for opioid use disorder after overdose is associated with reduced all-cause and opioid-related deaths (*292*). Clinicians should work with patients to reduce opioid dosage and discontinue opioids when indicated (see Recommendation 5) and should ensure continued close monitoring and support for patients prescribed or not prescribed opioids. If clinicians continue opioid therapy in patients with previous opioid overdose, they should discuss increased risks for overdose with patients; carefully consider whether benefits of opioids outweigh substantial risks; and incorporate strategies to mitigate risk into the management plan, such as offering naloxone (see Offering Naloxone to Patients), involving patient-identified trusted family members, and increasing frequency of monitoring combined with shorter prescription durations (see Recommendation 7).

#### Offering Naloxone to Patients

Naloxone is an opioid antagonist that can reverse severe respiratory depression; its administration by laypersons, such as friends, family, and caregivers of persons who experience opioid overdose, can save lives (*293*). Naloxone precipitates acute withdrawal among patients physically dependent on opioids. Serious adverse effects (e.g., pulmonary edema, cardiovascular instability, and seizures) have been reported but are rare at doses consistent with labeled use for opioid overdose (*294*). The clinical evidence reviews identified one observational study (*295*) that found provision of naloxone to patients prescribed opioids in primary care clinics was associated with decreased likelihood of opioid-related emergency department visits (*7*).

Clinicians should offer naloxone when prescribing opioids, particularly to patients at increased risk for overdose, including patients with a history of overdose, patients with a history of substance use disorder, patients taking benzodiazepines with opioids (see Recommendation 11), patients at risk for returning to a high dose to which they have lost tolerance (e.g., patients undergoing tapering or recently released from prison), and patients taking higher dosages of opioids (≥50 MME/day). Practices should provide education on overdose prevention and naloxone use to patients receiving naloxone prescriptions and members of their households. Naloxone coprescribing can be facilitated by clinics or practices with resources to provide naloxone training and by collaborative practice models with pharmacists. Resources for prescribing naloxone in primary care settings can be found through Prescribe to Prevent at https://prescribetoprevent.org.

#### Recommendation 9

**When prescribing initial opioid therapy for acute, subacute, or chronic pain, and periodically during opioid therapy for chronic pain, clinicians should review the patient’s history of controlled substance prescriptions using state prescription drug monitoring program (PDMP) data to determine whether the patient is receiving opioid dosages or combinations that put the patient at high risk for overdose (recommendation category: B; evidence type: 4).**

##### Implementation Considerations

- Ideally, PDMP data should be reviewed before every opioid prescription for acute, subacute, or chronic pain. This practice is recommended in all jurisdictions where PDMP availability and access policies, as well as clinical practice settings, make it practicable (e.g., clinician and delegate access permitted).
- At a minimum, during long-term opioid therapy, PDMP data should be reviewed before an initial opioid prescription and then every 3 months or more frequently. Recommendation category B acknowledges variation in PDMP availability and circumstances. However, because PDMP information can be most helpful when results are unexpected and, to minimize bias in application, clinicians should apply this recommendation when feasible to all patients rather than differentially on the basis of assumptions about what they will learn about specific patients.
- Clinicians should use specific PDMP information about medications prescribed to their patient in the context of other clinical information, including their patient’s history, physical findings, and other relevant testing, to help them communicate with and protect their patient.
- Clinicians should review PDMP data specifically for prescription opioids and other controlled medications patients have received from additional prescribers to determine whether a patient is receiving total opioid dosages or combinations (e.g., opioids combined with benzodiazepines) that put the patient at risk for overdose.
- PDMP-generated risk scores have not been validated against clinical outcomes such as overdose and should not take the place of clinical judgment.
- Clinicians should not dismiss patients from their practice on the basis of PDMP information. Doing so can adversely affect patient safety and could result in missed opportunities to provide potentially lifesaving information (e.g., about risks of prescription opioids and about overdose prevention) and interventions (e.g., safer prescriptions, nonopioid pain treatment [see Recommendations 1 and 2], naloxone [see Recommendation 8], and effective treatment for substance use disorders [see Recommendations 8 and 12]).
- Clinicians should take actions to improve patient safety:
  - Discuss information from the PDMP with the patient and confirm that the patient is aware of any additional prescriptions. Because clinicians often work as part of teams, prescriptions might appropriately be written by more than one clinician coordinating the patient’s care. Occasionally, PDMP information can be incorrect (e.g., if the wrong name or birthdate has been entered, the patient uses a nickname or maiden name, or another person has used the patient’s identity to obtain prescriptions).
  - Discuss safety concerns, including increased risk for respiratory depression and overdose, with patients found to be receiving overlapping prescription opioids from multiple clinicians who are not coordinating the patient’s care or patients who are receiving medications that increase risk when combined with opioids (e.g., benzodiazepines) (see Recommendation 11), and offer naloxone (see Recommendation 8).
  - Use particular caution when prescribing opioid pain medication and benzodiazepines concurrently, understanding that some patient circumstances warrant prescribing of these medications concomitantly. Clinicians should communicate with others managing the patient to discuss the patient’s needs, prioritize patient goals, weigh risks of concurrent benzodiazepine and opioid exposure, and coordinate care (see Recommendation 11).
  - Consider the total MME/day for concurrent opioid prescriptions to help assess the patient’s overdose risk (see Recommendation 4). Buprenorphine should not be counted in the total MME/day in calculations because of its partial agonist properties at opioid receptors that confer a ceiling effect on respiratory depression. If a patient is found to be receiving total daily dosages of opioids that put them at risk for overdose, discuss safety concerns with the patient, consider in collaboration with the patient whether or not benefits of tapering outweigh risks of tapering (see Recommendation 5), and offer naloxone (see Recommendation 8).
  - Discuss safety concerns with other clinicians who are prescribing controlled substances for the patient. Ideally, clinicians should first discuss concerns with the patient and inform them that they plan to coordinate care with their other clinicians to improve the patient’s safety.
  - Screen for substance use and discuss concerns with the patient in a nonjudgmental manner (see Recommendations 8 and 12).
  - When diverting (sharing or selling prescription opioids and not taking them) might be likely, consider toxicology testing to assist in determining whether prescription opioids can be discontinued without causing withdrawal (see Recommendations 5 and 10). A negative toxicology test for prescribed opioids might indicate the patient is not taking prescribed opioids, although clinicians should consider other possible reasons for this test result (e.g., false-negative results or misinterpretation of results) (see Recommendation 10).

##### Supporting Rationale

PDMPs are databases overseen by states, territories, counties, and the District of Columbia that collect information on controlled prescription drugs dispensed by pharmacies and, in selected jurisdictions, by dispensing clinicians. PDMPs do not report nonprescribed opioid use. A clinical evidence review did not find studies evaluating the effectiveness of PDMPs for risk mitigation (*7*). However, among patients receiving concurrent treatment with opioids and benzodiazepines, overdose risk is further increased among patients receiving these treatments from multiple prescribers rather than one prescriber, highlighting potential room for improvement in care coordination (*296*). PDMP data also can be helpful when patient medication history is not otherwise available (e.g., when patients transition care to a new clinician). A contextual evidence review (*7*) identified a survey of physicians in Maryland (*297*) finding that although barriers to PDMP review were noted (e.g., not knowing about the program, registration difficulties, and difficulty accessing data), most participants felt that PDMPs improved opioid prescribing by decreasing opioid prescription amounts and increasing comfort with prescribing opioids (*7*). Integration of PDMPs with electronic health records (EHRs) can reduce burden on clinicians compared with having to access a separate system (*298*,*299*).

Special attention should be paid to ensure that PDMP information is not used in a way that is harmful to patients. For example, PDMP information has been used to dismiss patients from clinician practices (*300*), which might adversely affect patient safety and result in untreated or undertreated pain. Many state laws require PDMP use under specific circumstances (*301*). Experts from OWG had concerns about PDMP risk scores or other algorithmic interpretations from software platforms that can lead to distrust between clinicians and patients and stigmatization, particularly for patients with conditions such as opioid use disorder. Risk scores are reportedly generated by applying proprietary algorithms that are not publicly available to information from patient EHRs and other sources such as court records and criminal and sexual trauma histories; these algorithms might disparately affect women, persons of color, and persons who live in poverty (*302*). Importantly, whereas one PDMP-generated risk measure has shown fair concurrence with the WHO Alcohol, Smoking, and Substance Involvement Screening Test (ASSIST), these scores have not been externally validated against clinical outcomes (*302*,*303*). Such risk scores should not take the place of clinical judgment. Rather, clinicians should use specific PDMP information about medications prescribed to their patient in the context of other clinical information, including their patient’s history, physical findings, and other relevant testing, to help them communicate with and protect their patient.

Experts raised varying points regarding frequency of PDMP use, with many agreeing that PDMPs should be consulted before every opioid prescription, several agreeing that universal application would mitigate bias in application to different patients, and others believing it might not be warranted or feasible to check the PDMP in all cases, particularly before prescribing opioids for acute pain for a small number of days. Ideally, PDMP data should be reviewed before every opioid prescription for acute, subacute, or chronic pain. This practice is recommended in all jurisdictions where PDMP availability and access policies make it practicable (e.g., clinician and delegate access permitted). At a minimum, PDMP data should be reviewed before initial opioid prescriptions for subacute or chronic pain and then every 3 months or more frequently during long-term opioid therapy. Recommendation category B acknowledges variation in PDMP availability and circumstances (e.g., a clinician might reasonably determine that a patient with severe acute pain in the emergency department during a PDMP system access failure would be adversely affected by waiting hours for a prescription). However, because PDMP information can be most helpful when results are unexpected and, to minimize bias in application, clinicians should apply this recommendation when feasible to all patients rather than differentially on the basis of assumptions about what they will learn about specific patients.

Clinicians should review PDMP data for prescription opioids and other controlled medications patients might have received from additional prescribers to determine the total amount of MME prescribed and to assess if the total dosage or combinations (e.g., opioids combined with benzodiazepines) put the patient at high risk for overdose. If patients are found to have total opioid dosages or combinations of medications that might put them at risk for overdose, or multiple controlled substance prescriptions written by different clinicians, clinicians should take actions to improve patient safety (see Recommendation 9, Implementation Considerations).

#### Recommendation 10

**When prescribing opioids for subacute or chronic pain, clinicians should consider the benefits and risks of toxicology testing to assess for prescribed medications as well as other prescribed and nonprescribed controlled substances (recommendation category: B; evidence type: 4).**

##### Implementation Considerations

- Toxicology testing should not be used in a punitive manner but should be used in the context of other clinical information to inform and improve patient care. Clinicians should not dismiss patients from care on the basis of a toxicology test result. Dismissal could have adverse consequences for patient safety, potentially including the patient obtaining opioids or other drugs from alternative sources and the clinician missing opportunities to facilitate treatment for substance use disorder.
- Before starting opioids and periodically (at least annually) during opioid therapy, clinicians should consider the benefits and risks of toxicology testing to assess for prescribed opioids and other prescription and nonprescription controlled substances that increase risk for overdose when combined with opioids, including nonprescribed and illicit opioids and benzodiazepines.
- Clinicians, practices, and health systems should aim to minimize bias in testing and should not apply this recommendation differentially on the basis of assumptions about patients.
- Predicting risk is challenging, and available tools do not allow clinicians to reliably identify patients who are at low risk for substance use or substance use disorders. Clinicians should consider toxicology screening results as potentially useful data, in the context of other clinical information, for all patients and consider toxicology screening whenever its potential limitations can be addressed.
- Clinicians should explain to patients that toxicology testing will not be used to dismiss patients from care and is intended to improve their safety.
- Clinicians should explain expected results (e.g., presence of prescribed medication and absence of drugs, including nonprescribed controlled substances not reported by the patient) and ask patients in a nonjudgmental manner about use of prescribed and other drugs and whether there might be unexpected results.
- Limited toxicology screening can be performed with a relatively inexpensive presumptive immunoassay panel that tests for opiates as a class, benzodiazepines as a class, and several nonprescribed substances. Toxicology screening for a class of drugs might not detect all drugs in that class. For example, fentanyl testing is not included in widely used toxicology assays that screen for opiates as a class.
- Clinicians should be familiar with the drugs included in toxicology screening panels used in their practice and should understand how to interpret results for these drugs. For example, a positive opiates immunoassay detects morphine, which might reflect patient use of morphine, codeine, or heroin, but does not detect synthetic opioids and might not detect semisynthetic opioids. In some cases, positive results for specific opioids might reflect metabolites from opioids the patient is taking and might not mean the patient is taking the specific opioid that resulted in the positive test.
- Confirmatory testing should be used when
  - toxicology results will inform decisions with major clinical or nonclinical implications for the patient;
  - a need exists to detect specific opioids or other drugs within a class, such as those that are being prescribed, or those that cannot be identified on standard immunoassays; or
  - a need exists to confirm unexpected screening toxicology test results.
- Restricting confirmatory testing to situations and substances for which results can reasonably be expected to affect patient management can reduce costs of toxicology testing.
- Clinicians might want to discuss unexpected results with the local laboratory or toxicologist and should discuss unexpected results with the patient.
- Clinicians should discuss unexpected results with patients in a nonjudgmental manner, avoiding use of potentially stigmatizing language (e.g., avoid describing a specimen as testing “clean” or “dirty”).
- Discussion with patients before specific confirmatory testing can sometimes yield a candid explanation of why a particular substance is present or absent and remove the need for confirmatory testing during that visit. For example, a patient might explain that the test is negative for prescribed opioids because they felt opioids were no longer helping and discontinued them. If unexpected results from toxicology screening are not explained, a confirmatory test on the same sample using a method selective enough to differentiate specific opioids and metabolites (e.g., gas or liquid chromatography–mass spectrometry) might be warranted.
- Clinicians should use unexpected results to improve patient safety (e.g., optimize pain management strategy [see Recommendation 2], carefully weigh benefits and risks of reducing or continuing opioid dosage [see Recommendation 5], reevaluate more frequently [see Recommendation 7], offer naloxone [see Recommendation 8], and offer treatment or refer the patient for treatment with medications for opioid use disorder [see Recommendation 12], all as appropriate).

##### Supporting Rationale

The clinical evidence reviews did not find studies evaluating the effectiveness of toxicology screening for risk mitigation during opioid prescribing for pain. However, concurrent use of opioid pain medications with other opioid pain medications, benzodiazepines, or heroin or other nonpharmaceutical opioids can increase patients’ risk for overdose. Toxicology tests can provide information about drug use that is not reported by the patient. In addition, toxicology tests can assist clinicians in identifying when patients are not taking opioids prescribed for them, which might in certain cases indicate diversion or other clinically important issues such as difficulties with adverse effects. The most commonly drug-tested bodily specimen is urine. Oral fluid (saliva) testing also is available (*304*), although testing protocols using oral fluid are not as well established. On October 25, 2019, SAMHSA published guidelines for the inclusion of oral fluid specimens in toxicology testing programs of federal executive branch agencies (*305*), effective January 1, 2020. Toxicology testing results can be associated with outcomes and practices that harm patients (e.g., stigmatization and inappropriate termination from care). False positive and false negative presumptive results are not uncommon, a problem that can be compounded because clinicians commonly misinterpret results (*306*,*307*), leading to inappropriate consequences for patients. Urine toxicology tests do not provide accurate information about how much or what doses of opioids or other drugs a patient took. Testing for fentanyl is not available in widely used toxicology assays, potentially leading to false assurance. Ideally, clinicians would only test for substances for which results could affect patient management. However, it can be challenging for clinicians in many settings to tailor widely used toxicology panels to include the specific substances most relevant to clinical decisions for their patient. Toxicology testing costs are not always covered fully by insurance and can be a burden for patients, and clinician time is needed to interpret, confirm, and communicate results.

Experts from OWG had concerns that biases and disparities affecting which patients undergo toxicology testing could have disproportionately negative consequences among Black and Hispanic patients. In addition, testing costs would have the greatest consequences for patients with the least ability to pay. Because of these concerns, some experts said that grading the recommendation as category A could potentially reduce bias and disparities. However, others indicated that although universal application could mitigate bias in who is tested, it would not mitigate stigma associated with testing. In addition, experts had concerns about accuracy, clinician interpretation, testing costs, and potential for a delay in care while waiting for test results.

Because of these concerns, the recommendation is rated category B. However, clinicians, practices, and health systems should aim to minimize bias in its application and should not apply this recommendation differentially on the basis of assumptions about what they will learn about specific patients. Predicting risk is challenging, and available tools do not allow clinicians to reliably identify patients who are at low risk for substance use disorder (*7*). Rather, clinicians should consider toxicology test results as potentially useful data, in the context of other clinical information, for all patients and consider toxicology testing whenever its potential problems can be mitigated. For example, clinicians can become familiar with the drugs included in toxicology testing panels used in their practice and understand how to interpret results; practices and health systems can ensure a laboratorian or toxicologist is available to discuss unexpected results, that costs to patients are not burdensome, and that practice policies regarding testing and frequency can minimize bias. For example, routine use of testing with standardized policies at the practice or clinic level might help destigmatize their use. Because truly random testing might not be feasible in clinical practice, some clinics obtain a specimen at every visit but only send it for testing on a random schedule.

Before starting opioids and periodically (at least annually) during opioid therapy, clinicians should consider benefits and risks of toxicology testing to assess for prescribed opioids and other prescription and nonprescribed substances that increase risk for overdose when combined with opioids, including nonprescribed and illicit opioids and benzodiazepines. Before ordering toxicology testing, clinicians should have a plan for responding to unexpected results. Clinicians should explain to patients that toxicology testing will not be used punitively (e.g., will not be used to dismiss patients from care) and is intended to improve their safety. Clinicians should also explain expected results (e.g., presence of prescribed medication and absence of substances, including nonprescribed substances, not reported by the patient). Clinicians should ask patients about use of prescribed medications and other substances and ask whether there might be unexpected results. This will provide an opportunity for patients to provide information about changes in their use of prescribed opioids or other drugs.

In most situations, initial toxicology testing can be performed with a relatively inexpensive immunoassay panel that tests for opiates and benzodiazepines as classes and for multiple nonprescribed substances. Patients prescribed oxycodone or nonmorphine-based opioids (e.g., buprenorphine or methadone) require specific testing for those agents. The use of confirmatory testing can add costs and should be used when toxicology results will inform decisions with major clinical or nonclinical implications for the patient, a need exists to detect a specific opioid that is prescribed or that cannot be identified on standard immunoassays, or to confirm unexpected toxicology screening results for which there is no other explanation. Clinicians and health systems can work to minimize inequitable cost burdens for patients and limit specific testing to situations when it is necessary. Clinicians should be familiar with the compounds included in toxicology testing panels used in their practice and should understand how to interpret results. For example, a positive opiate immunoassay test result detects morphine, which might reflect patient use of morphine, codeine, or heroin, but this immunoassay does not detect synthetic opioids (e.g., fentanyl or methadone) and might not detect semisynthetic opioids (e.g., oxycodone or buprenorphine). Many laboratories use an oxycodone immunoassay that detects oxycodone and oxymorphone; however, these agents might need to be ordered or identified separately in a toxicology testing panel. In some cases, positive results for specific opioids might reflect metabolites from opioids the patient is taking and might not mean the patient is taking the specific opioid for which the test was positive. For example, hydromorphone is a metabolite of hydrocodone, and oxymorphone is a metabolite of oxycodone. Detailed considerations for interpretation of urine toxicology test results, including which tests to order and expected results, drug detection time in urine, and drug metabolism, have been published previously (*308*). A review including interpretation of oral fluid sample toxicology test results is also available (*304*). Restricting confirmatory testing to situations and substances for which results can reasonably be expected to affect patient management can reduce costs of toxicology testing.

Clinicians might want to discuss unexpected results with the local laboratory or toxicologist and should discuss unexpected results with the patient. Discussion with patients before specific confirmatory testing can sometimes yield a candid explanation of why a particular substance is present or absent and obviate the need for confirmatory testing on that visit. For example, a patient might explain that the test is negative for prescribed opioids because they felt opioids were no longer helping and discontinued them. If unexpected results are not explained, a confirmatory test using a method selective enough to differentiate specific opioids and metabolites (e.g., gas or liquid chromatography–mass spectrometry) might be warranted to clarify the situation.

Clinicians should use unexpected results to improve patient safety (e.g., change pain management strategy [see Recommendation 2], carefully weigh benefits and risks of reducing or continuing opioid dosage [see Recommendation 5], reevaluate more frequently [see Recommendation 7], offer naloxone [see Recommendation 8], and offer or refer patients for substance use disorder treatment [see Recommendation 12], all as appropriate). If tests for prescribed opioids are repeatedly negative, including confirmatory tests, and the clinician has verified that the patient is not taking the prescribed opioid, clinicians can discontinue the prescription without a taper and discuss options for safe disposal of unused opioids (*154*).

Clinicians should not dismiss patients from care on the basis of a toxicology test result. Dismissal could have adverse consequences for patient safety, potentially including the patient obtaining opioids from alternative sources and the clinician missing opportunities to facilitate treatment for a substance use disorder.

#### Recommendation 11

**Clinicians should use particular caution when prescribing opioid pain medication and benzodiazepines concurrently and consider whether benefits outweigh risks of concurrent prescribing of opioids and other central nervous system depressants (recommendation category: B; evidence type: 3).**

##### Implementation Considerations

- Although in some circumstances it might be appropriate to prescribe opioids to a patient who is also prescribed benzodiazepines (e.g., severe acute pain in a patient taking long-term, stable low-dose benzodiazepine therapy), clinicians should use particular caution when prescribing opioid pain medication and benzodiazepines concurrently. In addition, clinicians should consider whether benefits outweigh risks for concurrent use of opioids with other central nervous system depressants (e.g., muscle relaxants, nonbenzodiazepine sedative hypnotics, and potentially sedating anticonvulsant medications such as gabapentin and pregabalin).
- Buprenorphine or methadone for opioid use disorder should not be withheld from patients taking benzodiazepines or other medications that depress the central nervous system.
- Clinicians should check the PDMP for concurrent controlled medications prescribed by other clinicians (see Recommendation 9) and should consider involving pharmacists as part of the management team when opioids are coprescribed with other central nervous system depressants.
- In patients receiving opioids and benzodiazepines long term, clinicians should carefully weigh the benefits and risks of continuing therapy with opioids and benzodiazepines and discuss with patients and other members of the patient’s care team.
- Risks of concurrent opioid and benzodiazepine use are likely to be greater with unpredictable use of either medication, with use of higher-dosage opioids and higher-dosage benzodiazepines in combination, or with use with other substances including alcohol (compared with long-term, stable use of lower-dosage opioids and lower-dosage benzodiazepines without other substances).
- In specific situations, benzodiazepines can be beneficial, and stopping benzodiazepines can be destabilizing.
- Clinicians should taper benzodiazepines gradually before discontinuation because abrupt withdrawal can be associated with rebound anxiety, hallucinations, seizures, delirium tremens, and, rarely, death. The rate of tapering should be individualized.
- If benzodiazepines prescribed for anxiety are tapered or discontinued, or if patients receiving opioids require treatment for anxiety, evidence-based psychotherapies (e.g., cognitive behavioral therapy), specific antidepressants or other nonbenzodiazepine medications approved for anxiety, or both, should be offered.
- Clinicians should communicate with other clinicians managing the patient to discuss the patient’s needs, prioritize patient goals, weigh risks of concurrent benzodiazepine and opioid exposure, and coordinate care.

##### Supporting Rationale

Benzodiazepines and opioids both cause central nervous system depression, and benzodiazepines can potentiate opioid-induced decreases in respiratory drive. Epidemiologic studies find concurrent benzodiazepine use in large proportions of opioid-related overdose deaths (*203*,*309*,*310*). The clinical evidence reviews identified three cohort studies that found an association between concurrent use of benzodiazepines and opioids versus opioids alone and increased risk for overdose (*7*). A case-cohort study found concurrent benzodiazepine prescription with opioid prescription to be associated with a near-quadrupling of risk for overdose death compared with opioid prescription alone (*311*). The clinical evidence reviews did not find studies evaluating the effectiveness of avoiding coprescribing of benzodiazepines and opioids on risk for overdose (*7*). The clinical evidence reviews identified three observational studies that found an association between concurrent use of gabapentinoids and opioids versus opioids alone and increased risk for overdose, with higher risks at increased gabapentinoid doses (*7*).

Experts from OWG noted that rather than necessarily being a direct cause of overdose, benzodiazepines might serve as a marker of risk for overdose because of underlying conditions, in specific situations benzodiazepines can be beneficial, and that stopping benzodiazepines can be destabilizing. In addition, experts noted that long-term, stable use might be safer than erratic, unpredictable use. Because of these considerations, multiple experts indicated that recommending extreme caution with concurrent prescription of opioid pain medications and benzodiazepines was more appropriate than a recommendation to avoid prescribing opioid pain medication and benzodiazepines concurrently and that category B would be more appropriate than category A for this recommendation.

Although in certain circumstances it might be appropriate to prescribe opioids to a patient receiving benzodiazepines (e.g., severe acute pain in a patient taking long-term, stable low-dosage benzodiazepine therapy), clinicians should use particular caution when prescribing opioid pain medication and benzodiazepines concurrently. In addition, because other central nervous system depressants (e.g., muscle relaxants, nonbenzodiazepine sedative hypnotics, and potentially sedating anticonvulsant medications such as gabapentin and pregabalin) (*312*) can potentiate respiratory depression associated with opioids, clinicians should consider whether benefits outweigh risks of concurrent use of these medications. Clinicians should check PDMPs for concurrent controlled medications prescribed by other clinicians (see Recommendation 9) and should consider involving pharmacists as part of the management team when opioids are coprescribed with other central nervous system depressants.

In patients receiving opioids and benzodiazepines long-term, clinicians should carefully weigh the benefits and risks of continuing therapy with opioids and benzodiazepines and discuss with patients and other members of the patient’s care team, as appropriate. In specific situations, benzodiazepines can be beneficial, and stopping benzodiazepines can be destabilizing. As emphasized in an FDA advisory (*313*), buprenorphine or methadone for opioid use disorder should not be withheld from patients taking benzodiazepines or other medications that depress the central nervous system. Whereas the combined use of these medications increases risks, the harm caused by untreated opioid use disorder can outweigh these risks.

If risks are determined to outweigh benefits of continuing opioids for pain and benzodiazepine therapy at current dosages, decisions about tapering medications (e.g., whether to taper opioids first, taper benzodiazepines first, or consider carefully transitioning from full agonist opioids to buprenorphine before tapering benzodiazepines) should be individualized and reevaluated over time. Considerations include patient priorities, the patient’s clinical considerations, the patient’s response to therapeutic changes, consultation with other clinicians managing the patient’s care, and, consultation with other specialists (e.g., an addiction specialist) if needed. Clinicians should taper benzodiazepines gradually before discontinuation because abrupt withdrawal can be associated with rebound anxiety, hallucinations, seizures, delirium tremens, and, rarely, death (*222*,*223*). Tapering rates should be individualized. Examples of benzodiazepine tapers and tips for managing benzodiazepine withdrawal are available (*314*). Cognitive behavioral therapy increases tapering success rates and might be particularly helpful for patients struggling with a benzodiazepine taper (*315*). If benzodiazepines prescribed for anxiety are tapered or discontinued, or if patients receiving opioids require treatment for anxiety, evidence-based psychotherapies (e.g., cognitive behavioral therapy), specific antidepressants or other nonbenzodiazepine medications approved for anxiety, or both, should be offered. Clinicians should communicate with mental health professionals managing the patient to discuss the patient’s needs, prioritize patient goals, weigh risks of concurrent benzodiazepine and opioid exposure, and coordinate care.

#### Recommendation 12

**Clinicians should offer or arrange treatment with evidence-based medications to treat patients with opioid use disorder. Detoxification on its own, without medications for opioid use disorder, is not recommended for opioid use disorder because of increased risks for resuming drug use, overdose, and overdose death (recommendation category: A; evidence type: 1).**

##### Implementation Considerations

- Although stigma can reduce the willingness of persons with opioid use disorder to seek treatment, opioid use disorder is a chronic, treatable disease from which persons can recover and continue to lead healthy lives.
- If clinicians suspect opioid use disorder, they should discuss their concern with their patient in a nonjudgmental manner and provide an opportunity for the patient to disclose related concerns or problems.
- Clinicians should assess for the presence of opioid use disorder using DSM-5 criteria.
- For patients meeting criteria for opioid use disorder, particularly if moderate or severe, clinicians should offer or arrange for patients to receive evidence-based treatment with medications for opioid use disorder.
- Clinicians should not dismiss patients from their practice because of opioid use disorder because this can adversely affect patient safety.
- Medication treatment of opioid use disorder has been associated with reduced risk for overdose and overall deaths. Identification of opioid use disorder represents an opportunity for a clinician to initiate potentially life-saving interventions, and the clinician should collaborate with the patient regarding their safety to increase the likelihood of successful treatment.
- For pregnant persons with opioid use disorder, medication for opioid use disorder (buprenorphine or methadone) is the recommended therapy and should be offered as early as possible in pregnancy to prevent harms to both the patient and the fetus.
- Clinicians unable to provide treatment themselves should arrange for patients with opioid use disorder to receive care from a substance use disorder treatment specialist (e.g., an office-based buprenorphine or naltrexone treatment provider), or from an opioid treatment program certified by SAMHSA to provide methadone or buprenorphine for patients with opioid use disorder.
- All clinicians, and particularly clinicians prescribing opioids in communities without sufficient treatment capacity for opioid use disorder, should obtain a waiver to prescribe buprenorphine for opioid use disorder.
- Clinicians prescribing opioids should identify treatment resources for opioid use disorder in the community, establish a network of referral options that span the levels of care that patients might need to enable rapid collaboration and referral, when needed, and work together to ensure sufficient treatment capacity for opioid use disorder at the practice level.
- Although identification of an opioid use disorder can alter the expected benefits and risks of opioid therapy for pain, patients with co-occurring pain and opioid use disorder require ongoing pain management that maximizes benefits relative to risks.

##### Supporting Rationale

Opioid use disorder (previously known as opioid abuse or opioid dependence in the *Diagnostic and Statistical Manual of Mental Disorders*, *Fourth Edition* [DSM-IV]) (*316*) is defined in DSM-5 as a problematic pattern of opioid use leading to clinically significant impairment or distress (*317*). Treatment with opioids for pain is associated with increased risk for opioid use disorder, particularly if opioids are prescribed for >90 days (*54*). A systematic review found the rate of opioid addiction among patients with chronic pain averaged 8%–12% in studies published during 2000–2013 (*318*). More recent studies have found prevalence estimates of 23.9%–26.5% for any prescription opioid use disorder and 5.2%–9.0% for moderate to severe opioid use disorder (using DSM-5 diagnostic criteria) among adults receiving long-term opioid therapy for pain, with slightly lower prevalence (21.5% for any and 4.2% for moderate to severe opioid use disorder) in clinics with more consistent use of risk reduction practices (*319*,*320*).

Opioid use disorder is manifested by at least two of 11 defined criteria occurring within a year (*317*):

1. Opioids are often taken in larger amounts or over a longer period than was intended.
2. There is a persistent desire or unsuccessful attempts to cut down or control opioid use.
3. A great deal of time is spent in activities necessary to obtain the opioid, use the opioid, or recover from its effects.
4. Craving, or a strong desire or urge to use opioids.
5. Recurrent opioid use resulting in a failure to fulfill major role obligations at work, school, or home.
6. Continued opioid use despite having persistent or recurrent social or interpersonal problems caused or exacerbated by the effects of opioids.
7. Important social, occupational, or recreational activities are given up or reduced because of opioid use.
8. Recurrent opioid use in situations in which it is physically hazardous.
9. Continued opioid use despite knowledge of having a persistent or recurrent physical or psychological problem that is likely to have been caused or exacerbated by the substance.
10. Tolerance, as defined by either of the following:
  - a need for markedly increased amounts of opioids to achieve intoxication or desired effect, or
  - a markedly diminished effect with continued use of the same amount of an opioid.
11. Withdrawal, as manifested by either of the following:
  - the characteristic opioid withdrawal syndrome, or
  - opioids (or a closely related substance) are taken to relieve or avoid withdrawal symptoms.

Criteria 10 and 11 are not considered to be met for those persons taking opioids solely under appropriate medical supervision (*317*). Severity is specified as mild (2–3 criteria), moderate (4–5 criteria), or severe (≥6 criteria) (*317*).

FDA-approved medications indicated for the treatment of opioid use disorder include buprenorphine (a partial agonist opioid), methadone (a full agonist opioid), and naltrexone (an opioid antagonist). Experts from OWG stated that partial agonist opioid, full agonist opioid, and opioid antagonist treatment should not be framed as equal options for opioid use disorder, noting that partial and full agonist opioid treatments have stronger evidence for better outcomes, do not require abstinence, have less challenges with initiation, and are much more widely used than opioid antagonist treatment. Clinical evidence reviews found evidence on the effectiveness of interventions (e.g., medications and behavioral treatments) for opioid use disorder related to prescription opioids to be limited (*7*). However, moderate-quality evidence indicated buprenorphine (a partial agonist opioid) and methadone (a full agonist opioid) to be effective in preventing return to drug use among patients with opioid use disorder involving heroin (*321*–*323*), although the presence of pain among patients in these studies is generally not described. In addition, a small number of studies have evaluated buprenorphine for patients with prescription opioid dependence (using DSM-IV criteria) (*316*) and found it to be effective in preventing return to drug use (*324*,*325*). One study found that among persons with opioid use disorder, previous prescription opioid use predicts stabilization on buprenorphine (*326*). Another trial that performed buprenorphine initiation and then randomized patients to buprenorphine taper versus maintenance was terminated early without reporting of planned outcomes because all patients randomized to the taper arm switched to maintenance or experienced a return to drug use; five of six patients in the maintenance arm completed the trial (*327*). In another trial identified by the clinical evidence reviews, no difference was found between buprenorphine/naloxone and methadone in likelihood of retention in the study and in pain, function, or self-reported side effects (*328*). Buprenorphine and methadone treatment of opioid use disorder has been associated with reduced overdose deaths (*329*) and reduced all-cause deaths (*330*). Naltrexone (an opioid antagonist) also can be used for opioid use disorder, particularly for highly motivated persons (*331*,*332*). Naltrexone blocks the effects of opioids if they are used. Naltrexone has not been evaluated in persons with concomitant pain and opioid use disorder, and opioid medications for pain generally cannot be used in patients receiving naltrexone. Naltrexone requires adherence to monthly, long-acting injections. The effectiveness of oral naltrexone can be limited by poor medication adherence (*332*), and oral naltrexone should not be used except under very limited circumstances (*96*) (e.g., for patients who would be able to comply with observed daily dosing to enhance adherence) (*96*,*317*). Naltrexone also must be started after full withdrawal from opioids, which is a challenge for some patients; however, for patients who have completed or are able to complete withdrawal, naltrexone has comparable effectiveness as buprenorphine in prevention of return to drug use (*333*).

Certain studies suggest that using behavioral therapies in combination with medications for opioid use disorder can reduce opioid misuse and increase retention during treatment (*334*,*335*). At the same time, a study of treatment for prescription opioid dependence (using DSM-IV criteria) (*316*) found buprenorphine treatment combined with standard medical management (including basic counseling recommending abstinence and self-help group participation) as effective as buprenorphine combined with more intensive opioid dependence counseling (i.e., addiction, recovery, and prevention of return to drug use education with self-help and lifestyle change recommendations, interactive exercises, and take-home assignments delivered by trained substance use treatment or mental health professionals in 45–60 minute sessions using drug counseling manuals with demonstrated efficacy); neither standard medical management nor opioid dependence counseling alone, without buprenorphine, was effective in preventing return to drug use (*325*). Recommendations for treatment of opioid use disorder include assessing the patient’s psychosocial needs and offering or referring the patient to psychosocial treatment in collaboration with qualified behavioral health care providers based on those needs; however, a patient’s decision to decline psychosocial treatment or the absence of available psychosocial treatment should not preclude or delay medications for opioid use disorder (*96*). Additional recommendations have been published on goals, components of, and types of effective psychosocial treatment to use in conjunction with pharmacologic treatment of opioid use disorder (*96*).

If clinicians suspect opioid use disorder on the basis of patient concerns or behaviors or on findings in PDMP data (see Recommendation 9) or from toxicology testing (see Recommendation 10), they should discuss their concern with their patient and provide an opportunity for the patient to disclose related concerns or problems. Clinicians should assess for the presence of opioid use disorder using DSM-5 criteria (*317*). Opioid use disorder can coexist with other substance use disorders, and patients who are actively using substances during opioid use disorder treatment might require greater support, potentially including involvement of an addiction specialist (*96*). Clinicians should ask about use of alcohol and other substances (see Recommendation 8). Alternatively, clinicians can arrange for a substance use disorder treatment specialist to assess for the presence of opioid and other substance use disorders.

For patients meeting criteria for opioid use disorder, particularly if moderate or severe, clinicians should offer or arrange for patients to receive evidence-based treatment with medications for opioid use disorder. Patients with opioid use disorder might benefit from counseling and referrals to mutual help groups such as Narcotics Anonymous (*336*), although this should not take the place of treatment with medication. Clinicians also should offer naloxone and training on proper use for overdose reversal to patients with opioid use disorder and to their household members and significant others (*96*) (see Recommendation 8). Clinicians should not dismiss patients from their practice because of opioid use disorder because this can adversely affect patient safety. Identification of opioid use disorder represents an opportunity for a clinician to initiate potentially life-saving interventions, and it is important for the clinician to collaborate with the patient regarding their safety to increase the likelihood of successful treatment. Detoxification on its own, without medications for opioid use disorder, is not recommended for opioid use disorder because of increased risks for return to drug use, overdose, and overdose death (*96*).

For pregnant persons with opioid use disorder, medications for opioid use disorder (buprenorphine or methadone) have been associated with improved maternal outcomes and should be offered as early as possible in pregnancy to prevent harms to both the patient and the fetus (see Recommendation 8) (*133*,*220*). Previous recommendations have suggested that transmucosal buprenorphine (without naloxone) is preferred during pregnancy to avoid potential prenatal exposure to naloxone, especially if injected, and evidence on the safety of naloxone in pregnant persons remains limited (*96*,*274*). However, combination buprenorphine/naloxone products are frequently used, a systematic review did not find reports of serious maternal or neonatal outcomes associated with maternal buprenorphine/naloxone use (*337*), and experts have noted that combination products are likely to be safe and effective for pregnant persons when taken as prescribed (*96*,*274*). ACOG also recommends that if a person is stable on naltrexone before pregnancy, the decision regarding whether to continue naltrexone treatment during pregnancy should involve a careful discussion between the clinician and the patient, weighing the limited safety data on naltrexone with the potential risk for return to drug use with discontinuation of treatment (*274*). For persons receiving buprenorphine or methadone for opioid use disorder and considering breastfeeding, AAP recommends breastfeeding be supported if there has been no return to drug use for ≥90 days and there are no other contraindications, considered if there has been no return to drug use within 30–90 days, and discouraged if there is active substance use or has been a return to drug use within the last 30 days (*280*).

In April 2021, to expand access to buprenorphine, the *Practice Guidelines for the Administration of Buprenorphine for Treating Opioid Use Disorder* (*338*) exempted eligible physicians, physician assistants, nurse practitioners, clinical nurse specialists, certified registered nurse anesthetists, and certified nurse midwives from previous Controlled Substances Act certification requirements related to training, counseling and other ancillary services (i.e., psychosocial services). To prescribe buprenorphine for opioid use disorder for up to 30 patients in an office-based setting, clinicians can forgo or choose to undertake training but must still receive a waiver from SAMHSA. Information about qualifications and the process to obtain a waiver are available from SAMHSA (*339*).

Additional recommendations have been published on initiation, use, and monitoring of buprenorphine treatment for opioid use disorder (*96*,*336*). Buprenorphine for treatment of opioid use disorder is usually combined with naloxone in a sublingual or buccal film or tablet (e.g., Suboxone), to reduce the potential for misuse of buprenorphine when injected. Naloxone is poorly absorbed orally; however, if buprenorphine/naloxone is manipulated and injected, naloxone can trigger opioid withdrawal (*340*). In 2018, long-acting injectable formulations of buprenorphine became available (*341*). As a partial agonist, buprenorphine should generally not be initiated until there are objective signs of withdrawal, to avoid precipitating withdrawal. As an alternative for patients not yet in opioid withdrawal, certain studies have described a low-dose initiation approach (sometimes referred to as microdosing) (*342*,*343*) to avoid precipitating withdrawal when initiating buprenorphine, although evidence regarding this approach is limited. Low-dose buprenorphine initiation is a potential option for patients with opioid use disorder who are taking opioid medications for pain. With this dosing strategy, full agonist opioids can be continued while buprenorphine is initiated, and the patient does not need to experience opioid withdrawal symptoms. For standard (not low-dose) buprenorphine initiation, after objective signs of withdrawal are observed, buprenorphine should be initiated (*96*) and titrated upward under supervision at approximately 2-hour intervals as needed to control withdrawal symptoms. Protocols for initiating buprenorphine by patients at home after an initial encounter with a clinician to establish the diagnosis of opioid use disorder and discuss medication options are in use by more experienced clinicians (*344*).

Importantly, opioid dosage thresholds for caution in the treatment of pain are not applicable to opioid agonist treatment of opioid use disorder (*345*) because recommended dosages of methadone and buprenorphine for opioid use disorder (*96*) differ from those for pain management. No recommended duration limit exists for treatment of opioid use disorder with buprenorphine or methadone, and discontinuation is associated with risks for return to drug use and opioid overdose (*96*). If discontinued, buprenorphine should be tapered very gradually (over several months) (*96*).

Compared with buprenorphine, which can be prescribed by clinicians with a waiver in any setting or dispensed from a SAMHSA-certified opioid treatment program, ongoing methadone treatment for opioid use disorder can only be provided through an opioid treatment program. As short-term exceptions, any clinician may administer (but not prescribe) methadone or buprenorphine to treat acute opioid withdrawal for up to 3 days, while working to refer the patient to opioid use disorder treatment (*346*). Previously, up to a 1-day supply could be administered per day for up to 3 days; in December 2020, Congress directed the Drug Enforcement Administration (DEA) to revise regulations to allow for a 3-day supply of medication to be dispensed at one time (*347*); DEA subsequently advised practitioners how to request exceptions to the 1-day supply limitation pending amendment of 21 CFR 1306.07(b) (*348*). Patients already receiving treatment for opioid use disorder and admitted for other medical reasons may continue to directly receive methadone or buprenorphine treatment in an emergency department or in a hospital throughout inpatient hospitalization (*336*,*346*,*349*).

Naltrexone does not require a waiver and can be prescribed in any setting. Additional recommendations have been published previously on naltrexone treatment for opioid use disorder (*96*). A minimum of 7–10 days free of opioids is recommended before the first naltrexone dose to avoid precipitation of severe opioid withdrawal (*350*). Extended-release injectable naltrexone is typically administered every 4 weeks by deep intramuscular injection in the gluteal muscle at 380 mg per injection (*96*), alternating buttocks for each subsequent injection (*350*). Certain patients, including those who metabolize naltrexone more rapidly, might benefit from dosing as frequently as every 3 weeks (*96*). Oral naltrexone is no longer recommended and should not be used except under very limited circumstances (*96*). No recommended duration limit exists for treatment of opioid use disorder with naltrexone. If discontinued, naltrexone can be stopped abruptly without precipitating withdrawal symptoms (*96*). Clinicians should warn patients who discontinue naltrexone of the risk for potentially fatal opioid overdose if opioid use is resumed (*96*), because of the loss of tolerance to the previous opioid dosage.

Clinicians are strongly encouraged to provide medication treatment for their patients with opioid use disorder. Those unable to provide treatment themselves should arrange for patients with opioid use disorder to receive care from a colleague who is able to provide treatment, from a substance use disorder treatment specialist (e.g., an office-based buprenorphine or naltrexone treatment clinician), or from an opioid treatment program certified by SAMHSA to provide methadone or buprenorphine for patients with opioid use disorder. Resources to help clinicians arrange for treatment include SAMHSA’s buprenorphine physician locator (https://www.samhsa.gov/medication-assisted-treatment/find-treatment/treatment-practitioner-locator) and SAMHSA’s Opioid Treatment Program Directory (https://dpt2.samhsa.gov/treatment/directory.aspx). Clinicians should assist patients in finding qualified treatment specialists, should arrange for patients to follow up with these specialists, and should coordinate continuing care with these specialists. Rapidly identifying appropriate care can be challenging. Treatment need in a community is often not met by capacity to provide buprenorphine or methadone therapy (*351*). Clinicians prescribing opioids in communities without sufficient treatment capacity for opioid use disorder should obtain a waiver to prescribe buprenorphine. SAMHSA’s Providers Clinical Support System (https://pcssnow.org/) offers training, technical assistance, and mentors to assist clinicians in assessment for and treatment of substance use disorders, specifically opioid use disorder, and on the interface of pain and opioid misuse. Clinicians prescribing opioids should identify treatment resources for substance use disorders including opioid use disorders in the community, establish a network of referral options that span the levels of care that patients might need to enable rapid collaboration and referral, when needed, and work together to ensure sufficient treatment capacity at the practice level.

#### Management of Opioid Misuse That Does Not Meet Criteria for Opioid Use Disorder

Clinicians can have challenges distinguishing between opioid misuse behaviors without opioid use disorder and mild or moderate opioid use disorder (*352*). For patients with opioid misuse that does not meet criteria for opioid use disorder (e.g., taking opioids in larger amounts than intended without meeting other criteria for opioid use disorder), clinicians should reassess the patient’s pain, ensure that therapies for pain management have been optimized (see Recommendation 2), discuss with patients, and carefully weigh benefits and risks of continuing opioids at the current dosage (see Recommendation 5). For patients who choose to but are unable to taper, clinicians can reassess for opioid use disorder and offer buprenorphine treatment or refer for buprenorphine or methadone treatment if criteria for opioid use disorder are met. Even without a diagnosis of opioid use disorder, transitioning to buprenorphine for pain also can be considered because of reduced risk for overdose with buprenorphine compared with risk associated with full agonist opioids (see Recommendation 5).

#### Pain Management for Patients with Opioid Use Disorder

Although identification of an opioid use disorder can alter the expected benefits and risks of opioid therapy for pain, patients with co-occurring pain and substance use disorder require ongoing pain management that maximizes benefits relative to risks. Clinicians should use nonpharmacologic and nonopioid pharmacologic pain treatments as appropriate (*96*) (see Recommendations 1 and 2) to provide optimal pain management. For patients with pain who have an active opioid use disorder but are not in treatment, clinicians should consider buprenorphine or methadone treatment for opioid use disorder, which also can help with concurrent management of pain (*96*). For patients who are treated with buprenorphine for opioid use disorder and experience acute pain, clinicians can consider temporarily increasing the buprenorphine dosing frequency (e.g., to twice per day) (*96*) to help manage pain because the duration of effects of buprenorphine is shorter for pain than for suppression of withdrawal (*242*). For severe acute pain (e.g., from trauma or unplanned major surgery) in patients receiving buprenorphine for opioid use disorder, clinicians can consider additional as-needed doses of buprenorphine. In supervised settings, adding a short-acting full agonist opioid to the patient’s regular dosage of buprenorphine can be considered without discontinuing the patient’s regular buprenorphine dosage; however, if a decision is made to discontinue buprenorphine to allow for more *µ*-opioid receptor availability, patients should be monitored closely because high doses of a full agonist opioid might be required, potentially leading to oversedation and respiratory depression as buprenorphine’s partial agonist effect lessens (*96*). For patients receiving naltrexone for opioid use disorder, short-term use of higher-potency nonopioid analgesics (e.g., NSAIDs) can be considered to manage severe acute pain (*96*). Patients receiving methadone for opioid use disorder who require additional opioids as treatment for severe acute pain management should be monitored carefully, and when feasible, should optimally be treated by a clinician experienced in the treatment of pain in consultation with their opioid treatment program (*96*). The ASAM National Practice Guideline for the Treatment of Opioid Use Disorder (2020 Focused Update) provides additional recommendations (see Part 9) (*96*) for the management of patients receiving medications for opioid use disorder who have planned surgeries for which nonopioid therapies are not anticipated to provide sufficient pain relief.

## Conclusion and Future Directions

CDC indicated the intent to evaluate and reassess the 2016 CDC Opioid Prescribing Guideline as new evidence became available and determine when sufficient new evidence would prompt an update (*56*). CDC funded AHRQ to conduct systematic reviews of the scientific evidence. The following five areas were assessed: 1) noninvasive nonpharmacologic treatments for chronic pain, 2) nonopioid pharmacologic treatments for chronic pain, 3) opioid treatments for chronic pain, 4) treatments for acute pain, and 5) acute treatments for episodic migraine (*7*–*11*). An update to the 2016 CDC Opioid Prescribing Guideline was warranted on the basis of these reviews.

The new evidence reviews conducted by AHRQ’s Evidence-based Practice Centers affirmed the appropriateness of the recommendations in the 2016 CDC Opioid Prescribing Guideline for using opioids to treat chronic pain. The reviews also prompted CDC to modify the recommendations to include acute and subacute pain more explicitly. This updated clinical practice guideline also includes a new topline recommendation for patients who are already receiving ongoing opioid therapy for pain. Specifically, the clinical practice guideline outlines how clinicians and patients should work together in assessing the benefits and risks of continued opioid use and if or when to taper opioids to a lower dosage or discontinue opioids altogether in accordance with the HHS Tapering Guide (*219*,*353*).

Four key areas are covered in this clinical practice guideline for prescribing of opioid pain medication for patients aged ≥18 years for pain, excluding pain management related to sickle cell disease, cancer-related pain treatment, palliative care, and end-of-life care. These areas are 1) determining whether or not to initiate opioids for pain; 2) selecting opioids and determining opioid dosages; 3) deciding duration of initial opioid prescription and conducting follow-up; and 4) assessing risk and addressing potential harms of opioid use. In addition, five guiding principles were identified to inform implementation across recommendations. These guiding principles focus on 1) the appropriate treatment of pain; 2) flexibility to meet the care needs and clinical circumstances of each patient; 3) a multimodal and multidisciplinary approach to pain management; 4) avoiding misapplication of the clinical practice guideline beyond its intended use; and 5) vigilance in attending to health inequities and ensuring access to appropriate, affordable, diversified, coordinated, and effective nonpharmacologic and pharmacologic pain treatment for all persons.

A central tenet of this clinical practice guideline is that acute, subacute, and chronic pain needs to be appropriately and effectively treated regardless of whether opioids are part of a treatment regimen. Clinicians should select nonpharmacologic or pharmacologic treatment modalities, or both, that maximize patient safety and optimize outcomes in pain, function, and quality of life. A multimodal and multidisciplinary approach to pain management that considers the biologic, psychological, and social characteristics of each person is critical (*6*). The care provided needs to be individualized and person centered (*6*). Clinicians and patients should work together to identify treatment goals, including functional goals, and tailor an approach that considers both the benefits and risks of available options (*6*). Progress should be monitored over time and treatment protocols adjusted accordingly. Health systems and payers can work to ensure multimodal treatment options are available, accessible, and reimbursed for patients. Public and private payers can support a broader array of nonpharmacologic interventions such as exercise, multidisciplinary rehabilitation, mind-body interventions, cognitive behavioral therapy, and certain complementary and integrative medicine therapies (e.g., acupuncture and spinal manipulation) that increasingly are known to be effective (*9*). Reimbursement often is cited as a principle barrier to why these nonpharmacologic treatments are not more widely used (*9*).

An integral part of providing access to and delivery of high-quality health care, including pain treatment, is understanding how the social determinants of health influence the health care provided and the differential outcomes observed (*354*). Social, economic, educational, and neighborhood-level factors might create and exacerbate health inequities that certain persons experience throughout their lives (*354*). These social determinants of health are borne out of historical and contemporary injustices that advantage some and disadvantage others in society, leading to the systemic marginalization or oppression of some groups (*355*). These inequities affect persons from some racial and ethnic groups, women, persons living in rural areas, persons experiencing homelessness, persons with disabilities, persons with substance use disorders, justice-involved populations, persons with diverse sexual orientation, identity, or gender, and non-U.S. born persons, among others (*356*).

Outcomes such as function and quality of life also are influenced by the health care context (*354*). Differential access to and coverage for high-quality, culturally and linguistically appropriate, health-literate care might influence attitudes toward health care and use of available services (*354*). Prejudice, bias, discrimination, and stereotyping by clinicians, practices, health systems, and payers serve to reinforce these health disparities (*355*). Clinicians, practices, health systems, and payers should attend to health inequities to protect patient safety; guard against unnecessary risks; and ensure access to appropriate, diversified, effective nonpharmacologic and pharmacologic pain management options that are person centered, affordable, accessible, and well coordinated. This begins with raising awareness and acknowledging the presence of these inequities, strengthening patient-clinician communication, leveraging community health workers, implementing multidisciplinary care teams, tracking and monitoring performance measures, and integrating quality improvement initiatives that support and invest in guideline-concordant care for all persons (*355*).

To avoid unintended consequences for patients, this clinical practice guideline should not be misapplied, or policies derived from it, beyond its intended use (*67*). Examples of misapplication or inappropriate policies include being inflexible on opioid dosage and duration, discontinuing or dismissing patients from a practice, rapidly and noncollaboratively tapering patients who might be stable on a higher dosage, and applying recommendations to populations that are not a focus of the clinical practice guideline (e.g., patients with cancer-related pain, patients with sickle cell disease, or patients during end-of-life care) (*67*).

This clinical practice guideline provides overarching voluntary recommendations on the use of opioids to manage pain. To assist in the uptake and understanding of this new clinical practice guideline, CDC will provide tools and resources for clinicians, health systems, patients, and others on the use of opioid and nonopioid pain treatments. The uptake and widespread use of the 2016 CDC Opioid Prescribing Guideline hinged on its successful dissemination, and CDC supported its translation and integration in clinical practice. CDC produced a checklist and mobile app so clinicians could more readily apply guideline recommendations; developed fact sheets, posters, and public service announcements to make the guideline more accessible and understandable to clinicians and patients; and developed a 14-module interactive, web-based training with self-paced learning, case-based content, knowledge checks, and integrated resources for clinicians (*57*). Updated and new resources and tools will align with this new clinical practice guideline and will support health equity.

CDC will work with public and private payers by sharing evidence that can be used to inform decisions about coverage for nonpharmacologic treatments, access to nonopioid pain medication, support for patient counseling and coordination of care, access to evidence-based treatments of opioid use disorder, and availability of multidisciplinary and multimodal care. Robust coverage and access (e.g., limited utilization management and cost sharing for evidence-based treatments) and decision support (e.g., adjustment of EHR prescribing defaults) can be used to facilitate and encourage evidence-based treatments as default treatments for pain (*357*,*358*).

This clinical practice guideline updates and expands the recommendations in the 2016 CDC Opioid Prescribing Guideline using the best available evidence as interpreted and informed by expert opinion and attending to the values and preferences expressed by patients, caregivers, and clinicians. Although the strength of the evidence is sometimes low quality and research gaps remain (Box 5), clinical scientific evidence continues to advance and supports the recommendations in this clinical practice guideline (*6*–*11*,*359*).

BOX 5 **Areas for additional research to build the evidence base for optimal pain management**

- Efficacy of screening tools to assess risk for opioid misuse and developing an opioid use disorder.
- Effective management of patients on high-dosage opioids, the application of multidisciplinary and multimodal models of pain treatment, and service delivery modalities including telehealth.
- Long-term comparative effectiveness of pharmacologic and nonpharmacologic therapies for chronic pain, including effects of treatment combinations, dosage variation, and comorbidities.
- Comparative effectiveness and comparative risks of partial agonist opioids (e.g., buprenorphine) versus full agonist opioids for pain.
- Comparative effectiveness and risks of interventional procedures as part of a comprehensive pain management plan.
- Effects of therapies on nonpain outcomes.
- Treatment outcomes for specific pain conditions and how benefits and risks of therapies vary among subpopulations.
- Adapting evidence-based opioid prescribing and pain management strategies to meet the needs of special populations, including persons from some racial and ethnic groups, older adults, and persons living in rural communities.
- Effectiveness of clinician and health system strategies to promote equitable access to high-quality pain management.
- Improved diagnostics in measuring pain.
- Enhanced clinician and patient education about pain and the use of opioids, and the assessment of practice-level strategies in health systems to improve management and care coordination for patients on opioid therapy.
- Transition from acute to chronic pain and how to apply effective diagnostic, preventive, and therapeutic approaches.
- Effects of stigma as a barrier for treating pain and receiving treatment for an opioid use disorder, and effective ways to counter the effects of stigma on access to treatment for pain and opioid use disorder.

The principal aim of this clinical practice guideline is to ensure persons have equitable access to safe and effective pain management that improves their function and quality of life while illuminating and reducing risks associated with prescription opioids. CDC will evaluate this clinical practice guideline to identify the effects of the recommendations on clinician and patient outcomes and on health disparities, including intended and unintended consequences. Communication between clinicians and patients about the benefits and risks of opioids should be central to treatment decisions for patients in pain. This clinical practice guideline can help inform those decisions and assist clinicians in meeting the unique needs of each person. CDC will revisit this clinical practice guideline when remaining evidence gaps have sufficiently been addressed and another update is warranted.

## Footnotes

* Financial conflict of interest means a significant financial interest that could directly and significantly affect the design, conduct, or reporting of Public Health Service-funded research (42 CFR 50.603). Although certain members reported receiving research support totaling or equivalent to more than $10,000, SBIU determined these activities did not constitute a substantial conflict of interest pertaining to the content of this clinical practice guideline.

## Appendix Primary Clinical Questions, Detailed Methods, and Findings for the Systematic and Contextual Evidence Reviews

### Primary Clinical Questions

Across reviews, the main outcomes were pain, function, and quality of life. Harms varied depending on the therapy evaluated but included serious adverse events when reported; for opioids, key harms included overdose and harms related to opioid use disorder. The reviews of therapies for chronic pain assessed outcomes at short- (1 to <6 months), intermediate- (6 to <12 months), and long-term follow-up (≥12 months). The reviews of therapies for acute pain assessed outcomes at <1 day, 1 day to <1 week, 1 week to <2 weeks, and 2–4 weeks; the review of treatments for acute nonmigraine pain also evaluated outcomes at ≥4 weeks. All reviews included key questions (KQs) or subquestions on how benefits and harms varied according to demographic (age, sex, race), clinical (severity and duration of pain, medical and psychiatric comorbidities, concomitant medications), and intervention (dose, duration, intensity) characteristics.

The systematic clinical evidence reviews addressed questions regarding the effectiveness and comparative effectiveness of noninvasive nonpharmacologic treatments; nonopioid pharmacologic treatments; and opioid treatments for chronic pain, acute pain, and episodic migraine pain (details including questions are available in the full AHRQ reports) (*1*–*5*).

### Opioids for Chronic Pain

- The effectiveness and comparative effectiveness (benefits [KQ 1] and harms [KQ 2]) of long-term opioid therapy versus placebo, no opioid therapy, or nonopioid therapy.
- The comparative effectiveness of various opioid dosing strategies (KQ3):
  - Different methods for initiating and titrating opioids
  - Short-acting versus long-acting and extended-release opioids
  - Different long-acting opioids
  - Short- acting plus long-acting versus long-acting opioid alone
  - Scheduled, continuous versus as-needed dosing
  - Opioid dose escalation versus dose maintenance or use of dose thresholds
  - Opioid rotation versus maintenance
  - Different strategies for treating acute exacerbations of chronic pain
  - Decreasing opioid doses or tapering off opioids versus continuation of opioids
  - Different tapering protocols and strategies
  - Different opioid dosages and durations of therapy
- The accuracy of instruments for predicting risk for opioid overdose, addiction, abuse, or misuse; the effectiveness of risk prediction instruments; the effectiveness of various risk mitigation strategies; and comparative effectiveness of strategies for managing patients with opioid use disorder (KQ 4). The following are risk mitigation strategies:
  - Opioid management plans
  - Patient education
  - Urine drug screening
  - Use of prescription drug monitoring program (PDMP) data
  - Use of monitoring instruments in patients prescribed opioids
  - More frequent monitoring intervals
  - Pill counts
  - Use of abuse-deterrent formulations
  - Consultation with mental health specialists when mental health conditions are present or suspected
  - Avoidance of coprescribing of sedative hypnotics
  - Coprescribing of naloxone

### Noninvasive Nonpharmacologic Treatments for Chronic Pain

- The effectiveness and comparative effectiveness (benefits and harms) of noninvasive nonpharmacologic treatments (exercise, mind-body practices, psychological interventions, multidisciplinary rehabilitation, mindfulness practices, musculoskeletal manipulation, physical modalities, and acupuncture) versus inactive treatments, usual care, no treatment, pharmacologic therapy, or selected active treatments (exercise [chronic pain conditions other than headache] or biofeedback [headache]), for the following conditions:
  - Chronic low back pain (KQ 1)
  - Chronic neck pain (KQ 2)
  - Osteoarthritis (knee, hip, hand) (KQ 3)
  - Fibromyalgia (KQ 4)
  - Chronic tension headache (KQ 5)

### Nonopioid Pharmacologic Treatments for Chronic Pain

- Effectiveness and comparative effectiveness (benefits [KQ 1] and harms [KQ 2]) of nonopioid pharmacologic agents (nonsteroidal anti-inflammatory drugs [NSAIDs], antidepressants, anticonvulsants, acetaminophen, muscle relaxants, memantine, topical agents, and cannabis) versus placebo or other nonopioid pharmacologic agents.

### Treatments for Acute Pain

- Effectiveness and comparative effectiveness (benefits and harms) of opioid therapy versus nonopioid pharmacologic therapy (acetaminophen, NSAIDs, skeletal muscle relaxants, benzodiazepines, antidepressants, anticonvulsants, and cannabis) or nonpharmacologic therapy (exercise, cognitive behavioral therapy, meditation, relaxation, music therapy, virtual reality, acupuncture, massage, manipulation or mobilization, and physical modalities); nonopioid pharmacologic therapy versus other nonopioid pharmacologic treatments or nonpharmacologic therapy; and nonpharmacologic therapy versus inactive treatments or usual care, for the following conditions:
  - Acute back pain (including back pain with radiculopathy) (KQ 1)
  - Acute neck pain (including neck pain with radiculopathy) (KQ 2)
  - Musculoskeletal pain not otherwise included in KQ 1 or KQ 2 (including fractures) (KQ 3)
  - Peripheral neuropathic pain (related to herpes zoster and trigeminal neuralgia) (KQ 4)
  - Postoperative pain (excluding inpatient management of pain following major surgical procedures (KQ 5)
  - Dental pain (KQ 6)
  - Kidney stones (including inpatient management) (KQ 7)
  - Sickle cell crisis (episodic pain) (KQ 8)

### Treatments for Acute Episodic Migraine

- Effectiveness and comparative effectiveness (benefits and harms) of the following:
  - Opioid therapy versus nonopioid pharmacologic therapy (acetaminophen, NSAIDs, triptans, ergot alkaloids, combination analgesics, muscle relaxants, antinausea medications, cannabis, or others [e.g., gepants]) or nonpharmacologic therapy (exercise, cognitive behavioral therapy, acupuncture, or others) (KQ 1)
  - Nonopioid pharmacologic therapy versus a different nonopioid pharmacologic therapy or nonpharmacologic therapy (KQ 2)
  - Nonpharmacologic therapy versus inactive treatments, usual care, or no treatment (KQ 3)

### Search Protocols

Complete methods and data, including detailed search protocols and inclusion and exclusion criteria, for the five AHRQ reports summarized here have been published (*1*–*5*). Study authors developed the search protocols using a standardized process with input from experts and the public. The review protocols were submitted for registration in the PROSPERO database before conducting the reviews. For each review, research librarians conducted searches on multiple electronic databases. For all reviews, searches were conducted on MEDLINE, Cochrane CENTRAL, and the Cochrane Database of Systematic Reviews; other databases that were used for one or more reviews (depending on the topic) were Embase PsycINFO, CINAHL, Scopus, and others. The searches were supplemented by a review of reference lists (including previous AHRQ and CDC reviews on these topics) (*6*–*8*) and gray literature sources. Searches were conducted in August or September 2019 for the chronic pain reviews and in July or August 2020 for the acute pain reviews.

### Summarizing the Evidence

The reviews categorized magnitude of effects for pain and function using the same system as previous AHRQ reviews (*6*,*9*). A small effect was defined for pain as a mean between-group difference after treatment of 0.5–1.0 points on a 0- to 10-point numeric rating scale (NRS) or visual analog scale (VAS) and for function as a standardized mean difference (SMD) of 0.2–0.5 or a mean difference of 5–10 points on the 0- to 100-point Oswestry Disability Index (ODI) (*10*), 1–2 points on the 0- to 24-point Roland-Morris Disability Questionnaire (RDQ) (*11*), or equivalent. A moderate effect was defined for pain as a mean difference of 10–20 points on a 0- to 100-point VAS (1–2 points on a 0- to 10-point NRS) and for function as an SMD of 0.5–0.8, or a mean difference of 10–20 points on the ODI, 2–5 points on the RDQ, or equivalent (*6*,*9*). Large or substantial effects were defined as greater than moderate. Similar thresholds were applied to other outcomes measured. Small effects using this system might not meet proposed thresholds for clinically meaningful effects (*12*). However, estimated minimum clinically important differences vary across studies, and the clinical relevance of effects classified as small might vary for specific patients depending on preferences, baseline symptom severity, harms, cost, and other factors (*13*,*14*). The reviews also evaluated results on the basis of dichotomous outcomes (e.g., likelihood of experiencing clinically meaningful improvement in pain or function, often defined as >30% or >50% improvement from baseline).

### Opioids for Chronic Pain

The AHRQ systematic clinical evidence review on opioids for chronic pain (*1*) updated the 2014 AHRQ report (*7*) and 2016 CDC update (*8*) and expanded on the previous reviews by adding evidence from randomized trials reporting short-term outcomes, including tramadol as an opioid intervention, addressing risks of coprescribing benzodiazepines or gabapentin, and addressing effects of co-use of cannabis.

### Effectiveness (Benefits and Harms)

For short-term (1 to <6 month) outcomes, based on over 70 placebo-controlled trials (evidence type 1), opioids were associated with beneficial effects versus placebo but mean differences were small: for pain, <1 point on a 0–10 scale and for function, a SMD of 0.22 or <1 point on the 0- to 10-point Brief Pain Inventory (*15*) interference scale and <1 point on the 0- to 24-point RDQ. Opioids were associated with a number of patients needed to treat (NNT) of approximately 6.7 to achieve one additional case of short-term pain relief (e.g., ≥30% improvement in pain). Analyses based on a combination of head-to-head (within study) comparisons as well as a meta-regression of placebo-controlled trials indicated an association between higher opioid dosage and greater short-term effects on pain that appeared to plateau at approximately 50 mg morphine equivalent dose (MME)/day (evidence type 2). Evidence also indicated that effects of opioids dissipate with longer duration of therapy. Opioids were associated with a small mean improvement in short-term sleep quality (evidence type 2) versus placebo and a small mean short-term improvement in Short-Form 36-item (SF-36) (*16*) mental health status (evidence type 1). Effects of opioids on short-term outcomes were generally consistent across opioid types (opioid agonist, partial agonist, or mixed medication agent). Effects on pain were somewhat greater for neuropathic than musculoskeletal pain (effects on pain approximately 0.5 point greater for neuropathic versus musculoskeletal pain on a 0–10 scale). Use of a crossover or enriched enrollment randomized withdrawal (EERW) design (a type of trial in which potential participants receive the study drug for a period in a prerandomization phase and only those who benefit from the drug and can tolerate the side effects continue in the trial, randomly assigned to continue on the study drug or placebo) (*17*) was associated with greater effects on pain than parallel group or non-EERW studies.

Opioids were associated with increased risk versus placebo for discontinuation because of adverse events (number of patients treated to cause one adverse event [number needed to harm, NNH 10], and increased risk for gastrointestinal events [NNH 7.1 for nausea, 14.3 for vomiting, and 7.1 for constipation], somnolence [NNH 11.1], dizziness [NNH 12.5], and pruritus [NNH 14.3]) (evidence type 1). Few serious adverse events and no difference between opioids versus placebo in risk were reported in the short-term trials (evidence type 2); however, serious adverse events were not well defined by the trials, the trials excluded patients at higher risk (e.g., those with a history of substance use disorder), and the trials were not designed to assess serious but less common harms such as overdose, opioid use disorder death, cardiovascular events, and fractures. EERW studies tended to report lower risk with opioids of discontinuation because of adverse events and gastrointestinal adverse events than non-EERW studies. Uncontrolled studies (studies without a nonopioid control group) were not included in the AHRQ review, although a recent systematic review with such studies found that rates of misuse ranged from 21% to 29% (95% CI: 13%–38%) and rates of addiction ranged from 8% to 12% (95% CI: 3%–17%), based on higher-quality observational evidence (*18*).

As in the 2014 AHRQ report and 2016 CDC update, the clinical evidence review identified no long-term (>1 year) randomized controlled trials (RCTs) of opioid therapy versus placebo. One new cohort study found long-term opioid therapy was not associated with improved pain, function, or other outcomes versus no opioids (*19*). New observational studies included in the new AHRQ review were consistent with the 2014 AHRQ report in finding an association between use of prescription opioids and risk for addiction, overdose, fractures, falls, and cardiovascular events (evidence type 3); a new study also found an association between opioid use and risk of all-cause deaths (*20*) (evidence type 4). New observational studies also were consistent with the 2014 AHRQ report in finding associations between higher dosages of opioids and risks for overdose, addiction, and endocrinological adverse events; new studies also found an association between higher dosage and increased risk for incident or refractory depression (*21*,*22*). Observational studies also indicated an association between coprescription of gabapentinoids (*23*–*25*) or benzodiazepines (*26*–*28*) and increased risk for overdose, with most pronounced risk occurring soon after initiation of these medications (evidence type 3). All observational studies were susceptible to residual confounding.

No differences were found across 16 trials between opioids versus nonopioids (most commonly NSAIDs, gabapentinoids, and nortriptyline) in short-term pain, function, health status or quality of life, sleep quality, or mental health outcomes (evidence type 1 for function; evidence type 2 for other outcomes), although opioids were associated with increased risk for short-term adverse effects (evidence type 1 or 2). Most trials were <6 months; one trial of patients with chronic low back pain or pain associated with osteoarthritis (mean pain intensity: 5.4 on a 0–10 scale at baseline) evaluated outcomes at 1 year (*29*). The trial found no differences between stepped therapy with opioids versus stepped therapy starting with nonopioids in function, sleep, or mental health outcomes; opioids were associated with slightly worse effects (by approximately 0.5 point on a 0–10 scale) on pain (evidence type 2). Although tramadol was an option in step 3 of the nonopioid stepped therapy arm, only 11% received tramadol; mean opioid doses for stepped opioid therapy and stepped therapy starting with nonopioids were 26 versus 1 MME/day, respectively, at 12 months.

Also, there were no differences between combination therapy versus a nonopioid alone in short-term effectiveness but increased risk for short-term adverse effects for combination therapy, on the basis of six trials (evidence type 3). Combination therapy was associated with a small (5–13 MME/day) opioid-sparing effect versus opioid therapy alone, with little effect on pain. All trials of combination therapy evaluated patients with neuropathic pain and primarily evaluated gabapentinoids or nortriptyline. Evidence on long-term effects of combination therapy versus an opioid or nonopioid alone was lacking.

### Opioid Dosing Strategies

Evidence on the effectiveness of different opioid dosing strategies remains limited. One trial included in the 2014 AHRQ report found no differences between a more liberal dosage escalation strategy versus maintenance of current dosages in pain, function, or discontinuation because of opioid misuse; however, the difference in opioid dosages between arms was small (52 versus 40 mg MMD/day) (*30*) (evidence type 3). No clear differences were found between short- versus long-acting opioids (evidence type 3) or between different long-acting opioids (evidence type 2) in pain or function; however, in most trials, dosages were titrated to achieve adequate pain control. Evidence on comparative risks of methadone versus other opioids and risk for overdose remains limited and inconsistent. Evidence on the benefits and harms of different methods for initiating and titrating opioids, scheduled and continuous versus as-needed dosing of opioids, use of opioid rotation, and methods for titrating or discontinuing opioids remains insufficient. The 2014 AHRQ report found buccal or intranasal fentanyl more effective than placebo or oral opioids for treatment of exacerbations of chronic pain, based on immediate effects (up to 2 hours after administration). None of the trials of buccal or intranasal fentanyl was designed to assess longer-term benefits or harms, and no new trials were identified for the 2020 systematic review. In 2007, the Food and Drug Administration released a public health advisory due to case reports of deaths and other life-threatening adverse effects in patients prescribed buccal fentanyl (*31*).

### Risk Mitigation Strategies

New evidence on the accuracy of risk prediction instruments was consistent with the 2014 AHRQ report, which found highly inconsistent estimates of diagnostic accuracy, methodological limitations, and few studies of risk assessment instruments other than the Opioid Risk Tool (*32*) and Screening and Opioid Assessment for Patients with Pain-Revised instrument (*33*) (evidence type 3). Evidence on the effectiveness of risk mitigation strategies also remains limited. One new observational study found that provision of naloxone to patients prescribed opioids in primary care clinics was associated with decreased likelihood of opioid-related emergency department visits; there were too few opioid poisoning deaths to assess effects on overdose mortality (evidence type 3) (*34*). Evidence on opioid tapering was largely limited to a trial that found a taper support intervention associated with better functional outcomes and a trend toward lower opioid doses versus usual opioid care (*35*) (evidence type 2). A cohort study found discontinuation of opioid therapy was associated with increased risk for overdose death versus continuation; however, there was no statistically significant difference in risk for all-cause deaths (*36*). Findings should be interpreted with caution because of potential confounding related to the reason for discontinuation.

No trial compared different rates of opioid tapering, although one observational study found an association between longer time to opioid discontinuation in patients on long-term, high-dosage opioid therapy and decreased risk of opioid-related emergency department visit or hospitalization (*37*) (evidence type 3). The review did not identify any study that evaluated the effectiveness of risk mitigation strategies, such as use of risk assessment instruments, opioid management plans, patient education, urine drug screening, PDMP data review, monitoring instruments in patients prescribed opioids, more frequent monitoring intervals, pill counts, abuse-deterrent formulations, or avoidance of coprescribing of benzodiazepines on risk for overdose, addiction, abuse or misuse.

Evidence on the effectiveness of interventions for opioid use disorder in patients with prescription opioid dependence or opioid use disorder was limited by such factors as small sample sizes, high attrition or crossover, and exclusion of patients with chronic pain.

### Noninvasive Nonpharmacologic Treatment for Chronic Pain

The AHRQ systematic clinical evidence review (*2*) focused on commonly encountered pain conditions and frequently used interventions. Selection of conditions for review was informed by stakeholder input.

### Benefits

**Chronic Low Back Pain**. The review found psychological therapies associated with small improvements versus usual care or an attention control for function and pain at short-, intermediate-, and long-term follow-up (evidence type 2). Exercise, low-level laser therapy, spinal manipulation, massage, yoga, acupuncture, and multidisciplinary rehabilitation were associated with improvements in function at short- and intermediate-term follow-up versus usual care, placebo, waiting list, or inactive therapies; effects on pain were small for all therapies except yoga, for which benefits were moderate (evidence type 2 at short term for exercise, massage, and yoga; evidence type 3 for others). Massage, mindfulness-based stress reduction, acupuncture, and multidisciplinary rehabilitation were associated with small short-term improvement in pain versus control (evidence type 2); exercise, low-level laser therapy, and yoga also were associated with small to moderate short-term improvement in pain, although evidence was not as strong (evidence type 3). At intermediate term, spinal manipulation, yoga, multidisciplinary rehabilitation (evidence type 2) and exercise and mindfulness-based stress reduction (evidence type 3) were associated with improved pain versus sham, usual care, or attention control; effects were small for all therapies except for yoga, for which effects were moderate. Compared with exercise, multidisciplinary rehabilitation was associated with small improvements in function and pain at short and intermediate terms (evidence type 2).

**Chronic Neck Pain.** The AHRQ systematic clinical evidence review found low-level laser therapy (evidence type 2) and massage (evidence type 3) associated with improved short-term function and pain for chronic neck pain. The magnitude of effect was moderate for low-level laser therapy and small for massage. Exercise was associated with small improvement in long-term function versus attention control (evidence type 3) and combination exercise was associated with improved short- and long-term function and short-term pain versus waiting list or attention control (evidence type 3). Acupuncture was associated with small improvements in short- and intermediate-term function versus sham, placebo, or usual care; however, there were no differences in pain versus sham acupuncture, an intervention meant to mimic acupuncture but without acupuncture effects (e.g., needles in nonacupuncture point, or nonpenetrating needles or pressure on acupuncture points) (evidence type 3). Pilates was associated with improved short-term function (small effect) and pain (large effect) versus acetaminophen (evidence type 3).

**Osteoarthritis Pain.** The AHRQ systematic clinical evidence review found that for knee osteoarthritis, exercise was associated with small improvements in short- and long-term function and pain versus usual care, no treatment, or sham (evidence type 2 for short-term and type 3 for long-term) and moderate improvement in intermediate-term pain and function (evidence type 3). For hip osteoarthritis, exercise was associated with small improvement in short-term function and pain versus usual care (evidence type 3). Functional improvement persisted at intermediate-term follow-up but pain improvement did not (evidence type 3).

**Fibromyalgia.** The AHRQ systematic clinical evidence review found exercise, mind-body practices, multidisciplinary rehabilitation, and acupuncture associated with small improvement in short-term function versus usual care or inactive treatments for fibromyalgia (evidence type 2 for acupuncture; evidence type 3 for others). At intermediate term, exercise, acupuncture, cognitive behavioral therapy, mindfulness-based stress reduction, myofascial release, and multidisciplinary rehabilitation were associated with improvements in function versus inactive treatments, usual care, or waiting list (evidence type 2 for exercise and acupuncture; evidence type 3 for others). Effects on intermediate-term function were moderate for cognitive behavioral therapy and small for the other therapies. At long term, multidisciplinary rehabilitation was associated with persistent small improvement in function versus usual care but not for pain (evidence type 3). Tai chi was associated with small improvement in function versus exercise at short- to intermediate-term follow-up (evidence type 3). Therapies associated with improved pain versus usual care, waiting list, no treatment, or inactive treatments were exercise (small effect, short and intermediate term; evidence type 2), cognitive behavioral therapy (small, short term; evidence type 3), mindfulness practices (small, intermediate term; evidence type 3), and multidisciplinary rehabilitation (small, intermediate term; evidence type 3).

**Chronic Tension Headache.** The AHRQ systematic clinical evidence review found spinal manipulation was associated with moderate improvement in short-term pain and small improvement in function versus usual care for chronic tension headache (evidence type 3). For other interventions, evidence was sparse, and the majority of trials had serious methodological limitations.

### Harms

Across conditions, data on harms of nonpharmacologic therapies were limited but no evidence suggested serious harms. Although reporting on harms was suboptimal, among studies that reported data, nonserious treatment-related adverse events (e.g., discomfort, soreness, bruising, increased pain, and worsening of symptoms) were infrequently reported, few withdrawals from nonpharmacologic therapies due to adverse events were reported, and no differences were found between comparison groups (either usual care or no nonpharmacologic therapy or another therapy) in the frequency of intervention-related adverse events or withdrawals (evidence type 2 or 3).

### Benefits

For neuropathic pain, the AHRQ systematic clinical evidence review (*3*) found anticonvulsants (gabapentin, pregabalin, and oxcarbazepine) were associated with small short-term improvement in pain versus placebo (evidence type 2), with no difference between pregabalin versus gabapentin enacarbil (evidence type 3). The antidepressant duloxetine was associated with small improvements in short-term pain, function, and quality of life versus placebo in patients with diabetic peripheral neuropathy (evidence type 2 for pain and quality of life; evidence type 3 for function). Tetrahydrocannabinol (THC) and cannabidiol (CBD) oral spray had inconsistent effects on pain in patients with multiple sclerosis or with allodynia (evidence type 3). Topical capsaicin was not associated with statistically significant effects on pain versus placebo, or effects were below the threshold for a small effect (evidence type 2).

For fibromyalgia, serotonin and norepinephrine reuptake inhibitor (SNRI) antidepressants milnacipran and duloxetine were associated with small, short- and intermediate-term improvements in pain and quality of life versus placebo; a small beneficial effect on function was only observed at short-term (evidence type 2). Anticonvulsants pregabalin and gabapentin were associated with small short-term improvements in pain and function versus placebo; there were no effects on quality of life (evidence type 2). Memantine was associated with moderate intermediate-term improvements in pain, function, and quality of life versus placebo (evidence type 3).

For osteoarthritis, NSAIDs were associated with small short-term improvement in pain (evidence type 2) and function (evidence type 1). Topical diclofenac was associated with small improvement in short-term pain (evidence type 2) and function (evidence type 3) versus placebo. Duloxetine was associated with small improvement in pain severity, function, and quality of life and moderate improvement in likelihood of a pain response (evidence type 1). Acetaminophen was not associated with improvement in pain or function versus placebo (evidence type 3).

For inflammatory arthritis, NSAIDs were associated with small improvements in short-term pain and function versus placebo (evidence type 2); effects on pain and function were small at intermediate-term follow-up (evidence type 3). At long-term follow-up, effects on pain were large, with no effects on function (evidence type 3).

For low back pain, duloxetine was associated with a small short-term improvement in pain intensity and likelihood of a pain response versus placebo; however, improvements in function and quality of life did not meet the threshold for small improvement (evidence type 2).

### Harms

Across all classes of nonopioid therapies, the AHRQ systematic clinical evidence review found that the incidence of serious adverse events was low; however, the trials were not designed to assess serious adverse events, and there were few serious adverse events (evidence type 3).

Antidepressants were associated with increased risk for withdrawal due to adverse events (WAE) versus placebo. SNRI antidepressants were associated with moderate to large increases in risk for nausea and excessive sweating (evidence type 2 or 3). Duloxetine was associated with a large, dose-dependent increase in sedation versus placebo (evidence type 2 or 3).

With regard to anticonvulsants, oxcarbazepine was associated with a large increase in risk for WAEs versus placebo (evidence type 2). Pregabalin and gabapentin were associated with moderate increased risk for WAEs (evidence type 2), with an association between higher dosages of pregabalin and increased risk. Pregabalin and gabapentin were associated with large increases in blurred vision, dizziness, weight gain, and cognitive effects (e.g., confusion) (evidence type 2). In addition, pregabalin was associated with large increases in risk for peripheral edema and sedation (evidence type 2).

NSAIDs were associated with increased risk for WAEs versus placebo; the magnitude was small for ibuprofen and diclofenac and moderate for naproxen (evidence type 2). There was no statistically significant increase in risk for any cardiovascular event for NSAIDs as a group; however, diclofenac was associated with a small increase in risk, particularly in the first 6 months, and with higher dosages (evidence type 2). Versus placebo, the risk for major coronary events was elevated with diclofenac and celecoxib (moderate effect) and with ibuprofen (large effect). For every 3,000 patients treated with diclofenac or celecoxib, there were an estimated three additional major coronary events. No difference was found in cardiovascular events between celecoxib versus nonselective NSAIDs in the intermediate or long term (evidence type 2). The risk for serious upper gastrointestinal events was increased with diclofenac (moderate effect) and ibuprofen or naproxen (large increase), particularly in the first 6 months of treatment (evidence type 1–2). In the intermediate term, diclofenac and naproxen were associated with large increase in risk for hepatic harms (evidence type 1–2).

Acetaminophen was not associated with increased risk for short- or intermediate-term WAEs versus placebo (evidence type 3). Capsaicin was associated with a large increase in risk for application site pain (evidence type 2) and a small increased risk for erythema (evidence type 3). Cannabis as oral dronabinol solution was associated with a large increase in risk for dizziness, and as THC or CBD was associated with a large increase in risk for WAEs, dizziness, and nausea (evidence type 3).

### Treatments for Acute Pain

The AHRQ systematic clinical evidence review (*4*) found that most trials of treatments for acute pain focused on effects on pain at short-term (up to 1 week) follow-up. Evidence was somewhat stronger for pharmacologic than nonpharmacologic therapies.

For acute surgical dental pain (evidence type 3) and kidney stone pain (evidence type 2), the AHRQ systematic clinical evidence review found that opioids were associated with small to moderate increases in pain or need for rescue medication use versus NSAIDs. Findings for postoperative pain were somewhat inconsistent. Although opioids were associated with increased likelihood of repeat or rescue medication use at 1 day to 1 week (evidence type 3), evidence on pain intensity was insufficient due to inconsistency. Results for postoperative pain were based on a small number of trials and pain related to a limited set of surgical procedures (most commonly cesarean section, anterior cruciate ligament reconstruction, knee arthroplasty, and cholecystectomy), limiting generalizability to other surgical procedures. Opioids were associated with increased risk for adverse events such as nausea, dizziness, and sedation versus nonopioid pharmacologic therapies (evidence type 2 or 3). The trials were not designed to assess serious adverse events, and few such events were reported. Evidence on opioids versus acetaminophen was somewhat mixed: for dental pain, the systematic clinical evidence review found opioids were associated with small improvement in pain outcomes on certain measures (evidence type 2) but for kidney stone pain, opioids were associated with a small increase in pain (evidence type 2). Evidence on NSAIDs versus acetaminophen was also somewhat mixed: for dental pain, evidence indicated that NSAIDs were associated with moderate to large decrease in pain (evidence type 2) but for kidney stone pain, evidence was insufficient. Evidence on nonopioid pharmacologic therapies other than NSAIDs or acetaminophen was very limited.

Evidence on nonpharmacologic therapies for acute pain was limited. For low back pain, the AHRQ systematic clinical evidence review found that heat therapy was associated with a moderate decrease in pain versus usual care or placebo at 1 day to <1 week and at 2 to <4 weeks (evidence type 2–3). For nonradicular low back pain, there might be no difference between spinal manipulation versus inactive controls (evidence type 2–3), although one trial of patients with radiculopathy found manipulation was associated with increased likelihood of improvement in pain at 2 to <4 weeks and at ≥4 weeks (evidence type 3) (*38*). Acupuncture was associated with moderate improvement in pain and function versus an NSAID for low back pain; however, findings were based on one trial that evaluated one session of acupuncture and a single dose of an NSAID (evidence type 3) (*39*). For postoperative pain, there was type 3 evidence that massage might have some effectiveness, with likely no difference between cold therapy versus no cold therapy, with the possible exception of decreased pain medication use at <1 week. Evidence supporting effectiveness of acupressure for acute musculoskeletal pain was limited (evidence type 3). Reporting of harms for nonpharmacologic therapies was suboptimal. However, the noninvasive nonpharmacologic therapies evaluated in the AHRQ systematic clinical evidence review were generally not thought to be associated with serious harms, and harms were few when reported.

Trials of opioid therapy for acute pain were not designed to evaluate effects on long-term use of opioids or outcomes such as misuse or development of opioid use disorder. Limited evidence from observational studies found that being prescribed an opioid for acute low back pain or after minor or elective surgical procedures was associated with increased likelihood of opioid use at longer term (e.g., 6 months or 1 year) follow-up (evidence type 3). Evidence on factors associated with opioid prescribing in patients with acute pain conditions was very limited and suggested that legislation mandating use of PDMP data before prescribing was not associated with decreases in opioid prescribing for low back pain or postoperative pain. No studies were identified that evaluated the accuracy or effectiveness of risk assessment instruments to inform use of opioids for acute pain.

### Treatments for Acute Episodic Migraine

The AHRQ review on treatments for acute episodic migraine (*5*) found limited evidence on the benefits and harms of opioids. The review found that opioids might be associated with decreased pain versus placebo but worse pain outcomes versus nonopioid pharmacologic therapy (evidence type 3). Most outcomes were assessed at short-term (2 hours or 1 day) follow-up. Opioids were associated with increased risk for adverse events, although evidence on serious adverse events was lacking. No studies were found on instruments for predicting opioid misuse, opioid use disorder, overdose, or risk mitigation strategies in patients prescribed opioids for migraine.

The AHRQ review found stronger (type 1 or 2) evidence supporting the effectiveness of multiple established nonopioid pharmacologic therapies for improving pain resolution in acute episodic migraine, including triptans, NSAIDs, dihydroergotamine, and ergotamine plus caffeine. Evidence also favored antiemetics versus placebo or no antiemetic but was more limited (evidence type 3). Newer treatments (calcitonin gene-related peptide [CGRP] antagonists [gepants], and lasmiditan [a 5-HT1F receptor agonist]) were associated with reduced pain and improved function versus placebo (evidence type 2 or 3). However, lasmiditan was associated with increased risk for serious adverse events (most commonly, dizziness; evidence type 3); evidence on serious adverse events of CGRP antagonists was insufficient.

Evidence on nonpharmacologic therapy for acute episodic migraine was sparse. Moderate evidence (evidence type 2) supported remote electrical neuromodulation. More limited evidence (evidence type 3) supported acupuncture, chamomile oil, external trigeminal nerve stimulation, and eye movement desensitization reprocessing. Evidence was insufficient to determine risk for serious adverse events with nonpharmacologic therapies for acute episodic migraine.

### Patient and Clinician Values and Preferences

**Opioids for Chronic Pain.** The contextual evidence review conducted for the 2016 CDC Opioid Prescribing Guideline (*8*) found data indicating that physicians frequently lacked confidence in their ability to safely prescribe opioids, predict or identify prescription medication misuse or opioid use disorder, or discuss these issues with their patients. Clinicians reported favorable beliefs and attitudes about effects of opioids on pain and quality of life; however, they also had concerns about risk for opioid use disorder and overdose yet did not consistently use risk mitigation strategies (e.g., use of PDMP data, urine toxicology testing, or opioid treatment agreements). Evidence on patient values and preferences was limited but indicated unfamiliarity with certain terms (“opioids”), more familiarity with the term “narcotics” but an association between “narcotics” and “addiction” or “abuse,” and concerns about addiction and abuse. Side effects such as nausea, constipation, and somnolence (rather than pain relief) accounted for most of the variation in patient preferences regarding use of opioids. Patients prescribed high-dose opioids reported reliance on opioids and ambivalence or uncertainty about benefits and side effects.

The AHRQ review identified new information on preferences and values. A survey of 961 clinicians found that 82% were reluctant to prescribe opioids and less than half (47%) expressed confidence in caring for patients with chronic noncancer pain (*40*). A total of 67% were aware of the 2016 CDC Opioid Prescribing Guideline and 55% were enrolled in the state PDMP; 2% always or frequently prescribed naloxone to patients on opioids, although results are difficult to interpret because the study did not specify whether patients met 2016 CDC Opioid Prescribing Guideline criteria for naloxone. Guideline awareness was associated with increased confidence in caring for patients with chronic pain. Other surveys found negative attitudes or concerns regarding prescription opioid use disorder but beliefs in potential effectiveness of opioids for treating pain and support for policies and guidelines aimed at mitigating risks, with increased confidence when following “best practices” (*41*–*43*).

Regarding patient preferences and values, a new systematic review found that among various opioid-related outcomes (effects), patients ranked pain relief, nausea, and vomiting as most important, followed by constipation (*44*). “Addiction” was only evaluated in two studies and rated as less important than pain relief. An online (non–peer reviewed) survey of approximately 3,000 patients 1 year after the release of the 2016 CDC Opioid Prescribing Guideline found that 84% reported more pain and worse quality of life and 42% said they had considered suicide; however, the survey did not attempt to sample patients with chronic pain using a rigorous methodological approach (*45*).

**Noninvasive Nonpharmacologic Treatments for Chronic Pain**. The contextual evidence review found that evidence on patient values and preferences related to noninvasive nonpharmacologic treatments for chronic pain was limited. A Gallup poll found that 78% of Americans preferred nonpharmacologic therapies (e.g., physical therapy and chiropractic care) to address pain over prescribed pain medication (*46*). Another survey indicated frequent use of complementary and integrative therapies for chronic pain (*47*).

Clinicians generally agreed with use of guideline-supported therapies and therapies supported by evidence, including nonpharmacologic therapies; clinicians also felt that treatments should be credible and individualized to the patient (*48*,*49*). Clinician concerns regarding nonpharmacologic treatments included costs and safety (*49*). Surveys indicated high support for use of exercise therapy, complementary medicine therapies, and psychological therapies (*50*–*52*); clinicians also supported chronic pain management informed by a biopsychosocial framework or using a multidimensional approach (*53*). Barriers to use of therapies included lack of knowledge or expertise and uncertainty regarding potential benefits (*48*,*50*,*52*–*55*).

**Nonopioid Pharmacologic Treatments for Chronic Pain.** The contextual evidence review found limited evidence on clinician and patient values and preferences related to nonopioid pharmacologic treatments. Evidence described variability in patient preferences regarding nonopioid pharmacologic treatments, interest in medical cannabis, cost as an important consideration, high priority on pain reduction as well as side effects and harms (including risk for opioid use disorder), and high value for having alternatives to opioids (*56*–*58*). A survey of pharmacists in Canada found that 38% agreed that nonprescription analgesics should be first line for chronic low back pain and 79% agreed that tricyclic antidepressants are effective for peripheral diabetic neuropathy (*59*).

**Treatments for Acute Pain.** The contextual evidence review found limited evidence suggesting variability in patient values and preferences regarding treatments for acute pain (*60*,*61*), with some evidence of high satisfaction when postoperative pain was managed using an opioid-sparing pathway (*62*). Also, there was variability in clinician values and preferences regarding acute pain treatments that were affected by clinical specialty, knowledge regarding effectiveness, and costs; negative attitudes toward acute pain conditions were associated with less likelihood of using or redosing opioids (*63*–*67*). A systematic review found inconsistent evidence that education increased clinician adherence with acute low back pain guideline recommendations in terms of referral rates to physiotherapy (*67*).

**Treatments for Acute Episodic Migraine.** The contextual evidence review found very limited evidence on clinician and patient values and preferences related to treatments for acute episodic migraine. One survey found that patients with headaches (primarily episodic or chronic migraine) prioritized efficacy of treatment over the safety or route of administration and preferred oral over parenteral medications (*68*). A survey of Canadian pharmacists found that 42% agreed that migraine patients should try nonprescription before prescription medications and 53% agreed that triptans should be reserved until failure of at least two other prescription medications (*59*).

### Costs and Cost-Effectiveness

**Opioid Therapy for Chronic Pain.** The contextual evidence review conducted for the 2016 CDC Opioid Prescribing Guideline estimated (on the basis of studies published after 2010) yearly direct and indirect costs related to prescription opioids at $53.4 billion for nonmedical use of prescription opioids; $55.7 billion for abuse, dependence (i.e., opioid use disorder), and misuse of prescription opioids; and $20.4 billion for opioid-related overdoses (*69*–*71*). In 2012, total expenses for outpatient prescription opioids were estimated at $9 billion, an increase of 120% from 2002 (*72*). On the basis of a large national sample of 2008 claims data, direct costs of opioids in patients with osteoarthritis were estimated at $287.40 per patient; however, there was wide variability in estimates (SD: $1,652.10) (*73*). One study estimated costs of urine toxicology testing (including screening and confirmatory tests) at $211–$363 per test (*74*).

The AHRQ report included data that estimated the total economic burden of fatal overdose, abuse, and dependence of prescription opioids in 2013 at $78.5 billion, with $28.9 billion related to increased health care and substance use disorder treatment costs (*75*). More recent data indicate that spending on opioid prescriptions peaked at $1.6 billion in 2009, with a decrease to $1.2 billion in 2016 (*76*). However, costs of treatment for opioid use disorder and overdose increased ($646 million in 2009 and $2.6 billion in 2016). Data also indicate that Medicaid spending on opioids has declined since 2014, although spending on buprenorphine (a partial opioid agonist often used to treat opioid use disorder) has increased (*77*), likely because of greater numbers of persons accessing medication and treatment for opioid use disorder.

No study was identified that formally evaluated the cost-effectiveness of opioid therapy versus no opioid therapy or nonopioid pharmacologic therapy for noncancer pain. A modeling study that estimated 80% of opioid overdose deaths to be attributable to illicit opioids projected that interventions targeting prescription opioid misuse (e.g., prescription monitoring programs) would decrease the number of opioid overdose deaths by 3.0%–5.3% (*78*). Also, there were no cost-effectiveness analyses of risk mitigation strategies in persons prescribed opioids for chronic pain. A systematic review that included 43 economic evaluation studies of treatments for opioid use disorder found evidence supporting the cost-effectiveness of methadone therapy, with less evidence for other opioid use disorder therapies (*79*). Additional analyses from the United Kingdom and California also found treatment for opioid use disorder to be cost-effective or cost-saving (*80*,*81*).

**Noninvasive Nonpharmacologic Treatments for Chronic Pain.** The contextual evidence review found that for nonpharmacologic treatments covered by commercial insurers, out-of-pocket costs ranged from $25 to $60 per visit ($150–$720 for a 6- to 12-visit course of therapy) (*55*). Studies found that a number of nonpharmacologic therapies were cost-effective for various chronic pain conditions. For osteoarthritis, cost-effective interventions (relative to a comparison such as no therapy or usual care) included exercise, acupuncture, and transcutaneous electrical nerve stimulation (*82*–*90*). For low back pain, cost-effective interventions included interdisciplinary rehabilitation, exercise, yoga, acupuncture, spinal manipulation, cognitive behavioral therapy, mindfulness-based stress reduction, biofeedback, and multidisciplinary rehabilitation (*91*–*99*). For neck pain, cost-effective interventions included manual therapy, physiotherapy, acupuncture, exercise, and spinal manipulative therapy (*94*,*100*–*104*). For fibromyalgia, cost-effectiveness analyses of nonpharmacologic therapies were very limited (*105*); however, certain evidence suggested that cognitive behavioral therapy dominated (associated with cost savings and greater benefits) pharmacologic therapy or usual care (*106*).

**Nonopioid Pharmacologic Treatments for Chronic Pain.** The contextual evidence review found certain evidence indicating that nonopioid pharmacologic therapies are cost-effective for chronic pain. For osteoarthritis and low back pain, there was evidence that nonopioid pharmacologic therapies (NSAIDs and duloxetine) are cost-effective versus opioids (*107*–*109*); studies also found NSAIDs, duloxetine, and pregabalin to be cost-effective versus usual care or no treatment (*108*,*110*–*112*). For neuropathic pain, cost-effective treatments included tricyclic antidepressants, duloxetine, pregabalin, and topical capsaicin or lidocaine (*113*–*126*). For fibromyalgia, cost-effective treatments included duloxetine, pregabalin, and amitriptyline, although analyses of relative cost-effectiveness among these therapies were inconsistent (*127*–*134*).

**Treatments for Acute Pain.** The contextual evidence review found limited evidence that exercise was cost-effective for acute low back pain and interdisciplinary rehabilitation cost-effective for low back pain that was identified as high risk for becoming chronic (*102*,*135*,*136*). Evidence that acetaminophen and spinal manipulation were not cost-effective for acute low back pain was limited (the acetaminophen analysis was based on a randomized trial that found acetaminophen to be ineffective for acute low back pain, and the spinal manipulation analysis was based on a cohort study that found manipulation for acute low back pain did not reduce follow-up visits or days of sick leave for low back pain) (*137*,*138*). One cohort study of patients with postsurgical pain found use of long-acting opioids within 30 days to be associated with greater costs of services ($11,900 versus $8,400; p<0.0001) (*139*).

**Treatments for Acute Episodic Migraine.** The contextual evidence review found that studies on costs and cost-effectiveness of treatments for acute episodic migraine focused almost exclusively on triptans. Triptans were consistently found to be associated with low costs per pain-free episode and other outcomes (e.g., migraine-disability days averted) (*140*–*148*). Triptans were dominant (more effective and less costly) over a fixed-dose combination of ergotamine tartrate plus caffeine (*149*).

### Opioid Treatments for Chronic Pain

To identify new evidence on opioid treatments for chronic pain that might have an impact on the conclusions or findings of the original (2020) systematic review, a series of three updates was conducted; searches for the final (third) update were conducted on March 16, 2022 (*150*). New evidence did not change the main findings of the original systematic review. For opioids versus placebo, updated meta-analyses that included three additional trials (*151*–*153*) reported a small reduction in pain intensity (mean difference: −0.78: 95% CI: −0.91 to −0.65), increased likelihood of experiencing >30% improvement in pain (RR: 1.33; 95% CI: 1.22–1.46), and small improvement in function (standardized mean difference: −0.21; 95% CI: −0.27 to −0.15), with estimates very similar to the original review. Findings for increased risk of opioids versus placebo of short-term harms (discontinuation because of adverse events, constipation, nausea, vomiting, dizziness, somnolence, and pruritus) also were unchanged. One new randomized trial (*154*) found transcutaneous electrical nerve stimulation to be associated with a small improvement in short-term function versus opioids and with decreased risk for any adverse event, nausea, constipation, and dizziness (strength of evidence: low); no study evaluated this comparison in the original review. No new randomized trials of opioids versus other nonpharmacological therapies or nonopioid medications were found. Two new cohort studies (*155*,*156*) found opioid dosage reduction or discontinuation to be associated with increased risk for mental health crisis events or fatal or nonfatal suicide attempts; however, evidence on the association between tapering or discontinuation and risk for overdose was inconsistent. The studies were not designed to evaluate the indication or circumstances for dosage reduction or methods used to support dosage reductions or discontinuation and had methodologic limitations, including potential for confounding. New evidence on long-term benefits and harms, risk mitigation strategies, dose-dependent risks of opioids, and management of opioid use disorder was limited; for all of these areas, findings with the addition of studies identified in the updates were consistent with the original report.

### Nonopioid Pharmacologic Treatments for Chronic Pain

To identify new evidence on nonopioid pharmacologic treatments for chronic pain that might have an impact on the conclusions or findings of the original (2020) systematic review, a series of three updates was conducted; searches for the final (third) update were conducted on April 1, 2022 (*157*). The addition of evidence identified during the updates did not change the main conclusions of the original review, which found nonopioid drugs (mainly SNRI antidepressants, pregabalin and gabapentin, and NSAIDs) to be associated with small to moderate improvements in short-term pain and function outcomes in patients with specific types of noncancer chronic pain. Evidence on intermediate- and long-term outcomes of nonopioid pharmacologic treatments for chronic pain remained limited. Findings after the addition of new studies were also consistent with the original review in finding nonopioid drugs to be associated with increased risk for class-specific harms (e.g., gastrointestinal events with NSAIDS), with certain patients withdrawing because of adverse events. For neuropathic pain, new evidence resulted in certain changes to strength of evidence of magnitude of effects assessments, including a change to low strength of evidence for small increased likelihood of experiencing a pain response with cannabis (RR: 1.30; 95% CI: 0.88–1.94; magnitude of reduction previously assessed as moderate) and large risk for sedation with cannabis (RR: 5.84; 95% CI: 1.90–17.92; previously insufficient evidence), due to the addition of one new randomized trial (*158*); strength of evidence was changed to low for no difference between gabapentin or pregabalin and duloxetine in pain intensity (previously insufficient evidence), due to the addition of two new randomized trials (*159*,*160*). An updated meta-analysis found capsaicin to be associated with a large increased risk for discontinuation because of adverse events (strength of evidence moderate) compared with placebo (previously no increase in risk), due to the addition of one new randomized trial (*161*), although the absolute number of participants who withdrew because of adverse events was small (<1%).

### Noninvasive Nonpharmacologic Treatments for Chronic Pain

To identify new evidence on noninvasive nonpharmacologic treatment for chronic pain that that might have an impact on the conclusions or findings of the original (2020) systematic review, a series of three updates was conducted; searches for the final (third) update were conducted in March 2022 (*162*). The addition of evidence identified during the updates did not change the main conclusions of the original review, which found exercise, multidisciplinary rehabilitation, acupuncture, cognitive behavioral therapy, mindfulness practices, massage, and mind-body practices to be associated with improved function, pain, or both, beyond the course of therapy for specific chronic pain conditions. Updated meta-analyses with the addition of new studies were conducted for low back pain (exercise, psychological therapies, manual therapy, mind-body practices, and acupuncture), neck pain (exercise), knee osteoarthritis (exercise, physical modalities [low-level laser therapy], ultrasound, and mind-body therapies), and fibromyalgia (exercise, mindfulness practices, acupuncture, and multidisciplinary rehabilitation). On the basis of the updated meta-analyses, the strength of evidence for mind-body therapies for knee osteoarthritis was upgraded to low for moderate improvement in pain and small improvement in function (previously insufficient evidence), due to the addition of one new trial (*163*); the strength of evidence for low level laser therapy for knee osteoarthritis was also upgraded to low for no difference in pain improvement and small improvement in function (previously insufficient evidence), due to the addition of one new trial (*164*). Otherwise, findings were unchanged from the original review. As in the original review, harms were poorly reported across interventions, although serious intervention-related adverse events were not identified.

### Treatments for Acute Pain

To identify new evidence on noninvasive nonpharmacologic treatment for chronic pain that might have an impact on the conclusions or findings of the original (2020) systematic review, a series of three updates was conducted; searches for the final (third) update were conducted on May 6, 2022 (*165*). The addition of evidence identified during the updates did not change the main conclusions of the original review. Specifically, opioid therapy was associated with decreased or similar effectiveness for pain versus an NSAID for surgical dental pain, kidney stone pain, and low back pain. New evidence was identified for low back pain (acupuncture), musculoskeletal pain (opioid versus acetaminophen, and topical ibuprofen versus capsaicin), postoperative pain (opioid versus NSAID and opioid versus acetaminophen; cold therapy; music therapy; abdominal binder; and transcutaneous electrical nerve stimulation), and dental pain (opioid versus NSAID, opioid versus acetaminophen, and NSAID versus acetaminophen). As in the original review, opioids and NSAIDs were more effective than acetaminophen for surgical dental pain and acute musculoskeletal pain, but opioids were less effective than acetaminophen for kidney stone pain. Opioids were associated with increased risk for short-term adverse events versus NSAIDs or acetaminophen, including any adverse event, nausea, dizziness, and somnolence. Serious adverse events were uncommon for all interventions; however, studies were not designed to assess risk for overdose, opioid use disorder, or long-term harms. Being prescribed an opioid for acute low back pain or postoperative pain was associated with increased likelihood of use of opioids at long-term follow up versus not being prescribed, on the basis of observational studies, although potential confounding could have had an impact on findings. Evidence on nonpharmacologic therapies for acute pain remained limited; however, heat therapy, spinal manipulation, massage, acupuncture, acupressure, a cervical collar, music therapy, transcutaneous electrical nerve stimulation, and exercise were effective for specific acute pain conditions. Evidence remained limited on the comparative effectiveness of therapies for sickle cell pain, acute neuropathic pain, neck pain, and management of postoperative pain after discharge; effects of therapies for acute pain on nonpain outcomes; effects of therapies on long-term outcomes, including long-term opioid use; and variations of benefits and harms of therapies among subgroups. A new finding from the updates was an association of preoperative education with decreased opioid use with similar or reduced pain intensity versus no preoperative education; this finding was based on three new trials (*166*–*168*) (no previous trials).

### Treatments for Acute Episodic Migraine

To identify new evidence on treatments for acute episodic migraine that might have an impact on the conclusions or findings of the original (2020) systematic review, a series of three surveillance reports was conducted; searches for the final (third) update were conducted on March 21, 2022 (*169*). The addition of new evidence identified in the updates did not change the main conclusions of the original review regarding the effectiveness for improving short-term (<1 day) pain and function of established pharmacological treatments (e.g., triptans, NSAIDs, antiemetics, and ergot alkaloids) and newer treatments (e.g., gepants and ditans); pharmacological treatments were associated with mild adverse events. Evidence on opioids for acute treatment of episodic migraine remain remained low or insufficient, and evidence on nonpharmacological treatments remained low, except for remote electrical neuromodulation (strength of evidence moderate). New evidence identified for the updates supported effectiveness of the calcitonin gene-related peptide eptinezumab (one new RCT) (*170*) and propofol (one new RCT) (*171*), occipital and supraorbital nerve blocks (two new RCTs) (*172*,*173*), transcranial stimulation (one new RCT) (*174*), and inhaled oxygen (one new RCT) (*175*) in acute treatment of episodic migraine (moderate strength of evidence for eptinezumab; otherwise low strength of evidence).

### A Note on Historically Used Terms

Historically, terms such as “abuse,” “drug abuse,” and “opioid abuse” have been used in research and diagnostic terminology. For example, opioid use disorder, defined in the *Diagnostic and Statistical Manual of Mental Disorders,* *Fifth Edition* (DSM-5) as a problematic pattern of opioid use leading to clinically significant impairment or distress (*176*), was previously referred to as opioid abuse or opioid dependence in the *Diagnostic and Statistical Manual of Mental Disorders*, *Fourth Edition* (DSM-IV) (*177*,*178*). However, more recent research indicates that use of terms such as “abuse” negatively affects perceptions and judgments about persons with drug use and substance use disorders (*179*–*181*). In the *CDC Clinical Practice Guideline for Prescribing Opioids for Pain — United States, 2022*, “abuse” is sometimes used to accurately reflect underlying sources or to report findings from research conducted using this terminology; however, terms such as “drug use” or “opioid use” typically are used to describe behaviors, and terms such as “substance use disorder” or “opioid use disorder” are used when discussing relevant diagnoses (*176*).
